# Assessing Students’ Translation Competence: Integrating China’s Standards of English With Cognitive Diagnostic Assessment Approaches

**DOI:** 10.3389/fpsyg.2022.872025

**Published:** 2022-03-31

**Authors:** Huan Mei, Huilin Chen

**Affiliations:** ^1^School of English Studies, Shanghai International Studies University, Shanghai, China; ^2^School of Education, Shanghai International Studies University, Shanghai, China

**Keywords:** translation competence assessment, cognitive diagnosis, China’s Standards of English, fine-grained diagnostic feedback, translation teaching and learning

## Abstract

While translation competence assessment has been playing an increasingly facilitating role in translation teaching and learning, it still failed to offer fine-grained diagnostic feedback based on certain reliable translation competence standards. As such, this study attempted to investigate the feasibility of providing diagnostic information about students’ translation competence by integrating China’s Standards of English (CSE) with cognitive diagnostic assessment (CDA) approaches. Under the descriptive parameter framework of CSE translation scales, an attribute pool was established, from which seven attributes were identified based on students’ and experts’ think-aloud protocols. A checklist comprising 20 descriptors was developed from CSE translation scales, with which 458 students’ translation responses were rated by five experts. In addition, a Q-matrix was established by seven experts. By comparing the diagnostic performance of four widely-used cognitive diagnostic models (CDMs), linear logistic model (LLM) was selected as the optimal model to generate fine-grained information about students’ translation strengths and weaknesses. Furthermore, relationships among translation competence attributes were discovered and diagnostic results were shown to differ across high and low proficiency groups. The findings can provide insights for translation teaching, learning and assessment.

## Introduction

In translation studies, translation competence assessment is a widely-discussed topic that has gained continuous scholarly attention (Adab, 2000; Orozco and Hurtado Albir, 2002; Way, 2008; Angelelli, 2009; Galán-Mañas and Hurtado Albir, 2015; Hurtado Albir, 2015). One remarkable progress, as can be seen during the past decades, is that assessment has been no longer merely about a way of measurement, it has also served as a tool to facilitate translation teaching and learning. For example, the practice of traditional assessment that only provided one total score has been supplemented with or sometimes even replaced by the use of various assessment instruments such as rubrics or scales to guide students’ autonomous learning or teachers’ feedback-giving (e.g., Presas, 2012; Galán-Mañas, 2016). However, on one hand, most of these instruments, if not all, were self-made while lacking rigorous development and validation procedures. On the other hand, feedback through neither way of assessment could convey fine-grained diagnostic information about students’ translation competence.

Even though quite a few language proficiency scales have started to arise since last century, notably, Interagency Language Roundtable Scale (ILR), Canadian Language Benchmarks (CLB), Common European Framework of Reference for Languages (CEFR), and American Council for the Teaching of Foreign Languages (ACTFL), only ILR included translation competence descriptors which were primarily designed for government’s language service management rather than for translation teaching and learning. Presumably because of the growing frequency of international exchanges and cooperation, recent years have begun to witness the emerging role of translation competence in language proficiency scales. For example, Council of Europe added translation competence descriptors to its new version of Council of Europe (2018). Also, China’s Standards of English (CSE; National Education Examinations Authority, 2018), the Chinese counterpart to CEFR, included translation competence scales as one of its major constitutes. Unlike those self-made ones in previous studies, these two scales have experienced rigorous development processes and were therefore of higher quality to evaluate students’ translation competence. Take CSE for example, guided by Bachman and Palmer (1996, 2010) communicative language ability (CLA) model while situated in Chinese English teaching and learning context, the CSE project team has spent more than 3 years collecting and validating descriptors from 1,60,000 Chinese EFL learners and teachers nationwide.

The second problem, through no fault of teachers’ own, lies in the inadequacy of previous measurement approaches in helping produce students’ mastery information on each translation sub-competence or sub-skills. Hurtado Albir and Pavani (2018) tried to design a multidimensional assessment scheme for the purpose of providing data about students’ different translation sub-competences, however, their proposal involved more than 20 tasks (with each task corresponding to each sub-competence), making it time-consuming and therefore unrealistic to implement in most assessment settings. In this case, a more useful approach that can delve into specific information about students’ translation competence while using a moderate amount of assessment tasks is called for. Fortunately, a new paradigm of measurement approach—cognitive diagnostic assessment (CDA), is found to meet such needs. Different from traditional measurement theories such as classical test theory (CTT) or item response theory (IRT), CDA can provide diagnostic feedback through fine-grained reporting of students’ skill mastery profiles (Tatsuoka, 1983; Shohamy, 1992; DiBello et al., 1995) “with a limited number of items/tasks available in a test form” (Sawaki et al., 2009, p. 192).

Given that such scales as CSE can overcome the shortcomings of previous assessment instruments by providing reliable and valid translation competence descriptors while CDA can outperform traditional measurement approaches in generating fine-grained information about certain competence with comparatively limited tasks, it is our assumption that the integration of the two is much likely to provide solutions to the aforementioned problems. Focusing on Chinese/English language pairs, this study is therefore aimed at investigating the feasibility of integrating CSE with CDA approaches to provide diagnostic information about students’ translation competence.

## Literature Review

### Cognitive Diagnostic Assessment

“CDA is designed to measure specific knowledge structures and processing skills in students so as to provide information about their cognitive strengths and weaknesses” (Leighton and Gierl, 2007, p. 3). Generally speaking, six steps are involved in the application of CDA approaches, namely, describing assessment purposes, identifying attributes, constructing a Q-matrix, validating the Q-matrix empirically, evaluating the selected model, and communicating diagnostic feedback (Fan and Yan, 2020). At the very beginning, the question of “what to assess” (theoretical construct) should be answered. Next, by seeking guidance from various relevant sources such as test specifications, content domain theories, analysis of item content, think-aloud protocol analysis of examinees’ test-taking process, and other relevant research results (Buck and Tatsuoka, 1998; Leighton et al., 2004; Leighton and Gierl, 2007), the construct is operationalized into a set of cognitive attributes, namely, specific knowledge, skills or strategies involved in completing the test tasks (Birenbaum et al., 1993; Buck et al., 1997). Then, a Q-matrix, “specifying the relationship between attributes and test items” (Jang, 2009a, p. 211), is constructed by “content experts such as language assessment specialists, language teachers, and applied linguists” (Lee and Sawaki, 2009a, p. 170). Last, cognitive diagnostic models (CDMs) are compared and selected to conduct cognitive diagnostic analysis and then generate diagnostic feedback. So far, there have been a vast array of CDMs among which some typical ones are the linear logistic model (LLM; de la Torre and Douglas, 2004), the deterministic input, noisy “and” gate model (DINA; Junker and Sijtsma, 2001), the reduced reparameterized unified model (RRUM; Hartz, 2002), the deterministic input, noisy “or” gate model (DINO; Templin and Henson, 2006), the additive cognitive diagnostic model (ACDM; de la Torre, 2011) and the generalized DINA model framework (G-DINA; de la Torre, 2011). Various as they are, all CDMs can be classified into compensatory, non-compensatory and saturated (general) models. Compensatory models (e.g., LLM, DINO, and ACDM) assume that mastery of one attribute can compensate for non-mastery of other attributes needed to answer an item correctly. By contrast, non-compensatory models (e.g., DINA and RRUM) assume that examinees should master all the attributes required by the item so as to produce correct answers. Saturated models (e.g., G-DINA) allow for both compensatory and non-compensatory relationships within the same test, however, such CDMs employ more complex parameterization and thus require a larger sample size in order to provide accurate estimates (de la Torre and Lee, 2013).

### Cognitive Diagnostic Assessment in Language Testing

Despite its popularity in the psychometric community for quite a long time, cognitive diagnostic research didn’t show its presence in language testing until the 1990s (Sheehan et al., 1993; Buck et al., 1997; Buck and Tatsuoka, 1998). Since then, a plethora of studies have been conducted, the majority of which were devoted to exploring the feasibility and validity of retrofitting existing non-diagnostic language tests for cognitive diagnostic purposes. Specifically, they have investigated the extent to which CDMs could fit the response data and then generated fine-grained information about test-takers’ language strengths and weaknesses. Taking a step forward, quite a few studies have explored the extent to which diagnostic results could help discover the relationships among attributes (e.g., Chen and Chen, 2016a; Ravand and Robitzsch, 2018; Du and Ma, 2021) and could differ across different proficiency groups (e.g., Kim, 2010, 2011; Fan and Yan, 2020). Among all those studies, a notable phenomenon is that assessment of receptive language skills such as reading and listening (e.g., von Davier, 2008; Jang, 2009b; Lee and Sawaki, 2009b; Kim, 2015; Chen and Chen, 2016b; Yi, 2017; Aryadoust, 2021; Dong et al., 2021; Toprak and Cakir, 2021; Min and He, 2022) gained much more attention than that of productive ones such as writing (Kim, 2010, 2011; Xie, 2017; Effatpanah et al., 2019; He et al., 2021). “One possible reason is that different test methods are used to assess these two types of skills” (He et al., 2021, p. 1). For the former, selected-response tests are often used, producing answers that can be scored with 1 (right) or 0 (wrong), which can serve as the response data directly analyzed by CDMs. The latter, however, is usually assessed with constructed-response tasks, meaning that it is difficult to score the answers (e.g., an essay) with either 1 or 0. To tackle this problem, Kim (2010, 2011) designed a diagnostic checklist composed of 35 writing ability descriptors and asked experts to use this checklist to assess examinees’ essays by rating 1/0 on each descriptor, the rating results of which then served as the writing response data analyzed by CDMs. Kim’s study provided methodological references for cognitive diagnostic research conducted with constructed-response tests, but descriptors in the study needed further amelioration in that they were sourced merely from a few teachers’ verbal reports. He et al. (2021) made an improvement in this regard by using descriptors selected from CSE. One slight flaw in their study, however, was that only one type of writing activity was involved, which, to some degree, restricted the inferences of test-takers’ writing competence.

Another phenomenon worth noting is that while CDA approaches were shown to be suitable and effective for language assessment, performances of different CDMs varied. The G-DINA model turned out to be the most widely-used or the best-fitted model in diagnosing receptive language skills (e.g., Chen and Chen, 2016b; Ravand, 2016; Ravand and Robitzsch, 2018; Javidanmehr and Sarab, 2019) given that “it is sensitive to the integrative nature of comprehension skills” (Chen and Chen, 2016a, p. 1051). However, no optimal model has been found in writing competence diagnosis due to the limited number of relevant research. Among the very few studies, three models have been found with the best model fit data including RRUM (Kim, 2010, 2011; Xie, 2017), ACDM (Effatpanah et al., 2019), and LLM (He et al., 2021). To the best of our knowledge, it seems that cognitive diagnostic studies on translation competence are not yet available. To solve the problems existing in translation competence assessment mentioned earlier, it is imperative that such a research gap should be filled. But before that, the foremost thing is to determine the construct of translation competence.

### Construct of Translation Competence

The construct of translation competence has caught continuous scholarly attention over the past decades (e.g., Cao, 1996; PACTE, 2003, 2005, 2008, 2015; Pym, 2003; Göpferich and Jääskeläinen, 2009), among which the empirical research results from PACTE have received considerable support. According to PACTE, translation competence is comprised of bilingual, extralinguistic, knowledge of translation, instrumental and strategic as well as psycho-physiological components. Based on PACTE’s translation competence model while taking into consideration the research findings on Chinese/English translation competence (e.g., Yang, 2002, 2014), the translation team of the CSE project defined translation competence as “the written language transference ability demonstrated by language learners and users in the participation of intercultural and *trans*-lingual activities” (National Education Examinations Authority, 2018, p. 108). Following this construct, they established a descriptive parameter framework of CSE translation scales composed of four major parameters, namely, translation activities, translation strategies, translation knowledge, and typical translation features (see Table 1). Under each parameter exists several subparameters with each corresponding to one subscale in CSE translation scales. So far, subscales related to the first two parameters have already been unveiled. For each subscale, there are five proficiency levels in which levels 5 and 6 are generally aligned with novice translation learners while levels 7, 8, and 9 with advanced ones. Among those parameters, translation activities can be helpful in selecting appropriate assessment tasks. For the other three ones, operationalized as they are, not all of them are suitable enough to serve the intended purpose of cognitive diagnosis. In particular, typical translation features, as the name suggests, are more concerned with the general criteria of translation quality rather than specific knowledge or skills. In this case, it is suggested that further refinement of this parameter framework is needed.

**TABLE 1:** Descriptive parameter framework of CSE translation scales.

First-level parameter	Second-level parameter	Third-level parameter
Translation	Translation activities	Description
		Narration
		Exposition
		Argumentation
		Instruction
		Interaction
	Translation strategies	Planning
		Execution
		Appraising and compensation
	Translation knowledge	Theoretical knowledge
		Practical knowledge
		Professional knowledge
	Typical translation features	Accuracy
		Completeness
		Appropriateness
		Fluency
		Standardization

*Bai et al. (2018, p. 107).*

### Refinement of the Parameter Framework of China’s Standards of English Translation Scales

Following the definition of cognitive attributes, it can be seen that both translation strategies and translation knowledge can meet the requirement. In other words, if we intend to conduct cognitive diagnostic assessment of translation competence, such subparameters as strategies of planning, execution, appraising and compensation, as well as theoretical, practical and professional knowledge are suitable to serve as cognitive attributes. By way of contrast, subparameters of typical translation features are neither strategies nor knowledge and thus need to be refined. To this end, document analysis was conducted to search for subskills corresponding to those subparameters, i.e., accuracy, completeness, appropriateness, fluency and standardization. By perusing translation competence descriptors in CSE and referring to the syllabus for major English tests (with translation section) and translation tests administrated in China including College English Test-Band 4 and 6 (CET 4 and 6), Test for English Majors-Band 8 (TEM 8), and China Accreditation Test for Translators and Interpreters Level 2 and 3 (CATTI 2 and 3), six major subskills were extracted, namely, conveying key information, conveying details with accurate wording, conforming to language norms, conforming to language habits, reproducing styles and optimizing logical structures. All those subskills can be classified into the five typical translation features, that is, conveying key information, conveying details with accurate wording and reproducing styles can fall into the category of “accuracy” and “completeness” while conforming to language norms, conforming to language habits and optimizing logical structures belong to “standardization,” “appropriateness,” and “fluency” respectively. Together with the subparameters of translation strategies and knowledge, an attribute pool of translation competence was then established to serve as the framework of reference for attribute selection (see Table 2). For the definition of those attributes, the first six ones were from the translation team of the CSE project (Bai et al., 2018), while the last six ones were a synthesis of test syllabus content, CSE translation competence descriptors and the definition of typical translation features.

**TABLE 2:** The attribute pool of translation competence.

Attribute	Definition
Using the strategy of planning	Making plans for the translation process and translation activities according to the purpose of translation.
Using the strategy of execution	Making use of translation techniques (e.g., amplification, omission, and conversion) to solve translation problems.
Using the strategy of appraising and compensation	Evaluating and monitoring the translation process and products so as to compensate for translation deficiencies in a timely manner by using appropriate translation compensation techniques.
Mastering theoretical knowledge	Understanding the nature, basic concepts, theories and history of translation.
Mastering practical knowledge	Applying theoretical knowledge of translation to specific translation practice activities.
Mastering professional knowledge	Mastering knowledge of translation industry norms, professional conduct, etc.
Conveying key information	Understanding and communicating main messages in a faithful, complete and correct manner without obvious wrong translation and loss of information.
Conveying details with accurate wording	Using appropriate words to express meaning in an accurate way.
Conforming to language norms	Conforming to relevant standards of the target language (grammatical rules, terminology, units of measurement, etc.).
Conforming to language habits	Conveying messages in accord with language and cultural habits of the target language.
Reproducing styles	Reproducing the original rhetorical and stylistic features.
Optimizing logical structures	Reproducing the original text structures in a logical and coherent way.

*Sources. Bai et al. (2018); China’s Standards of English Language Ability (National Education Examinations Authority, 2018); Syllabus for CATTI (CATTI Center, 2020a,b); Syllabus for CET (National College English Testing Committee, 2016); Syllabus for TEM8 (National Advisory Committee for Foreign Language Teaching, 2004).*

So far, we have identified the problem existing in translation competence assessment, which is also shown to be a research lacuna in cognitive diagnostic research in language testing. Moreover, we have found that CSE could provide a scientific framework of reference for the application of CDA approaches to translation competence assessment in such aspects as selection of tasks and attributes, development of diagnostic checklists, etc. This study, therefore, was aimed at investigating the feasibility of integrating CSE with CDA approaches to assess students’ translation competence. To this end, this study was guided by three research questions: (1) To what extent can CDMs fit the data and then generate fine-grained information about examinees’ translation strengths and weaknesses? (2) To what extent can diagnostic results help discover the relationships among translation competence attributes? (3) To what extent can diagnostic results differ across high and low translation proficiency groups?

## Methodology

### Instrument

As with the common practice in current cognitive diagnostic studies, this study retrofitted a non-diagnostically designed test for cognitive diagnostic output. The translation responses were collected from a school-based English proficiency test (SEPT) administered in a leading foreign studies university in China. The SEPT, administered in paper-and-pencil format at the end of each semester, is designed for non-English major undergraduates. As for the content of the SEPT, language skills and knowledge such as listening, reading, writing, vocabulary and grammar are often covered. In particular, translation is only included in the SEPT targeted at juniors presumably because the translation course is not open to them until the third school year. For the development of the SEPT administered in January 2021, the two researchers were responsible for the selection of materials for the translation section. Following the descriptive parameter framework of CSE translation scales, we tried to cover as many translation activities as possible. Considering our subjects were novice translation learners, short texts with simple language and familiar topics mainly from the retired CET translation sections were chosen with reference to descriptors from levels 5 and 6 in the CSE translation scales. With careful selection and discussion, the translation section of the SEPT was finally developed which consisted of four tasks covering five major types of activities, namely, description, narration, exposition, argumentation and instruction. The first two tasks were in English (111 words in total) designed to assess students’ English–Chinese translation competence while the last two were in Chinese (171 words in total) used to evaluate students’ Chinese–English translation competence. Based on the requirement for translation speed in CET (280 ∼ 320 words per hour), a total of 60 min were set for the completion of the translation section. After the test, 510 translation responses were obtained, out of which 458 ones were left in this study after those with a considerable amount of incomplete answers (with a proportion of more than 15% of the original content untranslated) were screened out.

### Participants

Twelve juniors (10 females and 2 males) majoring in Chinese/English translation volunteered to help identify the attributes involved in this study. Averaged at 21 years old, these students had learned translation for 2.5 years.

Seven domain experts (four males and three females) were recruited to help identify the attributes, develop the diagnostic checklist and construct the Q-matrix, and five of them also participated in the rating process in this study. We selected experts who (a) acquired a master’s or doctoral degree in translation. (b) Had hands-on experience as translators or translation teachers. (c) Had previous experience as scorers in large-scale language tests. Averaged at 30 years old, the seven experts involved in this study had an average of 5.2 years’ translation experience. All of them had acquired a master’s degree in translation and had served as scorers in such tests as TEM and CET. In addition, four of them were faculty members or Ph.D. candidates in the translation program, the other three were professional translators.

### Procedures

Somewhat different from the steps mentioned by Fan and Yan (2020) in diagnosing receptive language skills, the procedures in this study bore a strong resemblance to those in diagnosing writing skills (Kim, 2010, 2011; Xie, 2017; Effatpanah et al., 2019; He et al., 2021) which included five major stages: identifying the attributes, developing the diagnostic checklist, constructing the Q-matrix, rating the translation responses and selecting the CDM.

#### Identifying the Attributes

In the literature review, we have established an attribute pool of translation competence comprising 12 attributes. In order to identify those involved in this study and at the same time to check whether there existed any other attribute, think-aloud protocols were conducted among students and experts. To be more specific, 12 juniors majoring in Chinese/English translation were asked to give a retrospective verbal report on the process of translating the four texts. Translation majors were chosen because “their proficiency level is relatively higher than that of the subjects and thereby can provide more comprehensive information about the subskills involved in the test” (Zhang and Shi, 2020, p. 81). In addition, seven experts were asked to verbalize how they evaluated 10 translation responses that were randomly chosen from 458 responses. A total of 85 and 158 min’ verbal reports were collected from students and experts, respectively, transcribed into about 18,000 and 36,500 words (Chinese) respectively.

By analyzing and coding the transcription, the two researchers consistently identified seven major attributes in this study including conveying key information (A1), conveying details with accurate wording (A2), conforming to language norms (A3), conforming to language habits (A4), reproducing styles (A5), optimizing logical structures (A6) and using the strategy of execution (A7). For detailed transcription, please refer to the Supplementary Material. Because of the limited space, only some typical quotes extracted from the verbal reports were presented in Table 3 to demonstrate how we identified the seven attributes in our study. For example, expert No. 7 pointed out that “the student didn’t fully understand the original information and tended to translate the text based on their own interpretation, which caused a lot of wrong translations,” it was found that what he said was consistent with the definition of the attribute “conveying key information,” then, we identified A1 as one of the attributes in our study.

**TABLE 3:** Selected quotes from students’ and experts’ verbal reports.

Attribute	Selected quotes
A1	E7: Obviously, this student didn’t fully understand the original information and tended to translate the text based on their own interpretation, which caused a lot of wrong translations. S7: I didn’t quite understand the sentence “we do not believe that climate change is a certainty” which is a little bit contradictory to common sense, so I was not sure whether my word-for-word translation was correct or not…
A2	E6: Some of the words in the translated text were not accurate enough, for example, “affair” could not be used to replace the meaning of “marriage.” S12: It was challenging to choose an accurate and appropriate expression of “冲”, crash? flock? or rush? I couldn’t find the one that I felt satisfied with.
A3	E5: Grammatical and spelling mistakes were common in this translation work such as the wrong spelling of “flew” or a lack of articles in front of singular nouns (house, car, etc.).
A4	E2: The translated text was awkward and did not look like Chinese because all the passive voice in the original (English) text have been followed rigidly. S6: Given that verbs are used more frequently in Chinese than in English, I chose to convert nouns into verbs in my translation.
A5	E3: This student failed to take into account the style of the original text (a notice posted up in the library) and therefore could not achieve pragmatic effects. S9: This text seemed to be very official, so I tried to use formal words to keep consistent with the tone in the original text.
A6	E4: This student failed to reproduce the logical relationship existing in the original text such as the causal relationship in the third translation task. S2: I did not quite understand the logic within the second sentence, so I felt there must be some problems with my translation.
A7	E1: The technique of “explicitation” should be used to further explain the word “needs” to help target readers better understand this message. S5: To make the English word “it” more specific in Chinese, I chose to use the technique of “conversion”.

*“E,” “S” mean expert and student, respectively.*

#### Developing the Diagnostic Checklist

Three steps were involved in developing the diagnostic checklist. Firstly, based on the translation activities involved in the four translation tasks and the English proficiency levels of the examinees, a total of 46 descriptors mainly from the six subscales (description, narration, exposition, argumentation, instruction, and the strategy of execution) at levels 5 and 6 were selected to form an initial descriptor pool. Then, by analyzing the content of the four translation tasks, the two researchers chose 22 descriptors that were of direct relevance to the tasks from the descriptor pool. Next, a discussion among the seven experts was conducted to judge whether these 22 descriptors were suitable, the result of which was that controversy on 2 descriptors was unsolved and thus deleted. To call on He et al. (2021) suggestion, for the remaining 20 descriptors, the researchers made some modifications in due courses to tailor them to specific translation tasks for the convenience of experts’ ratings. Table 4 shows the final diagnostic checklist of 20 descriptors in which D1–D3, D4–D8, D9–D14, and D15–D20 belong to task 1, task 2, task 3, and task 4, respectively.

**TABLE 4:** The diagnostic checklist of 20 descriptors.

Task	No.	Descriptor
One	D1	Can translate short argumentative texts on common themes, conveying the arguments and reasoning.
	D2	Can accurately convey detailed information in the argumentative texts.
	D3	Can change wording and sentence structures based on the writing styles of the original, ensuring stylistic consistency between the original and the translation.
Two	D4	Can translate notices and posters used in everyday life, conveying the key information.
	D5	Can accurately convey detailed information in the notice.
	D6	Can convert passive voice into active voice as needed in the notice.
	D7	Can change wording and sentence structures based on the writing styles of the original, ensuring stylistic consistency between the original and the translation.
	D8	Can add words or phrases implied in the original, making the translation coherent and intelligible.
Three	D9	Can translate short popular science articles, conveying the key information.
	D10	Can convey detailed information in the exposition.
	D11	Can properly translate expressions for a series of nouns in accordance with the grammatical rules of the translation.
	D12	Can flexibly adjust the word order according to the way of expression in English.
	D13	Can flexibly use translation skills such as omissions to remove repetitions in the original.
	D14	Can add conjunctions indicating logical connections implied in the original according to English sentence patterns.
Four	D15	Can translate simple documentary texts, reproducing the courses of events.
	D16	Can translate accurately reproduce the original details in scenes of events and activities.
	D17	Can use transliteration to translate proper nouns, such as names of persons and places.
	D18	Can properly translate expressions for space and time in accordance with the grammatical rules of the translation.
	D19	Can choose proper sentence patterns to reproduce the tone in the original.
	D20	Can convert diverse Chinese clauses or sentences into English compound sentences, non-finite verb phrases, or prepositional phrases, making the translation compact and concise.

#### Constructing the Q-Matrix

Seven experts were asked to identify the relationship between each descriptor and the seven attributes. For each descriptor, if a certain attribute was thought to be measured, then “1” was coded, otherwise, “0” was coded. Putting their coding results together, we coded “1” for those relationships on which four or more experts reached consensus (Meng, 2013; Du and Ma, 2021), otherwise, we coded “0.” In such a way, the Q-matrix was constructed (see Table 5). As shown, out of the 20 descriptors, 11 descriptors were coded as measuring one attribute and 9 ones measuring more than two attributes. The times of measurement for each attribute ranges from 3 to 9, meeting the requirement that attributes should be measured at least 3 times in order to produce stable results (Buck and Tatsuoka, 1998; Hartz, 2002).

**TABLE 5:** The Q-matrix.

AttributeDescriptor	A1	A2	A3	A4	A5	A6	A7
D1	1	0	0	0	0	0	0
D2	0	1	0	0	0	0	0
D3	0	0	0	0	1	0	1
D4	1	0	0	0	0	0	0
D5	0	1	0	0	0	0	0
D6	0	0	0	1	0	0	1
D7	0	0	0	1	1	0	1
D8	0	1	0	0	0	0	1
D9	1	0	0	0	0	0	0
D10	0	1	0	0	0	0	0
D11	0	0	1	0	0	0	0
D12	0	0	0	1	0	1	1
D13	0	0	1	1	0	1	1
D14	0	0	0	1	0	1	1
D15	1	0	0	0	0	0	0
D16	0	1	0	0	0	0	0
D17	0	0	1	0	0	0	1
D18	0	0	1	0	0	0	0
D19	0	0	0	0	1	0	0
D20	0	0	0	1	0	1	1
Times of measurement	4	5	4	6	3	4	9

#### Rating the Translation Responses

Five experts were involved in rating students’ translation responses using the diagnostic checklist. For practical reasons, the division of work was as follows: for 458 translation responses, one expert (rater 1) rated all the 20 descriptors while each of the other four experts was responsible for one translation task (i.e., rater 2 for D1–D3, rater 3 for D4–D8, rater 4 for D9–D14, rater 5 for D15–D20), in this way, each descriptor was rated by two experts independently. The binary rating method was used, that is, “1” was coded if the rater thought that a student’s response generally met the criteria of the descriptor; otherwise, “0” was coded (Kim, 2010, 2011; Xie, 2017; Effatpanah et al., 2019; He et al., 2021). Prior to the rating, the researchers explained to raters specific details in each descriptor. After ensuring each rater has fully understood the coding process and their own responsibilities, we then carried out a pilot rating, the results of which were fairly satisfactory given the high inter-rater agreement. Then five raters began their independent coding on 458 responses, the results of which were acceptable in that the percent exact agreement on each descriptor ranged from 95.63 to 100%. As for the inconsistent part, discussions were conducted between experts to determine the final results. The duration of each discussion averaged about 50 min. After a long process of training, rating and discussion, a final table of 458 candidates’ 1/0 scores on the 20 descriptors was developed to serve as the response data for this study. The examinees’ total scores in the checklist (one point for each descriptor) generally followed a normal distribution (skewness = −0.31, kurtosis = 0.33) with a mean score of 11.84 (out of a total score of 20) and a standard deviation of 2.67.

#### Selecting the Cognitive Diagnostic Models

To select the appropriate CDM with the best diagnostic performance, model fit statistics (absolute and relative model fit) need to be evaluated and compared. According to Chen et al. (2013), the absolute model fit is examined through the evaluation of the transformed correlation (r) and log-odds ratio (l) with their adjusted *p*-values compared to the suggested threshold of 0.05. If the *p*-value of r and l are higher than 0.05, the data is shown to fit certain CDM. As for the relative fit, it aims to help select the optimal model by evaluating indices including Akaike’s Information Criterion (AIC), Bayesian Information Criterion (BIC) and -2 log-likelihood (-2LL). “For each of these three statistics, the fitted model with the smallest value is selected among the set of competing models” (Chen et al., 2013, p. 127). However, as -2LL was shown to always select the saturated model such as G-DINA (Lei and Li, 2014), only AIC and BIC were taken into account in this study. Four CDMs (i.e., G-DINA, RRUM, ACDM, and LLM), the most widely-adopted models in diagnosing language skills, were evaluated and compared in this study by using the G-DINA package version 2.8.7 (Ma and de la Torre, 2020) in RStudio (Version 1.4.1717).

Table 6 presents the information of model fit statistics. As demonstrated, all the four CDMs could fit the response data well as the adjusted *p*-value were all higher than 0.05. In terms of relative model fit, LLM and G-DINA registered the smallest and largest value in AIC and BIC, respectively, meaning that LLM was the optimal CDM while G-DINA was the poorest one in terms of their diagnostic performance among the four CDMs. One thing worth attention was that the AIC and BIC values between ADCM and LLM were very close with a slight difference of 2 (AIC: 10023.99 vs. 10022.58; BIC: 10775.08 vs. 10773.67), which made it difficult to distinguish between the two models in this study (Burnham and Anderson, 2004). In this case, classification accuracy (*Pa*), the indicator of reliability in GDINA package, was employed to compare the two models. Table 7 shows classification accuracy at the test level (*P_a*) and the attribute level (*P_a* A1, A2, A3, A4, A5, A6, A7). Evidently, *Pa* indices of LLM were all higher than those of ACDM, indicating that LLM processed a higher probability of accurately classifying examinees into their true latent classes. Therefore, we decided to select LLM to generate diagnostic information about students’ translation competence in this study.

**TABLE 6:** Model fit statistics.

	G-DINA	RRUM	ACDM	LLM
Adj.*p*-value (*r*)	0.0967	0.2903	0.0616	0.2750
Adj.*p*-value (l)	0.1607	0.8534	0.6026	0.4156
AIC	10060.1	10038.03	10023.99	10022.58
BIC	10939.12	10789.12	10775.08	10773.67

**TABLE 7:** Classification accuracy *Pa*.

Classification accuracy	G-DINA	RRUM	ACDM	LLM
*P_a*	0.5962	0.7181	0.6441	0.6929
*P_a* A1	0.8977	0.9013	0.9143	0.9219
*P_a* A2	0.8976	0.8933	0.8927	0.9085
*P_a* A3	0.9948	0.8901	0.8281	0.8518
*P_a* A4	0.8005	0.8457	0.8426	0.8677
*P_a* A5	0.9999	0.9998	0.9998	0.9999
*P_a* A6	0.8040	0.9988	0.9546	0.9997
*P_a* A7	0.8358	0.9101	0.8905	0.9420

## Results

### RQ1: To What Extent Can Cognitive Diagnostic Models Fit the Data and Then Generate Fine-Grained Information About Examinees’ Translation Strengths and Weaknesses?

As explained above, all the four widely-used CDMs could fit the data well while LLM turned out to be the best one. According to its diagnostic results, students’ translation strengths and weaknesses could be identified for the overall group as well as for individuals.

**FIGURE 1:**
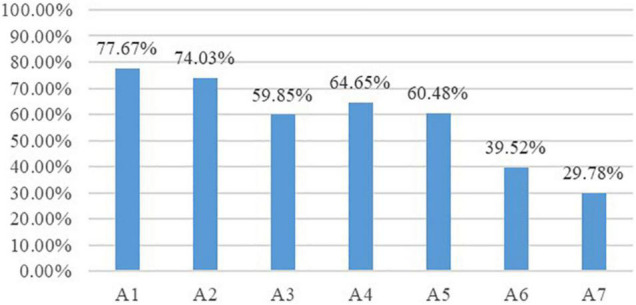
Attribute prevalence of the examinee group.

On one hand, we could discover the overall mastery profile of students’ translation competence through “attribute prevalence” (see Figure 1). As illustrated, students had the highest mastery probability of conveying key information (77.67%) and conveying details with accurate wording (74.03%) while showing the lowest mastery proportion in optimizing logical structures (39.52%) and using the strategy of execution (29.78%), suggesting that A1 and A2 were the easiest attributes while A6 and A7 were the most difficult ones for the examinee group. The three remaining attributes, namely, conforming to language norms (59.85%), conforming to language habits (64.65%), reproducing styles (60.48%), were of similar difficulty for the examinee group in that students’ mastery probability of the three was fairly close.

**FIGURE 2:**
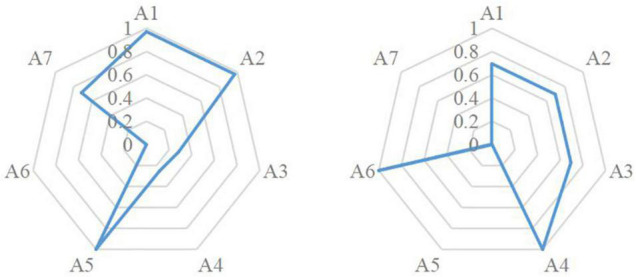
Person parameter estimation specifications of students No.15 and No.26.

On the other hand, we could know each individual’s strengths and weaknesses through “person parameter estimation specifications” generated by LLM. For example, the two radar maps in Figure 2 depicted two students’ (student No. 15 on the left and student No. 26 on the right) mastery information on each attribute, both students scored 13 in the diagnostic checklist, but their weaknesses and strengths were quite different. For student No. 15, A1, A2, and A5 were the strongest attributes while A6 was the weakest one. However, for student No. 26, A4, and A6 were the strongest attributes while A7 was the weakest one.

### RQ2: To What Extent Can Diagnostic Results Help Discover the Relationships Among Translation Competence Attributes?

To discover the relationships among translation competence attributes, correlation analysis was first conducted based on examinees’ person parameter estimations on each attribute. According to Table 8, it was observed that A1, A2, A3, A4, and A5 were significantly correlated with each other (*p* < 0.01), in particular, the correlation coefficients between A1, A2, A3, and A4 ranged from 0.408 to 0.847, meaning that there existed moderate to strong positive relationships among these four attributes (Dancey and Reidy, 2017).

**TABLE 8:** Pearson correlations among translation competence attributes.

	A1	A2	A3	A4	A5	A6	A7
A1	1	0.847**	0.677**	0.420**	0.309**	0.156**	0.046
A2		1	0.662**	0.414**	0.214**	0.043	0.031
A3			1	0.408**	0.280**	0.131**	−0.196**
A4				1	0.200**	−0.007	−0.107*
A5					1	0.071	−0.094*
A6						1	0.028
A7							1

*p < 0.05, **p < 0.01.

According to Chen and Chen (2016a), relationships among attributes could be further revealed by analyzing the co-occurrence of the attributes in dominant latent classes which represented different skill mastery/non-mastery profiles. Table 9 displayed the ten most dominant latent classes and their posterior probabilities. For each latent class, it consisted of seven numbers (“1” means mastery while “0” means non-mastery), the number from the left to the right represented A1, A2, A3, A4, A5, A6, and A7, respectively. Apparently, “1111100” was the most common latent class among the examinee group, indicating that 16.93% of the examinees have generally mastered the first five attributes while failing to master A6 and A7, which concurs with the results of attribute prevalence. Another two major ones were “1111110” and “1111000” with a proportion of 9.64 and 6.40%, respectively. High proportions of the three latent classes signified frequent co-occurrence of the first four, five and six attributes. By adding up the posterior probabilities of their co-occurrence in the ten dominant latent classes (58.7% in total), a proportion of 39.85, 29.91, and 9.64% was found, respectively, suggesting that the most frequent co-occurrence existed among the first four attributes. Such a finding, together with the correlation results demonstrated in Table 8, could reasonably lead to the conclusion that A1, A2, A3, and A4 could form an attribute cluster. Considering the relatively lower frequencies of co-occurrence among the first five and six attributes, it would be more appropriate to infer that A5 bore some relationship with the “A1–A4” attribute cluster, and A6, together with A5 could also interact with the “A1–A4” attribute cluster. Among the remaining latent classes, similar relationships could also be discovered, for example, the classes “1111101” (3.34%) and “1111011” (3.54%) indicated that A7 was more likely to connect itself with the “A1–A4” attribute cluster when it was accompanied by A5 or A6.

**TABLE 9:** Ten dominant latent classes and posterior probabilities.

Latent class	Posterior probability	Latent class	Posterior probability
1111100	16.93%	1111011	3.54%
1111110	9.64%	1111101	3.34%
1111000	6.40%	1110100	3.22%
0000000	5.47%	1100101	3.16%
1110110	3.97%	1101111	3.03%

**FIGURE 3:**
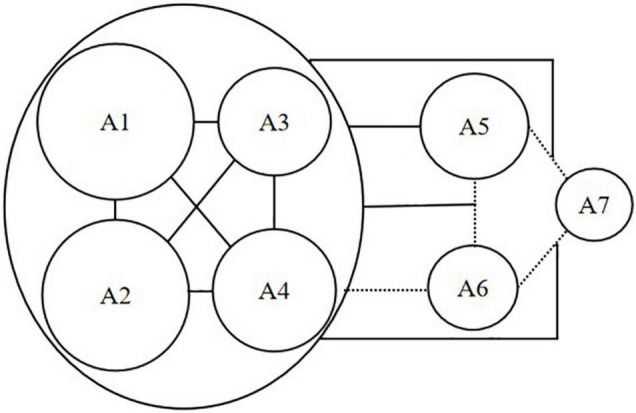
Relationship structure of translation competence attributes.

By taking the findings above into account, the relationships among translation competence attributes could be described by means of the structure in Figure 3. In this figure, the oval box referred to the attribute cluster while round boxes stood for attributes with their sizes denoting the attribute prevalence among the examinee group. In addition, real lines represented certain noticeable associations while dotted lines indicated that no obvious relationships were observed based on correlation analysis and co-occurrence analysis. Such a structure can provide insights into the sequence of skill training (see the Section “Discussion”).

### RQ3: To What Extent Do Diagnostic Results Differ Across High and Low Translation Proficiency Groups?

Given that “a diagnostically well-constructed model was assumed to produce skill profiles that had distinctively different characteristics across different proficiency levels” (Kim, 2011, p. 528), we chose to compare high and low proficiency groups with regard to their attribute prevalence and latent classes.

**FIGURE 4:**
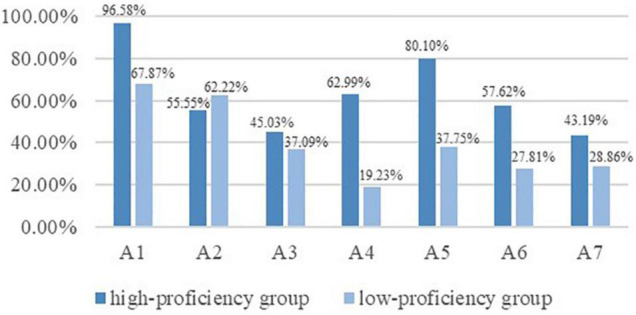
Attribute prevalence of high and low proficiency groups.

According to the scores in the diagnostic checklist, the top 33% (*N* = 151) and bottom 33% (*N* = 151) of the examinees were divided into high proficiency group (*M* = 14.61, *SD* = 1.35) and low-proficiency group (*M* = 8.86, *SD* = 1.65) (Bachman, 2004). Independent-samples *t*-test confirmed that the translation proficiency of the two groups was significantly different [*t*(300) = −33.13, *p* < 0.001]. The attribute prevalence of the two groups was presented in Figure 4. For the low proficiency group, A1 (67.87%) and A2 (62.22%) were the easiest attributes while A4 (19.23%) and A6 (27.81%) were the most challenging ones. By contrast, for the high proficiency group, A1 (96.58%) and A5 (80.10%) were the easiest attributes while A3 (45.03%) and A7 (43.19%) were the most difficult ones. Apart from the differences manifested in their strengths and weaknesses, it could also be found that the mastery proportions of all but one attribute (A2) in the high proficiency group were higher than those in the low proficiency group. For attributes like A4 and A5, the most obvious distinctions across the two groups were observed with an absolute difference of 43.76 and 42.35%. The findings above indicated that the diagnostic results could generally distinguish high and low translation proficiency groups.

Further evidence can be collected by analyzing the common latent classes of the two groups. As displayed in Table 10, among the ten dominant latent classes of the two groups, nine classes of the low proficiency group included less than four attributes (except for “1110100”), indicating that the majority of the examinees in this group mastered less than four attributes. By way of contrast, all the ten classes at high proficiency level included at least four attributes, meaning that most, if not all examinees in the high proficiency group mastered at least 4 attributes. The results above confirmed that the diagnostic results could well distinguish different proficiency groups.

**TABLE 10:** Ten dominant latent classes and posterior probabilities at low and high proficiency levels.

Low proficiency level		High proficiency level	
Latent class	Posterior probability	Latent class	Posterior probability
1100000	6.47%	1101110	9.16%
1010000	5.30%	1111100	7.81%
1110100	5.00%	1101100	7.59%
1110000	4.76%	1010110	5.94%
0100001	4.73%	1111110	5.70%
1100100	4.62%	1000111	5.61%
0110000	4.51%	1110100	4.68%
0000000	4.27%	1001110	4.10%
1100010	4.04%	1011011	3.67%
1000100	3.89%	1001011	3.57%

## Discussion

In this study, we have explored the feasibility of assessing students’ translation competence by integrating CSE with CDA approaches. It was found that LLM was best fitted to the translation responses data among the four widely-used CDMs and could help identify students’ translation strengths and weaknesses at both group and individual level. Moreover, it was discovered that diagnostic results could help reveal the relationships among translation competence attributes and could also differ across high and low translation proficiency groups. Those findings can lend support to the feasibility of such an approach in assessing students’ translation competence, in addition, they can provide implications for model selection, translation teaching and learning, etc.

Firstly, the satisfying absolute model fit statistics of all the four CDMs can enhance the credibility of model selection results in previous studies (Kim, 2010, 2011; Chen and Chen, 2016b; Ravand, 2016; Xie, 2017; Ravand and Robitzsch, 2018; Effatpanah et al., 2019; Javidanmehr and Sarab, 2019; He et al., 2021). The selection of LLM in our study is consistent with that in He et al.’s study (2021), which may be attributed to the fact that both their study and ours were conducted under CSE descriptive framework and both studies dealt with data obtained from constructed-response tests. Apart from LLM, both ACDM and RRUM turned out to fit the response data better than the G-DINA model in our study, which shares certain similarities with some writing diagnostic studies (Kim, 2010, 2011; Xie, 2017; Effatpanah et al., 2019) in their preference for the former two CDMs. Another advantage of LLM, ACDM, and RRUM over G-DINA model in our study was the classification accuracy, which could be accounted for by the fact that only 458 examinees were included in our study while a large sample size was required by G-DINA model in order to generate accurate estimates (de la Torre and Lee, 2013). Given this, it can be predicted that the G-DINA model may be more suitable to be applied to large-scale selected-response tests while data from constructed-response tests with a small sample size may function better under other models like LLM, ACDM and RRUM. Such a prediction can provide references for model selection in future studies.

Secondly, by knowing students’ strengths and weaknesses at the group level, teachers can carry out remedial teaching by adjusting their teaching design (content, method, etc.). In this study, A6 (optimizing logical structures) and A7 (using the strategy of execution) were shown to be the most challenging attributes for the examinees, so teachers should attach importance to these two subskills in their teaching. Also, by presenting different mastery profiles of two students with the same score, the study clearly illustrated the limitations of the traditional way of assessment under the CTT theory and highlighted the advantage of CDA in discovering in-depth information under the same score. Such fine-grained information can offer each student with personalized feedback, which then, helps promote their individualized learning.

Thirdly, person parameter estimations and dominant latent classes helped uncover the relationships among translation competence attributes. In this study, conveying key information (A1), conveying details with accurate wording (A2), conforming to language norms (A3), conforming to language habits (A4) were found to form an attribute cluster. As those four subskills are all closely related to bilingual knowledge and competence, such a cluster can be named “linguistic attribute cluster.” The formation of the linguistic attribute cluster is conducive to teaching by informing teachers that these four attributes can be taught concurrently. Given that A6 and A7 could only interact with the linguistic attribute cluster when accompanies by A5, they are not suitable to be taught prior to A5. In a similar vein, it is not advisable to teach A7 earlier than A6 considering the former was connected with the linguistic attribute cluster only when it occurred along with A6. As analyzed above, it can be seen that the relationships structure of translation competence attributes was established with the linguistic attribute cluster as its basis in that the last three attributes were either individually or collectively connected with the cluster, which, to some degree, echoes Yang (2002)’s statement that “linguistic competence forms the major constituent element as well as the foundation of translation competence and the strengthening of the latter has to rely on the enhancement of the former” (p. 16). As the examinees are novice translation learners, the central role of linguistic attribute cluster also lends evidence to Yang (2014) experimental results that the effects of Chinese learners’ language proficiency on their translation proficiency were particularly strong in the initial stage of learning translation. In addition, the finding that A7 could be associated with the attribute cluster only when working with A5 or A6 corroborates the proposition that “translation strategy is, in essence, a problem-solving means used to achieve certain goals” (Bai et al., 2018, p. 104). For the examinee group in this study, translation strategy was used mostly for reproducing style (A5) or optimizing logical structures (A6).

Fourthly, the result that the high proficiency group mastered more attributes than the low proficiency one is consistent with that in Du and Ma’s study (2021). However, different from previous studies in which high-proficiency levels perform better in all attributes than low-proficiency ones (Kim, 2010, 2011; Xie, 2017; Fan and Yan, 2020; He et al., 2021), there existed one exceptional attribute in our study. Such an exception can be explained by the limitation of the sample in this study, that is, the examinee group is generally homogeneous in their translation learning experience and thus in their overall translation proficiency. Since they are all novice translation learners who had just learned Chinese/English translation systematically for half a year, it is likely that there is no obvious distinction in their mastery of certain translation subskill(s) even if their total scores are significantly different. In this case, it is assumed that such an exception may no longer exist if more experienced examinees (e.g., advanced translation majors) are involved.

## Limitations and Suggestions for Future Research

Notwithstanding instructive findings and implications, several limitations exist. Firstly, constrained by the test format, attributes related to translation knowledge and the strategies of planning and appraising were not assessed. Secondly, in order to standardize the testing procedures and thus guarantee the quality of response data, only 458 examinees from the same school were involved. In the future, it is suggested techniques like Translog, screen-recording can be used to assess such subskills as using the strategy of planning, appraising and compensation. In addition, the sample size can be enlarged with translation major students involved, in this way, the relationships structure of translation competence attributes of non-translation majors and translation majors can be compared so as to further investigate the difference between those two groups and then provide insights for translation teaching targeted at the two different groups.

## Data Availability Statement

The original contributions presented in the study are included in the article/Supplementary Material, further inquiries can be directed to the corresponding author.

## Ethics Statement

Ethical review and approval was not required for the study on human participants in accordance with the local legislation and institutional requirements. Written informed consent from the patients/participants legal guardian/next of kin was not required to participate in this study in accordance with the national legislation and the institutional requirements.

## Author Contributions

HM: design of the study, data collection and analysis, and writing original draft. HC: data collection and analysis, revising the draft, and supervision. Both authors contributed to the article and approved the submitted version.

## Conflict of Interest

The authors declare that the research was conducted in the absence of any commercial or financial relationships that could be construed as a potential conflict of interest.

## Publisher’s Note

All claims expressed in this article are solely those of the authors and do not necessarily represent those of their affiliated organizations, or those of the publisher, the editors and the reviewers. Any product that may be evaluated in this article, or claim that may be made by its manufacturer, is not guaranteed or endorsed by the publisher.
