# Supplementary material for: Assessing Students’ Translation Competence: Integrating China’s Standards of English With Cognitive Diagnostic Assessment Approaches
Source: Front Psychol. 2022 Mar 31;13:872025. doi: 10.3389/fpsyg.2022.872025 (PMC9008137; doi:10.3389/fpsyg.2022.872025)
Supplement: Supplementary file 1 [file Data_Sheet_1.DOCX]

Supplementary Material (translation tasks)

**English-Chinese translation**

1. We believe that global warming is a serious threat, and that the world needs to take steps to try to avert it. But we do not believe that climate change is a certainty. There are no certainties in science. Prevailing theories must be constantly tested against evidence, and more evidence collected, and the theories tested again.
2. It has been noted with concern that the stock of books in the library has been declining alarmingly. Students are asked to remind themselves of the rules for the borrowing and return of books, and to bear in mind the needs of other students. Penalties for overdue books in the future will be strictly enforced.

**Chinese-English translation**

1. 裸婚是一种新的结婚方式，指一对恋人没房、没车、不办婚礼、不度蜜月，只领取结婚证的结婚方式。现代年轻人的生活压力大，且强调爱情的独立，必须大肆操办婚事的传统在年轻一代中逐渐削弱了。
2. 首先引起安德鲁注意的是一架波音767型飞机。这架飞机从波士顿飞往洛杉矶，载着81名乘客。飞机上午7:59起飞，一直向西飞行，飞过阿迪朗达克山（the Adirondacks）上空，突然调头南下，冲向纽约市中心。
