# Supplementary material for: Assessing Students’ Translation Competence: Integrating China’s Standards of English With Cognitive Diagnostic Assessment Approaches
Source: Front Psychol. 2022 Mar 31;13:872025. doi: 10.3389/fpsyg.2022.872025 (PMC9008137; doi:10.3389/fpsyg.2022.872025)
Supplement: Supplementary file 2 [file Data_Sheet_2.DOCX]

Supplementary Material

***Transcript of students’ verbal reports***

Texts that help identify different attributes are presented with different colors: conveying key information; conveying details with accurate wording; conforming to language norms; conforming to language habits; reproducing styles; optimizing logical structures; using the strategy of execution

S1：

英译汉我主要是采取直译。从第二句开始，我觉得在英文中第二句和第三句是分开的两个句子，我认为它是有一定的逻辑关系的，因为我们相信气候，但是我们相信气候变化不是必然的，是因为科学没有必然性，所以我在这里增了一个连接词。然后最后一句话主要是把英文的一个被动语态翻译成汉语的时候，是变为一个主动语态。然后第二句也主要是直译，唯一的变化就是最后一句英文翻译过来，直译的话就是惩罚，就对于过去逾期未还的书的惩罚，其实将会被严厉的执行。然后这里也是涉及到英语的被动，然后翻译成一个主动。然后用的是那个overdue books 英文中是一个逾期未还的书，我在想这里是不是涉及到一个移就的修辞，然后我就把这个书换成那个人，就是对于逾期未还者严格执行惩罚制度。然后接下来是汉译英第三句，然后第一句话就是裸婚是一种新的结婚方式，然后指什么什么。其实这句话的主语就是裸婚，最开始我想的是把它全部就译出来，Have no house, no car，后来发现太长了，然后最后我就还是把主语提出来，然后中间设了一个插入语，然后refers to，最后就是用了一个result，虽然不办婚礼，不度蜜月在中文也是一个动词的表达，但是我还是把它与前面的没房没车用一个without 全部连接起来。然后只领取结婚证的这个表达在中文里是一个动词，是一个主谓结构，然后我在英文中把它翻成了一个偏正结构，就是registered couple，然后做了一个这样的改变。然后最后一个就是现代年轻人生活压力大，然后就也是主要他们是面临了很大的生活压力，所以我增了一个动词face。然后强调这个爱情的独立是直译的，然后削弱是做了一个伴随状语把它译出来，然后必须大肆操办婚事的传统。我觉得我把它概括化了，就是一个extravagant marriage，是一个奢侈的婚姻。然后在他们新的一代就是被削弱了这样的传统，然后就也是简单做了一个概括化的处理。然后最后一句可能对我来讲，我觉得有点难。然后同样的最后一句第一句就也是把主语提出来了，然后是这个波音767，然后吸引了安德鲁的注意然后从最后把这架飞机从波士顿飞往洛杉矶，还有载着81名乘客。我就把它翻译成英文的时候，我就直接也是做了一个插入语，插在这个主语的后面，然后对它进行一个说明。最后就是飞机上午七点钟起飞，然后下去飞行，飞过了什么掉头还是冲向，我觉得这种短句是我一直不好处理的，因为它是一个动词，然后就像那种说“我睡下了，然后我也醒了，然后去上了个厕所，然后我又睡下了”这种类似的动词，我觉得不好处理。但是我还是把它提出来做前面做了一个状语，然后taking off，然后就是那个然后用it ，把它后面的再顺下去了，但我觉得处理的不是很好。

S2：

我是先把第一句这一整段话给读了一遍，读了一遍之后，我发现第一句和第二句它中间存在一个转折的关系，但是我们中文里面都是会把那个转折词给说出来的，比如虽然但是什么的，所以他第一句话前面我加了一个，虽然我们相信这个全球变暖是一个巨大的威胁，这个地方serious 本来是一个严重，但是我觉得严重的威胁是不怎么......然后后面这个the world，我觉得the world是指的是世界各国，如果单纯的只是说全世界的话，可能那个主语就不太对，我觉得是世界各国。那我这个地方选择了增译，增译成了世界各国也需要采取措施去避免它的发生。然后这个avert是避免的、免除的意思，然后我再把它翻译成了避免它的发生，然后但是承接下面的虽然，但是我们不相信它的气候变暖是绝对的，科学界里也没有那种绝对的事情。certainty 本来是个名词，但是我在这里把它变成形容词。后面这个理论普遍的理论，流行的理论，理论必须是不断被检验的。然后是通过这些证据来不断检验的，然后更多的证据这个地方是没有“当然的”，但是我把这个and就是单独拎出来，就是把它译成“当然的”做一个承上启下的连接的关系。然后随着越来越多的证据被收集，然后理论也需要被重新检验，这就是我的第一段话。然后第二句话，其实第二句话是it 这个结构这个地方我当时还是纠结了很久的，就是这个it has been noted with concern，我当时讲的是人们关切的注意到，然后我就觉得这个很怪，或者是人们带着一些担忧...十分的担忧注意到，然后我就觉得很怪，我就把它翻译成了人们密切地注意到。然后这个stock储存量，就是图书馆的储存量在下降，这个alarming 我直接翻译成以惊人的速度在下降。后面是学生被问起，当他们要求会去被那些图书馆的借书或还书规则。**后面这个句子就是我比较那个疑惑的地方，就是这个bear in mind the needs of other students，这个地方我就不知道这个句子跟这一种的话有什么关系，我觉得他无论是藏书量还是借书，还是对以后的那个惩罚来说，其他学生的需求我觉得他是没有任何关系的，所以我就只能把它直译了，当时我也想了，最后就把他直译的这种单独放在这儿，我感觉肯定是有问题的。**后面的是今后，我把那个in the future 给提前了，时间状语我直接拎到最前面，然后我就说对书籍逾期不还的这个地方，我也是选择的增译。他本来想说的是，逾期不还的这种现象，但是我觉得对现象进行惩罚的话，我觉得不太对，然后我就把它变成了一个对逾期不还的那个书籍逾期不还的学生进行严格的惩罚。然后第三句话第一长句就是前面的很长的一些并列的结构，我就单独的把它变成那个独立主格，就是直接把它变成了一个介词加名词，并列名词，然后后面是把他们提前了之后，再裸婚是一种新的结婚方式，把它放在后面，然后后面有个定语从句叫做裸婚，然后后面是现代年轻人压力没有那么大，然后我就直接把它给切了。对于生活，现在年轻人生活压力大，我直接把它切。对于现在年轻人来说，然后我在modern young，然后they live with the great pressure，然后用并且更加注重他们的爱情的独立性。后面我是用了一个so that 这个结果状语从句，然后就直接后面接着他们。因为是我觉得是上面有因果关系，因为他们觉得压力大，并且爱情的独立，然后我所以才会导致他们会对那个大肆操办婚事的传统意识给削减了，所以我这个地方用的是so that，把它后面承接起来，主要是承接。然后那个“因为”是我把最后一句接在上面一句话，所以他后面在操办婚事的传统在年轻一代中，然后我就直接把这个年轻一代给省了，因为我觉得我前面有了对于年轻一代来说，然后for the modern young，我就直接把这个地方给丢掉了。然后就说在对他们来说，这个东西已经削减了。然后是第四句话，第四句话就是第一个首先吸引这个安德鲁的是波音747，然后后面这个句子我就直接用it 直接代指前面的那个波音747的飞机，然后我先说，他载着81名乘客，然后再去说他是被从波士顿飞往洛杉矶的，然后我用一个非谓语。从7点59开始起飞，然后说他是向西一直向西飞的，然后后面也是一个时间状语，然后说当他飞过这个阿朗迪的上空中，我用一个when来引导，然后说他飞过这个山的上空的时候，然后他再说主句，就是他突然向南边儿，然后后面还是一个ing的一个非谓语结构。然后就说他突然冲向了一个纽约的市中心。

S3：

首先第一句其实英语里面有很多这样的句子，就是we believe that，我倾向于把这个句子重要的地方放在前面说，他这个地方就是在讲一开始这个句子就讲的全球变暖，所以我把全球变暖放到前面了。它是个大隐患，然后后面就是这个that 全世界都需要一起行动起来，然后来消除这个隐患，我就避免把前面翻成我们认为这个地方我就去掉了。然后后面那个but 那个句子is... don't believe 这个地方否定转移的话，我们应该放在后面，所以我就很自然的把它翻成了这个非必然的事件。为了它对应的更加的清晰一些，我后面加了事件，并没有翻成不是必然的感觉，它这个句子节奏可能会好一点。还有就是后面那个科学界没有什么事情是绝对的，这个地方，我也是加了没有什么事情，加了这个事情的话，也是考虑到这个句子结构问题。然后后面我觉得这个我可能属于是一个误译，因为这个地方当时想的时候，这个prevailing它就是那种比较普遍那种，就是传播的比较广一点，比较famous的那种，但是我觉得它也不一定就是常用的。然后他说不断的要constantly tested against evidence，不断的用证据来检验这个地方，我觉得证据这里就怎么感觉不太地道，然后后面的我也是用的这些理论就能......收集的证据越多，这个地方我也是用的证据，但我总觉得这个证据在这里有点怪怪的。第二句话，我也是一样的，我也是it has been noted，那个地方也是有什么人们指出，这样的意思我也没有翻，我直接从后面开始that这个后面的从句。藏书急剧的减少，它是再回到前面的这个with concern，就感觉这是一件让人担忧的事情。然后后面我是看到那个主体，首先是这个学生，他们就是要求去还书，要遵守那种规则，借书跟还书，然后要牢记还要考虑到其他同学的借书需求，后面那个的主语就变成了处罚penalty，所以我就想前面是学生为主体，后面我就想再用一个什么主体，但是没有找到，我就加了一个相关人员来进行这个处罚。第三句汉译英，其实我觉得比英译汉肯定要难，因为毕竟也不太熟悉这个地方。我一看这个句子，就想把前面那个是一种新的结婚方式，用as来引导，然后指没有什么没有房车、婚礼、蜜月，有结婚证的这样一种结婚方式。因为前面有一个结婚方式，然后后面有一个结婚方式，我们肯定不能就真的翻地那么的重复，所以我前面用as之后，后面就是有什么。然后第二句话，现代年轻人的生活压力大，我就很自然的想到用那个live来翻译他们过的一种有压力的生活，就用and 把那个强调联系起来之后，我就觉得那个前面不是stressful，后面有个stress，感觉起来应该可以换个词可能会更好，然后必须大肆操办，或者这种传统被削弱了，就是因为我前面，压力大了，然后也强调爱情的独立他就被削弱了，所以我就很自然想到用which引导了一个从句。第四句话，说实话这个句子我觉得是这三个句子里面最难的一个，主要是觉得这个句子比较散，也有想过整合，但是在整的过程当中会出现一些问题，我怕整错了，所以有一些我不敢整在一起的，还是没有去整在一起。第一个句子它其实就只是引出来了这个波音747型的飞机，所以我就把那个相当于第一个句子和第二个后面这架飞机从哪来的，飞到哪儿去的，然后有多少名乘客，这个句子我就还是把它大胆地整在一起了。然后从飞机上午几点起飞，一直飞、向西飞、飞过了什么地方。这个地方我觉得存在一起也是没有问题，我就把它整在一起了。它飞过，我觉得它应该是一种伴随着，因为他一直向西飞，这个过程当中他飞过了，所以我用ing形式，突然掉头南下，我觉得它就有一种转折的意味，所以我就把它又另起了一个句子，用then 来引导的。掉头南下之后就直接冲到，然后他这个里面的动作衔接地也是很紧密，它南下掉头了之后，就冲向了纽约市中心。所以我就也用了ing形式的，相当于他们两个动作之间发生的比较近。

S4：

好的，首先就是我看到整个句子它不是有两个believe，第一个是表示肯定的，第二个是表示否定的。然后我就从逻辑方面，我就当时以为就觉得他是一个对比，所以我就有了一个我们相信但是又不相信，然后这是我一个点。然后再就是到后面，我觉得比较不好确认的地方就是一个还有一个certainty，它说气候变化是一个事实，然后我就觉得他就是说他既然之前已经说了，全球变暖是一个威胁，然后后面又说了一个not believe，我就觉得我理解的就是他们就是不相信这个气候变化，它是已经成为了一个事实的。然后我就是翻译成了气候变化已然来临，然后再加上最后一句，其实我当时翻的时候，我不是很理解这个逻辑，就是在tested against evidence 那里。然后我觉得我就理解的是，就是要我们用这个证据是用来检验这个理论的。然后证据越多，然后这个理论就被又证实了。但是我当时翻完之后，我就想，我说既然他第一句话又说全球变暖是一个严重的威胁，我想这个句子之间的我就觉得我翻译的句子之间的逻辑挺奇怪的。然后第二段的话，主要是我当时在想noted with concern，我就想把这两个意思都翻出来，就是人们关注到了这个问题，人们又很担忧，然后我就把它分开放。我说令人担忧的是，人们关注到这些，然后再就是图书馆书籍的贮藏量...我觉得后面的我倒是没有什么问题了，我觉得这一段比上一段要好理解一些。然后第三段的话，我当时想，它说结婚是一种新的结婚方式，然后又解释了这种结婚方式是哪一种结婚方式，然后我就用了一个which 的定语从句，把它联系在一起，我想要让它表达的更紧密一些。然后这里的必须大肆操办婚事的传统，我翻译的是celebration，但是我想的是可能这种celebration 就把那个大肆操办给翻掉了，然后我也不知道怎么把这个意思翻出来。然后第四段的话，我当时想讲的是就是说能够怎么样把他这个句子变得更紧密一些，因为我觉得他中文写的，他其实就是句子都很短，就是一个一个的短句，然后我就想把它联系的更紧密一些，然后我就用了一些介词，然后把它给加进去了，但是我不知道我加的这样是合适的还是不合适的，这就是我当时的想法。

S5：

首先就是这个第一句就是他开头就是一个we believe还有后面有一个对应的，we believe not，在这里我觉得这个不应该说我们相信什么的，我觉得这样就会非常的过于生硬，于是我把他提前，然后就说在我们看来。**然后后面就是we need to avert it, 就是这里it 是一个非常有指代性的一个词，然后我就想把它给它具体一下，就具体化成全球气候的这个问题**。然后后面we don't believe这里我就没有再重复这一点，然后就这一点就差不多就是省略的这样一个。然后就是在我自己在做的时候，我觉得最后一句是有困难的，因为最后一句有一种很反复的感觉，但是中文里面这种又不能词语过于反复，所以我就把这里的许多词语都给它有一个替换的这样一个过程。然后第二句，对于开头这个最开始我是有想很久，因为这个因为如果译出来的话，就是我对于这个noted 和concern 之间，这两个我总会下意识的就丢失一个意思，然后我就干脆把它分成了两个句子给它分开，给它拆开...这样一个解决的这个方法。然后还有就是后面的这个，这个主语是学生，但是这个地方我觉得这个主语我就给他稍微替换了一下，我就把注意替换成了图书馆，图书馆要求学生怎么样做，而不是学生被要求怎么样做，这样可以更直接一点，而且和整个句子的主语会更搭配一点，因为它整个都是在说图书馆里，图书馆对人们的一个要求，然后我就把它这里换成了图书馆。还有就是他后面的这些我都相应的有做出一些调整。然后第三个，这里最开始是这个裸婚我有找一下，然后找出来比较倾向的还是个naked marriage。然后我就选择这样一个词，然后后面这个没房没车，不办婚礼，不度蜜月，这里最开始我的处理方式是有一点过于冗长的。我觉得我现在这个也是有一点过于冗长的，但是他这里我觉得就是没房没车，我觉得还是要指代清楚一下。因为它不是指真的没有房子，没有车子，而是他们不选择去买房，不选择去买车，然后我就把这里这个联系给它显示出来了。然后还有对于这个爱情的独立这一点，就是我自己在做的时候，我不是很明白爱情的独立是一个什么意思，就是他独立指的是哪个方面，而且这里因为我没有太懂，所以我就没有把这个过于具体化这么一个表述出来，我就只能很概括性的，我就直接把它这么译出来了。然后这个大肆操办的，这里我觉得就是不再像从前那样传统的那样花很多的钱，很多的精力去办这么一件事情，然后我就在这里添加了一个traditional这个词，然后特别是这个句式的时候，这个句式这里写的是必须大操大办，这里最开始我想的是用一个must，但是我觉得如果用must 后面的东西太多了，然后最后我就把它改成了一个名词，改成了一个necessity，我觉得这个会更简练点。然后就是最后一句，我最开始看的时候就觉得是一个很多短句组合成的，然后这个句子就需要去给它整合起来，用那种有一点逻辑的那种关联词什么的，我是想着用时间顺序给他联系起来。我自己写的是从时间，然后载的人，然后他从哪里到哪里，这个地方我就给他全部给他连上了一句。然后在后面的时候，就是后面就是说他从哪里飞过哪里，然后掉头怎么样的，然后又冲向怎么样。然后这里我就给它....怎么说呢？就是有一点那种强调的感觉，然后还有这个用词的方面，我也想了一下，最后一句是冲向了纽约市，我觉得这个冲向了纽约市这个要强调一下，然后我就这里这个用词，我就直接用了headed 这个词。掉头南下这个地方，我之前也想了一会儿，因为如果要往里面融的话，就是不太好给它加进这个句子里，然后我就直接把它作为了一个后置的一个定语，给它放在了最后面，这就是我在做的时候这么一个思路。

S6：

首先我看到那个...做了一个词性的转换，就是那个serious threat。之所以把它翻译成了动词，严重威胁。首先是我不知道翻译成名词的话，那个serious 应该翻译成什么和那个名词搭配，**然后也觉得中文比较习惯用动词，所以就把它翻译成了动词**。然后后面需采取一些措施，它原句子是有一个the world 这个主语的，但是我觉得首先措施是有人采取的，所以翻译成世界就不太妥当。但是我也不知道应该用什么主语，因为我觉得一开头这个我们他不是指所有的人，但我觉得如果采取一些措施的话，可能所有人都有一定的责任，所以就采取了一个无主语就没有用主语。然后后面这个伤害，我没有翻译成威胁，是觉得我觉得威胁已经存在了，就感觉已经无法避免了，所以感觉他应该想要避免的是对我们进一步产生实质性的伤害，所以就翻译成了伤害。然后后面的在科学中也没有必然这一说法，根据自己的语感，感觉自己平时也特别喜欢说，也没有什么这一说法，所以就应用在了这里。剩下的第一句这个词当时搜了evidence 的，它其实有证据依据，感觉证据更像是像是法院里面的那种证据，所以感觉用在这里不是很妥当。然后后面那个句子，他原文的意思就是每收到更多的证据的时候，只要再次检验理论的准确性，我感觉如果这样翻的话，就不是特别的顺畅。然后我自己的理解的意思也是就是他每收到新的证据依据的时候，就会去再次检验这理论的准确性。第二段一开始它是noted with concern，开始看到这个我不是怎么知道处理这个句子，本来是觉得就不翻，直接说人们注意到，但是感觉还是需要翻译出来，不然就有点漏译，所以把它放在后面处理。然后后面那个原句子它使用的是一个被动语态，但是我感觉用被动不太妥当，中文也很少用被动。但是因为我当时也不知道该用什么主语在这里，所以就为了上下文的连贯，在这里增加了一个we解决这一问题。然后后面的句子基本上都是跟着直译的。本来最后一句我翻的是日后会针对逾期还书的情况，处以严厉的罚款。但是我感觉这个搭配就像是对情况处以罚款也是有点不太恰当的，应该是对人，所以在这里增加了一个对相关人员处以严厉的罚款。英翻中的基本上就是直译了，然后就是没房没车，不办婚礼，不度蜜月。原本我翻的是without buying house or car or holding a wedding and spending a honeymoon。但是我又觉得没有房，没有车，它是没有，但当时又想但是他原来就有一套房，那就不用买了，但是现在想想可能是我想太多了。然后后面那个的they only get a marriage certificate，我原本是想增译一个to show they have got married，就感觉不翻这个好像又少了点什么，但是翻了又感觉好像原文也没有那个意思，就还是像这样处理的。关于那个爱情的独立性，其实这个句子我当时也不是很理解，然后搜了一下中文，也感觉没有搜到什么东西。直译independence of love其实感觉还是不是很准确的。还有大肆举办婚事，我当时理解的就是感觉是举办一个隆重的婚礼，然后就用了一个hold a grand wedding，然后最后一段基本上也是就是按照他每一个句子字面意思翻的。其实感觉最后一段翻了很多地方都处理的特别不恰当，比如说那个rush，其实我感觉这个意思好像有点翻重了，冲向就想用一个介词toward，但是因为前面有个northward 感觉就有点重复了。

S7：

首先他第一段话的话，我把它看了一遍，我觉得它确实不是很长，所以它我没有调换它的顺序，然后我就这样顺着译下来了。然后那我就这样翻的时候，首先第一句说，我们认为全球变暖是...我开始翻的时候就直接翻成我们认为全球变暖是严重威胁，但是我觉得翻完以后有一点奇怪，就把它换成了人类正在面临的严重威胁。但是我翻这一句话的时候，我觉得这句话就翻出来，有一点就很硬，有点翻译腔，我就有另外两种想法。第一个就是说全球变暖正在严重威胁人类的什么东西，但后来后面的什么东西，我觉得要加一个什么，比如说生活或者是生产的那种，但是加了的话就好像有一点...我又担心说是不是太多了，翻得像一些原文没有的东西，然后就把这个舍弃。然后还有一个想法，就是说我们将全球变暖视为一场严重危险，但是这个又感觉好像把这个重要性减少，最后就还是按最开始的时候，我们认为全球变暖是人类正在面临严重威胁。然后第二句的话，我翻的世界各国应当立即采取措施避免这些问题。世界各国是因为我看到这一段话的时候，我觉得他应该是一个国际间的什么会议上面的，然后可能要呼吁各国干嘛的，就翻译成是世界各国。其实我翻译这个应当的时候，我一开始想的是把它翻成必须强调的这个采取措施的紧急性。但是我觉得但是必须的话，好像一个国家是我们必须干嘛，就好像有一点不太好，那还是翻成应当。立即是我加的一个词，我觉得可能就想表达那个紧急性，采取措施避免这些问题。这些问题我觉得不是很恰当，觉得这个措辞不是很好的，就避免这个问题。然后第二句的话但我们并不认为气球变化气候变化是必然的，科学无定论。后面这个科学无定论是我第一遍看这个译文的时候，我就觉得好像可以这样翻，还挺简洁的。但前面说，像我们并不认为气候变化是必然的，就是按字面意思翻，但是我翻这一句话的时候，我觉得有一点，其实我不太能理解这句话，就是说我们并不认为气候变化是必然的这种变化，因为我觉得他跟我们的常识有一点有点矛盾，我不太确定这样照着翻对不对。因为我觉得这个变化它已经产生了，就是它确实已经发生，然后就不是必然的话，有一点好像不是很对。然后后面我在想要不要说这个变化不是不可避免的，或者说不可改变的，但是最后还是没有改，还是用的是必然的。然后最后一句话说，盛行的理论必须不断接受检验，然后收集证据，再次检验理论。我觉得这一句话，我还是按照字面意思翻的，但是我觉得翻的有一点就是它中间有一个我觉得还有一个主语的偷换，就是一开始说必须接受检验理论，它的主语是理论，然后收集证据，再次接受检验，他们后面两句话的两个分句的主语好像是人，前面一个是理论。我不知道汉语可不可以这样，就是说英语应该是不可以这样。但是我这一整段话翻下来的话，就是我这边中间其实有很多疑问和很多问题。但是最后都没有改，因为我就是按照之前我们课上老师说，就是说能直译就直译，然后不会造成很大的问题的话，尽量按照字面意思翻，然后就这样翻下来了。第二段的话，首先他第一句话，我觉得他就应该要加一个主语，因为它的英文原文主语中用的是it，it has been noted with concern that 就是我觉得他肯定就是你要译成中文的话，加个主语。然后我用了一个大家关注到....，我就用大家的时候，我觉得其实有点奇怪，但是我就用人们关注到好像又不太合适，用学生的话，原文里面又没有很具体的说这个东西虽然我觉得肯定是跟学生说的，但是这里就直接翻成学生的话，好像不太好。最后我就翻成了大家关注到，关注的话，关注是因为这有一个concern。我觉得关注比注意的话好像要更符合一点，程度要更深一点。然后第一句话翻的是大家关注到图书馆的图书急剧减少这个现象，令人担忧。就是用了一个很中文比较死板的那种模板。然后第二句是一个被动句，就下意识把它换成了主动的。就因为是学生们被要求干嘛，然后我就翻成了学生们要干嘛。然后第三句话就是他这个原文，他说罚金也是一个被动句。然后我觉得英文中用被动句的话，中文就是绝大部分都是要把它换成主动的，然后我就把他的主语换成了借书逾期未还者，从那个罚金换成人，然后就我觉得这一段的话，就主要就是一个主语的转换，然后就这样翻了。然后第三段，他后面两段是汉译英，这个第三段有两句话。第一句话就是说裸婚是有什么，然后指什么，然后我就下意识把它翻成它是一个什么，然后用一个非限定性定语从句which......就这种的话，可能一般情况下，我要么就把它分成这样一个主句，加一个which引导的非限制定语从句，或者是用一个as 引导的什么状语从句，然后后面再用一个主句，但是后面因为后面我看到说现代年轻人的生活压力大，我觉得后面也可以译成一个从句加一个主句。然后后面就用了as的那个句式，就把前面换成了非限定性定语从句，然后我觉得他这一句话他没有很多很难的地方，就是一些措辞。裸婚我去查了一下词条，就是naked marriage，我觉得他现在已经有这样一个约定俗成的表达的话，既然他这个我觉得他已经有一个固定的表达，我就用上了。然后我看后面的什么恋人没房没车，就是后面我觉得他就可以就用一个without加一个名词，因为后面房车婚礼蜜月结婚证的话，他就都可以把前面的动词省掉，全部包含在那个result 里面，后面用一个名词就可以了。然后第二个句子就说现在年轻人生活压力大，就强调爱情独立。然后前面这两个小句我就把它翻成了放在as里面的那个从句里面，后面的the tradition...这个就翻成了后面这个句子的主句。这个有一个问题，就是我就用这个逐渐削弱，我用这个削弱这个词，我觉得有点不太对，这个diminished 我觉得好像不是很确切，但是我没有找到合适的词。然后第四句的话，就是我看到这个句子，我觉得他这一整段的话，好像除了第一个，一整段我觉得它的主语都是飞机，但是一开始我想能不能把它们译成一整个句子，就可能我平常翻译就有这种毛病，就想要能翻长一点，就是能翻译一个句子，就翻成一个句子。我觉得太长了就把它翻成两个句子，翻成两个句子的话就肯定要处理一下他们的主句和从句。然后第一句话就是他首先引起他注意的是一个飞机，然后这个飞机后面用一个从句来which引导的从句来修饰这个飞机，就是他从这个波士顿飞到洛杉矶，然后带着81名乘客，然后就从这里转开了。然后第二句说飞机它从什么时候起飞。后面这个短句就是非常多，一开始我想着能不能跟前面一样，也处理成一个从句加一个主句，但是没有理清楚，就直接翻成了并列的句子，就用and，然后还有but把它翻成一个简单句。这句话有一个问题就是我不理解，就是他是飞过阿迪朗达克山，他这个飞过我不知道他说的是刚好飞在那个山上面，还是把那个山已经飞过了。然后我翻的时候就把它当做它已经飞过了，就用了over 。

S8：

首先我当时翻译的时候，这个believe 我就纠结了一下，因为一般情况下这个believe 我们就会直接译为相信，但是我觉得在这个情况下，我们也可以译的比较口语化一点，就是译成了知道，因为它本身也有这个方面的意思。还有另一个比较另一个可能让我纠结了一个就是那个certainty，这是一个确定的事。但是后面也有一个certainties ，我要想这两个要怎么处理呢？因为两个如果重复的话，好像也不太好，气候变化那个气候变化不是一个确定的事儿，好像有点冗杂，然后就直接把它简化成了一个定式。然后后面一句就是为了解释一下，就说是科学中没有什么是一直确定不变的。还有我翻译的时候，我觉得一个难点就是最后一句话，那个被动句要怎么把它很自然而然转换成转换成主动句，而且看不出翻译腔。还有就是the theories tested again，这句话我觉得也挺难处理的，所以我就选了一个最笨的方法，就是直译，所以就直译成理论就会再次被检验。第二段那个存量就是直译，然后学生那句话，学生是被提醒就是被要求，但是我觉得这样生硬的译成被字句太过于生硬，我就转换了一下，转换成学生应时刻提醒他们自己，其实我知道这里是别人提醒学生，别人要求学生。还有就是那个我把原文中的名词短语译成了动词，就是borrowing and return of books 译成了动词。最后一句的话我理解的就是就像我们学校的图书馆，如果你借书，你逾期未归还的话，你下次去还的时候，你就要付那个补偿，就是有点像罚款一样，你就是要给他付钱，所以我就把这里是不是翻译成了罚款。还有它有一个strictly，我就想强调一下，严格严惩，就像那种犯罪了要严惩一样，就是严格处罚，严格罚款。第三个，首先我看到裸婚这个词的时候，我就想真的要翻译成naked marriage吗？然后我后来查了一下，可以这样翻，然后就直接这样翻了。因为后面这句比较长，一直到什么裸婚是一种什么什么的结婚方式。我就把后面那句是指一对就翻译成了我要带which的后置定语，你就来which来解释一下，然后这个事儿什么事儿，然后后面一句我调整，我调整了一下，就是把不对，我没有调整，我处理成一个主......就是他们主次不一样。就是due to 这个是我自己加上去的，我觉得是因为现在年轻人的生活压力大，所以我才会有后面这一个结果。还有我当时在翻的时候，我不知道最后一句话必须大肆操办婚事的传统，真的是不是真的要翻译成传统？我觉得可能是就是这个传统就指这种做法，这种做法就是这种做法现在在现实生活中越来越少了，但是我就觉得做法的话，跟那个传统的差别有点大，我就没有直接翻译成做法，我还是直译成了传统。第四段材料，首先拿到这个一看，我就要去查什么是一架波音767型飞机，然后就直接拿过来。用从波士顿飞往洛杉矶就完全直译，其实这个一直向西飞行，我当时还有点小纠结，这个是要用什么时态，现在一直就是那种现在完成进行什么，就表示他一直飞到那个状态，但是我就觉得这个事儿也是已经过去了，我就没有用飞过了这个山的上空，然后就直接是，我当时我本来用是pass还是用那个fly，但是我就觉得pass 好像是指汽车通过的哪个地方，好像平时很少跟飞机搭配，所以我就用了fly。其实这个突然掉头南下，我有这是我觉得这是一个难点，掉头南下，我就先把掉头翻译出来，然后再南下掉头turn round，然后南下down south。对，这就是大概就是我一个整体的翻译过程。

S9：

这一段话是我当时翻这段话的时候，我是觉得难点在于最后那部分就是从最后一句话，前面的我觉得还好，前面的我觉得就是因为他是说的全球气候变暖问题，我觉得就是要用那种比较稍微官方一点的语言，然后我就觉得语言上要注意语气的一致。然后后面主要是后面那一部分，后面那一句话，我开始是说的是那种理论需要经过不断的实践反复推敲，然后后面那个收集数据这句话我开始不会翻，我是放到最后来翻的，因为我想不知道这句话来怎么描述，开始我也不懂，然后我也想的是用的那种增词，然后把这句话把它翻出来的。因为他说的是test，然后我就觉得应该是收集跟数据有关的来表明这个气候变化，所以我就运用有很多增加增添词的那个东西来把它来把它说的长了一点。然后就说收集数据这么越多，然后才有利于什么反复实验，就这样的，然后最后我就是这样把它翻出来。然后第二段第二句话我看一下第二句话，对前面第一句话，那个数量那里确实我开始考虑到了，我觉得图书馆的数量，我开始也是在想用一个什么词来说明它就是应该是别人借出去了东西，然后没有归还，所以它会导致它下降，然后最主要的也是中间那里中间那个还要明确还要明确的一点，这句话我没有太懂，就是要满足别人的需求，这句话我没有翻的很好，我觉得因为我没有太懂这个意思，然后我觉得我这句话也没有翻清楚他是他所要表达的意思。然后后面这句话我觉得就是我把它综合了一下，就是关于借书，如果没有，到时间了没有还的话，就会罚款，然后我就把这两个结合在一起，他就会这种对于这种情况罚款的行为会加以严控，严格的管控，然后我就把它融合在一起了，最主要的就是中间那句话，满足其他学生需求。这里我没有翻太好。然后第三个，首先我想是裸婚，然后他后面也是对他的一种定义，所以我就先是先把它说出来，然后就用一个means就是来解释他这种行为，然后用了一个without，然后连接后面的什么都没有这几个短语，然后后面就分为了主要是两句话，前面一句，后面一句。然后后面这句话我想的是那里有点问题，就是那个传统的那种办婚礼的那句话，the tradition of 我觉得having to有点问题，这个就是这个问题，其他还好。然后第四句话我觉得我就是顺句挨着翻。首先应该注意的是什么。然后这架飞机从哪飞，带了多少名乘客，就是挨着翻下来的，然后就没有太大的我自己觉得这句这段话还好，没有特别太难的点。

S10:

第一句话就是比较普通的一句话，然后就直接就这样翻出来。然后第二句话，因为我觉得我第一次想到的就是这个take steps to，然后我想到这个take steps to的话，我就想了一些翻译。然后可能想到的最直接的就是采取措施，然后去阻止扭转，avert这个词的话我更倾向于翻译成阻止，这也是我的第一反应，这是因为我觉得这跟全球变暖和威胁比较搭。然后后面就是把这里有一个词性转换，把certainty 改成了理所当然的，这个也是符合我们的汉语的语言习惯。然后后面这句话我看到there are no certainties in science，虽然这句话是和前面一句话是分隔开的，但是我还是倾向于把它加到前面一句话，因为他们的意思，我可以说他们是在同一个意群里面的，所以他们的意思是有逻辑关系的，所以我就同时也用了逻辑关键词。prevailing theories 这个我根据我的经验，我把它翻译成了主流理论，因为在各种各样的文体中，比如新闻文体或者是报道中，一般都有类似的表述，所以我就用了主流理论这样的表述。然后最后这个关于实例来验证，然后最后一句话，就是不断搜集更多的实证，然后反复验证，这个都是我觉得这就是之前相当于我觉得应该没有什么翻译过程，这是比较下意识的翻译出来的一个结果。然后第二个it has been noticed with concern that 我觉得这里采用了一个技巧，就是it is什么that 加一个形容词，你这个地方这个技巧就是让人什么什么的事，或者说什么的事。我觉得我就用了这个比较普通的这样一个技巧，然后我就把它翻译成让人担忧的是。然后图书储存状况惊人的恶化，说实话这个恶化，我一开始我想到首先是这个储存的状况，但是我不太确定这个表示储存状况变坏的这个词能够能够用什么词来搭配，我就想了一下，然后我就想到了关于病情的恶化的这个动词，所以我就用了恶化，但是后来我再提交了译文以后，我又看了一下，我觉得可能还是不是特别的准确，所以这个地方我觉得是我可以再进一步润色的一个点。然后后面一句话，同样的我觉得也可以进一步润色，可以很明显的看到这句话里面，我没有加一个非常明显的一个主语，所以我后来我一开始甚至是没有主语的，但是我觉得没有主语的话，首先第一个就不符合汉语的表达习惯了。第二个，如果说我这个句子太长的话，很有可能会形成病句，所以我就加了一个规定。当然我觉得这个规定可能对于这个ask 的要求的这个含义来说的话，可能也没有非常的准确，但是我觉得就是因为在原先的这个句子里面，也没有一个主动主语，所以我觉得用这个规定来说，可能是一个对于我自己来说是一个中规中矩的选择。然后后面一句话时刻为他人着想，我觉得我翻的比较应该说比较general，因为我觉得把别人的需求考虑到自己的这个的话，我觉得这已经是属于意群的一个末尾部分了，我不想用一个太长的句子去说明一些...就是去说明这个内容，所以我就把它翻的比较比较笼统的一点。最后一句话，我觉得就没有什么可以，这个也就是一个比较直接的一个翻译，然后坚决落实，我觉得这个应该也是我从我们汉语的表达习惯出发然后来进行翻译的，这是第二个。然后第三个，裸婚是一种新型的结婚方式。然后关于这没房没车，不办婚礼，不度蜜月，这些都是一些term。然后首先我就去查询了一下相关term，然后确定了裸婚它所对应中的英文的表达是naked marriages，然后中间我就想关于这后面都是关于这个裸婚的解释说明，所以我就想把它通过从句或者是插入同位语，或者是甚至是插入语的方式，把它放在中间或者是放在后面来说明，所以我就想了一个我最近刚刚学到的一个表达，包括这些情况表达都是刚刚学到的，所以我就把它用了一个as加了两个逗号的，一个一个短的一个从句放在了中间，但是有一个问题就是我在翻译的时候，我发现因为这个里面有很多并列项，没房没车，不办婚礼，不度蜜月，所以我就把它简化了一下，我丢了一部分。但是我觉得这样的处理应该是差强人意的，当然这个词可能用的不好，可能还是需要进一步也是需要进一步提高的。因为如果有并列项的话，我个人觉得最好还是把它翻译出来，不然的话，在准确度这一方面，我觉得多少会有歧义。但是当时我考虑到了这一点，我又考虑到整个句子的通顺问题，如果说我要把所有的这些并列项加上去，可能我觉得我会导致我的整个句子逗号非常多，非常长，我觉得会失去我把它包含到一个从句里面中的这个我就失去了这个原本我想要达到的这个意义，所以我还是丢掉了一半吧。因为这个里面就是properties，就是相当于财产，可能这个也不准确，然后后面的wedding 就只有婚礼，没有蜜月。然后indoors 这个词是因为之前用过的，所以说就直接拿来用了，然后后面一句话，现代年轻人的生活压力大，且强调爱情的独立，在这个里面我想的是我先去说独立，因为这个独立是......我觉得这是一个在裸婚整个语言环境里，或者说这个语段的整个语言环境比较核心的一个意思，所以我想把它先说，然后后面再说在这个比较大的生活压力里面，所以这是我的想法，所以最后一部分就是最后一部分就是关于这个大肆操办婚纱的传统，在年轻一代中逐渐削弱了。然后这个里面有一个动词，我用的是mark，因为这个词我想到既有庆祝的意思也有就是recognize 就是把他看到一个很高的，重视他的意思，所以我觉得这个词我比较倾向于去在这里使用它，这是第三段。第四段，我想的一个过程是因为这是一个描述性的，是描述这个飞机的运动轨迹，所以我就首先会去想这个飞机它是怎么样去运动的，然后其他的关于比如说里面也说到了，从波士顿飞往洛杉矶载有81名乘客，然后飞过阿迪朗达克山，冲向纽约市中心，这些都是二级信息。首先我要去翻一翻的一级信息，就是飞机的运动轨迹，所以我就想的是，我先用主句来翻译这个飞机的运动轨迹，所以我就直接先说了这个飞机，先是引起了安德鲁的注意，然后再去加上他从波士顿飞往纽约，载着81名乘客。这是这首先是这首先是第一句话，然后又说他突然掉头南下，然后冲向纽约的市中心。不对，然后我又说他就起飞，然后向西飞行，然后掉头南下，冲向了纽约的市中心。然后我把它后面再加上在这之前它是飞过了阿迪朗达克山，所以我想的主要的就是通过我这个主句和从句的交替的使用来体现这个飞机的行动轨迹应该是一级信息，然后其他的信息是二级信息。

S11：

第一句话，我就是直接把那个global warming is a serious threat把它提到了后面就avert it 那个it 我就用来代替它。然后这样刚好一句话就说完，然后后面就是but那个地方就是直翻的，我感觉我后面基本上就是直接翻的。然后最开始那个prevailing 那个serious 那个地方，我把prevailing 那个单词还看错了，我最开始我看的就是那种先前的理论，第一个就讲完了。第二题就是然后这个就是这个句子，第一个句子我就是很明显的是我是这样翻的，然后这着实令人担忧，我就是把那个concern 提到了后面更加突出它的那种感觉。然后第三个我就是我也感觉我是直接翻的，我觉得我前面的这个从句，我是觉得我当时我是想不想用这个从句的，我觉得我们平时太喜欢用从句了，我当时想用那种现在分词，或者是那种用现在分词这样的句式去表达，我觉得我自己在翻译的时候，我觉得我比较排斥去用从句，但是我有时候又找不到说一个比较好的方法，却不用从句，所以我这里就很纠结，所以我感觉我这里翻的不是很好。然后在句式的那种连接上，我感觉那个逻辑关系，还有它中间的一些连接，我感觉我有时候比较难把握。然后这个high profile 我是之前做的专四的语法题之间看到过，然后就记下来了。然后就想着用这个词，然后eliminate 这个应该比较常见，就很多政府工作报告里面消除贫困，都用这个。然后第四题，我觉得我翻的时候，我就感觉我看完我自己会感觉好像小学生在写作文一样的，我自己翻的时候，我也会感觉就像你写那种流水账，我不知道要怎么处理好，平时接触这种题材的会比较少，然后我翻的时候，然后我翻到第四题的时候，真的不知道怎么处理，然后就很像小学生写作文，然后基本上就是直译的。

S12：

我自己在处理这个第一段的过程当中，我发现主要的原因就是如果在我能够理解，而且我自己认为我理解正确的基础之上，我觉得最主要的应该英翻汉的一个难点应该就是他的措辞方面的问题。就比如说第一段的话，我想将believe 后面的两个从句以及后面的那个we do not believe that 那三句话连合并成一句话来翻，但是由于我自己的那个措辞功底，我觉得这三句话并不好翻译成一句话，所以我最后还是按照原文的一个原文的断句方式，然后将第一句话就直接翻翻成了那样，后面的那个but we do not believe 这一句话，因为我觉得英文当中他们的否定句是放在主句当中进行一个否定，但是按照中文的一个语言习惯问题，所以在这里我还是将这个not 放到了它后面的一个从句进行翻译。再就是我个人认为这篇文章，这篇小段落的理解并不是特别难，它主要的措辞问题，我个人觉得体现在一个certainty 和一个prevailing 处理方面。在处理certainty 的时候，它其实就有什么确定性以及什么定数这种词语的选择，我个人在这里就用了定数，是因为我觉得比确定性而言的话，在中文二字格四字格是最为常见的。然后再就是后面那个句子，there are no certainties in science，在这句话的处理的时候，我如果直译的话，它就是科学当中没有确定性，那么我觉得体现的意思就是科学本身就是不确定的，所以我就这样翻译。我个人在觉得难一点的处理的难点就是prevailing 这个的处理，它有许多词语方面的选择，什么受欢迎的什么，像是popular, widespread 这种同义词，都可以来表示一种欢迎。但是我觉得英语是一个比较抽象型的语言，而汉语是一个比较具体的语言，所以我更想体现的是他受欢迎的，尽量把它表现的更加具体，所以我用了一个广为人知，但是在这里的话，我觉得这个选词我自己其实也还有待商榷。最后一句话，是一个省略句...这应该是一个省略句，就是more evidence must be collected and the theories must be tested again, 还原之后再进行翻译，所以这就是第一段话。然后第二段话的话，我个人还是觉得是又是措辞的问题。as we noted with concern, 在这里是英文当中习惯把主要的部分放在前面，所以我翻译的时候是把它处理到后面去的。我就先阐述了这个现象，然后再把这个alarmingly 单独拿出来做一个评论，然后再翻译它的一个主要部分就是引起公众注意的同时，也让人产生了担忧，再到后面的Students are asked to remind themselves of the rules for the borrowing and return of books. 在这里的话，我觉得没有什么。后面的话就涉及到了又是一个英语的抽象性和一个中文的具体性的一个问题就是to bear in mind the needs of our students 什么样的needs，我觉得在英文当中，虽然可以根据这个context 可以推出来，但是我觉得在中文当中我们的语言习惯问题，我还是把这里的need 给详细的处理了一下，就增译了借书这么一个词来更加的概括。再就是penalties for overdue books in the future will be strictly enforced，这句话的话，我觉得penalty 我们说是惩罚措施，将会采取什么样的惩罚措施。那么对你的对象我也觉得是有必要把它点明出来的，所以我个人处理的时候也就增译了学生，所以就是处理成了一个有学生再怎么样，我们学校就会怎么样，我觉得这样子逻辑关系会更加清楚。汉翻英两个段落的话，我觉得主要也就是一个合并的问题，裸婚是一种新的结婚方式，这就是一个一个典型的中文的一个主题论述的一个方式，然后在这里的话，英语当中我也不知道是因为学口译的原因还是怎么样，就很习惯的就顺句驱动的，我就可以把它翻下来，我就这样翻了，就是naked marriage refers to a new way of tying the knot我之所以会选择tying the knot是因为我看到外刊里面还是蛮常用这个词来表示结婚，我个人也觉得非常形象，所以就采用了这么一个词。然后后面只一对恋人没房没车这样的一个概念就是很典型的一个阐述它的概念我觉得就一个定语从句就可以把它概括下来，然后的话也没有什么特别大问题。再就是现代年轻人的生活压力大，且强调爱情的独立，必须大肆操办婚事传统，这就是典型的中文当中，他们擅用那个动词，但我觉得英文当中我只需要一个动词，然后其他的可以用一些什么同位语，非谓语来表达出来的，我都可以进行这样一个表达。我个人觉得他这里这句话，他最主要的还是在强调什么和逐渐削弱了什么这两个动词方面。所以我自己处理的时候，把年轻人的生活压力大，我就处理成了一个从句，然后后面就用了put priority over the independence of love。在这里我就个人有一个疑惑，且强调爱情的独立。这里爱情的独立是指的什么？独立是指两个人的经济方面的独立，还是说什么什么的独立。但是后来一想，反正因为是一个比较抽象型的语言，他汉语当中给的独立，我就直接对应一个independence 就行。然后其他的方面的话就是逐渐削削弱这一个选词问题上，其实也能对应到一个词，我就用的undermine，然后大肆操办的话，其实也有很多相应的词组，像splash out on...... 我觉得都可以用在这里。然后再就是第四句，首先引起安德鲁注意的是一架波音767型飞机。我没有想到我居然卡在了首先引起什么什么的这个上面，我刚开始处理的时候是处理成了Boeing 767 attracts the Andrew first,但是我觉得这样翻译是有歧义的。因为他如果这样翻了的话，他就是首先引起了安德鲁的注意，后来可能又会再引起别的什么注意，所以我最后还是用了一个比较普通的用词，was the first thing that attracted Andrew? 然后这里的话，我这段话我觉得还有的讲是因为他这个句子处理，我觉得有一个合并的成分在里面。这样像后面这架飞机从波士顿飞往洛杉矶，载着发生一名乘客怎么样，这都是一段对于这个飞机要干嘛的一个陈述，我也觉得不是一个非常重点的话题，所以我又用了一个同位语把它带过去，而且我觉得这个同位语用进去的话，它不这样的一个主要的成分，而且还能把这些意思都能表明，所以我在这里就插进去了a plane scheduled to take 81 passengers to LA from Boston。然后我的处理就把一二句话进行了一个整合，然后再到后面飞机上午7点59起飞，我也就直接是taking off at 7：59，然后一直向西飞行，这里一直向西飞行，我当时脑海里面就直接是straight to，所以就是用了flew straight to the west。飞过什么什么上空突然掉头南下，在这里飞过什么上空，我觉得不是一个主要成分，它后面的主要成分应该是掉头南下和冲向纽约市中心，所以在这里我也就处理成了出个took a sudden turn to the south after flying over the 什么上空，我觉得有点难，一个地方是在掉头南下，这个南下是个什么南下，这是我向南非回去了，还是说我向南非回去的过程当中同时也有下降，所以在这里我也不太能够确定，然后冲向纽约市中心，这里的冲向那个选词太难了，我又想用crash的，但是plane crash一般是指飞机失事，它这里还没有强调出它是一个失事的状态。所以我不想就不能用crash，然后要用体现出它的那个冲的那个趋势，我也想到几个词，什么flock，但是flock 它又体现的是鸟群一群飞过去的感觉，所以我觉得flock 也不行，后来我就只能又想体现出这个意象，最后选词就选用了一个rush，但是我还是觉得不太贴切，因为这毕竟是飞机。
