# Supplementary material for: Assessing Students’ Translation Competence: Integrating China’s Standards of English With Cognitive Diagnostic Assessment Approaches
Source: Front Psychol. 2022 Mar 31;13:872025. doi: 10.3389/fpsyg.2022.872025 (PMC9008137; doi:10.3389/fpsyg.2022.872025)
Supplement: Supplementary file 3 [file Data_Sheet_3.DOCX]

Supplementary Material

***Transcript of experts’ verbal reports***

Texts that help identify different attributes are presented with different colors: conveying key information; conveying details with accurate wording; conforming to language norms; conforming to language habits; reproducing styles; optimizing logical structures; using the strategy of execution

E1：

第一份译文需要注意的是，英译汉最重要的是要去切，就是把这个长句要把切成几块儿，因为我们汉语的话是很多流水句的。然后你可以看到很多逗号是吧？其实在这儿的话，我们认为不是大碍，但是我觉得我们认为后面如果加个逗号的话，可能更像汉语的那种形式结构，但是这个没有很大的问题，加了之后会更好。一大严重威胁是不是会比一个要好一点，这是我的看法，然后不要一个可以吗？另一个就是可能这个语法翻译的思维还是要克服一下，在这里面变成一个严重的威胁，我很少听说一个威胁，我只听说过一大威胁或一种威胁。然后世界需要采取措施避免全球变暖，世界需要采取措施，还是说世界各国，世界怎么去采取措施了？世界是一个非常general的，然后是一个集合名词，就像people，它可以指民族，也可以指人民，也就是各族人民。这个world 也是一样的，它可以指世界，也可以指世界各国。所以在这里我可能我觉得世界各国会不会更好一点，世界各国需要采取措施避免全球变暖。我们英文中很多代词是因为怕重复，它要用指示代词把它指出来，但是就这里肯定是不能翻译成它的。全球变暖说白了是一个问题，我就去说这一问题，而且这一问题又回扣了前面的全球变暖，它又一个回扣的感觉，能够把前面这个句子和后面这个分句把它的关系联系的更紧密一些。因为英语不像汉语，我是需要关联词把它突出出来，因为它是形合。但是汉语的话他很多时候都是欲言又止。当然，如果能突出层次感最好，但是如果你不确定这里真的存的确存在这种关系的话，那我不加也无妨，我觉得没有大碍，至少我这是一种策略。然后的话prevailing theories 这个prevailing 可能有点曲解，它不是主流的，就是说盛行的理论和主流的理论还是两码事，就是盛行的理论，它不一定主流的，盛行就是流行，只不过我们在这儿用流行的话，这个语域上有点违和。这是一个比较严肃的，然后议论性质的一种东西，如果是盛行和流行两个词的话，盛行可能更符合这个场合。第二段他说的这种语气是怎样的。然后他说的这种场合是怎么样的，就是说是谁说，肯定是图书馆的管理员或者老师说的，它代表的是全体管理员的这种语气*。*从全体管理员的视角对学生说的，那既然张贴在图书馆，那这个图书馆是我的，那我完全可以我们发现我们注意到什么什么。现提醒大家不是要求，remind是提醒的意思不是要求，要求是demand 和request，这个词从意义上来说有那么一点点偏离，还是要严谨一点。再者，我这个告示就是要简洁，然后就是那简明扼要，然后给人的感觉是我从图书馆管理员口中说出来的。现提醒大家，且记住其他学生的需求，我觉得这个没有很大的问题。**但如果说你要把它更明确化的话，就是说要记住其他同学也需要借阅，可能会更我们有一个策略叫做explicitation，叫做显化，其他学生的需求中这个需求到底是什么？它就是借书，借阅，所以这里的话记住其他同学也需要借阅可能会让目标语读者更明确**。第三段的话三个问题。第一个就是说我们看第一句话，你翻译的是naked marriage as a new way of marriage refers to a registered couple without...。我们用SVO把它，把它主干抽出来。你的意思就是说naked marriage refers to a couple, 有问题吗？婚姻是指夫妻，这个逻辑上就有问题了。就是在翻译的时候，尤其是中译英的时候，一定要还原成SVO，看一看有没有语病，这其实也是一种语义上的语病，语义不通*。*然后这里的这个house, car, wedding, honeymoon，就是说我们单词的话，尤其名词，我们一定要说注意它的这种单复数。它如果独立存在的话，它要么是单数存在，要么是复数存在。然后再就是modern youth，modern youth 这个有这样的表达，但是从风格上来说，这种是比较轻快，就是我们平常比较轻快的这种散文。但是这篇文章说实在他不是散文，它是一种生活大众化的说明文，那这个时候我就不要去搞那些文艺的东西进来，我觉得young people就行了。你不要觉得这个youth好像比这个young people 更美，要注意场合的。然后这个extravagant marriage，表示很浪费，很奢侈。大肆操办它是有区别的，大肆表示的是一种规模之大，grand scale比large要更好。因为grand就表示一种庞大宏大。宏大的话给我的感觉就是这个婚礼很宏大的感觉，比这个large 的这种色彩会更强烈一些。on the grand scale如果是细究的话，extravagant 你可以浪费奢侈的，它跟大型的好像没有大的区别，其实有区别。浪费奢侈不一定会很大型，没有必然的一个联系的。然后再就是in generation，这个是语法错误，应该是among the generation。最后一段其实他考的就是怎么去把很多分句整合成一个结构紧凑，然后符合形合特征的这种英语的句子。这个时候我就需要借助非谓语。

对于第二份译文，首先这个世界各国翻的非常好，就是说很多人就翻译成世界，但是如果是翻译成世界，是说不通的，所以说这个世界各国它其实也不算一个增译，它其实就把它具体化，使之更符合语言规范。然后我们再说它的发生，我们原文是it，从常规思路来说的话，it它在英文中代词，但是我们在中文中的话，我们是喜欢把代词把它对应的名词直接说了，把它对应的名词把它说出来。那对应的名词是什么了？是这个事情的话，这个是什么？是全球变暖，避免全球变暖的话，对，就是全球变暖的意思，对不对？但是这个意思也算具体化。对我们就是说我们英文中喜欢用代词是怕重复。但是我们中文的话我们是不喜欢用代词。我们很多时候是把这个代词所指代的那个名词或者指代的那个事情把它说出来。这个we don't believe，we don't believe climate change is a certainty. 这是一个否定转移，这个否定转移他他其实应该not，应该是后面的，但是英文的话，他把这个not 否定就转移到前面去了，所以说我们翻译的时候，我们要还原。然后接下来一些普遍的理论，这是一个误译，prevailing它更多的是盛行的。第二段其实最重要是要考虑这段话应该出现在什么样的场合。什么样的场合就决定了行文风格。然后再就是那个学生被要求这个被也要转成主动，以符合中文的表达习惯。下一段的话，整体来说，还是不太好。英文的话这个句子还是不要太松散了，能够把它整合起来，就整合起来。然后现在年轻人的生活压力大，什么什么强调独立必须什么什么什么。这明显就是两层，一个是年轻人怎么样生活压力大，强求爱情独立。然后传统就在这种环境下，受它的影响逐渐削弱了，所以说还是可以用因果关联词连接的。然后weaken是削弱，decline 慢慢消失不一样。最后一段话其实最后一句话我觉得还是句式松散了一点点，没有逻辑感。

学生3的译文的话，总体来说我觉得还是不错的，可能个别的用词和个别的句子有点别扭，有两处可能是误译。这个prevailing 的话，它的本身的意思是普遍的意思，普遍的在这了，放这肯定也是没问题，但是就这个正式的程度来说，因为这是一篇论述文，论述文的话它的特点是比较...观点是比较鲜明，整个语义的话是比较偏正式的，也是比较严谨的那种，如果把preventing 换成比如说盛行的理论，搭配起来会更地道，也更符合这个语域。然后这个we believe，我不建议删除，删除的话是删除了冗余的东西或删除可以存在也可以不存在东西，但是在这的话，其实为了突出论述我们的一个特点的话，我建议还是加上，我相信...我们也相信，这样的话就能够让读者也找到你的一个标志，从信号词看出你的这个观点到底有几个。第二段的话其实除了最后一句话念起来有点别扭之外，其它都挺好。第三段的话意思也没有很大的问题，但是从基础的句法的正确来说，第一句话as a new way of for getting married , naked marriage is with marriage certificate only , and without 什么.....这段话其实不够正确或者不够准确，is with...is without . 可能你说主谓主谓宾或者主谓表都在，但是我们其实很少说把这个介词短语作为一个表语成分，就是单纯的介词短语作为表语成分，其实在我们地道的英文中是很少使用这样的。最后一段话想去整合，但是可能整合的不好，有这个整合意识就非常不错的。它这个是零散的分句，它考的也是你去整合的能力。原文的话是三句话，用这个句号来分的三句话。但是有时候我们不一定按照英中文的它这种分句，我们有时候完全可以把它拆开，然后去去整合。如果分成三个层次的话，怎么分？第一个层次肯定就是说这个注意的是令人注意的是这个飞机这个飞机从哪儿飞到哪儿，这个肯定能整合成一段。最后我们中间不看，因为中间有点难的情况下，我们就先看第三句。第三句我们是怎么整合？第三句我们可能就是强调它一个轨迹，飞行的轨迹从哪开始飞向哪飞，飞过哪，然后怎么样，最后冲到哪。好，也就是从一直向西飞行到最后可以是一个层次，因为我就是强调方向，因为前面是9点59是时间，它可能跟它不是一个层次的。就是我强调的是方向感和地点轨迹，而不是时间。所以说我们就从一直向西飞开始到最后。中间剩下的两句话，一个是载着81名乘客，一个是从几点飞。那想一下，能不能把这两句整成一句？其实可以的，这个完全也是符合我们常规，就是说它几点飞，载着多少乘客，这个载着就用with，因为如果说你把这个载着多少乘客放到第一个层次的话，那第二个层次就只有一句话了。

对于第四份译文，这个全球变暖其实已经在发生了，我不是去预防，而是去阻止，或者说去避免它继续发生。第二句话其实we don't believe 并不是我们不相信，它是一个否定转移，我们相信气候变化不是必然的。certainty 是必定的事情，它不是已然来临的意思。然后prevailing theories它不是当前的意思，而是表示一种盛行的理论。然后后面这个逻辑抓不清，后面more evidence collected....就是一旦收集到新的证据，就需要对其进行再次检验，这个逻辑就是这样的。第二段的话，还是从这个意思上来说，就是说这个贮藏量用的不太恰当，可能就是我们说库存比较好，图书馆一般就是库存。然后“我们”翻译的很好，这段话可能就是出现在图书馆的门口，然后是以这个图书管理员的这种语气去说，那完全可以用我们然后面向学生去说。第三段话的话，语法错误还是挺多的。which means means 后面就应该是一个完整的句子，A couple getting married ...这个是没有动词。后面的在年轻一代人中，这个应该是among，不是in。大肆操办婚事，这个celebration 只是单纯的这种庆祝，它还是不够准确。最后一段话它其实就是一个让你整合这个分句的，但是这个译文没有很好地按照层次整合。

第五份译文，首先avert它不是tackle，也不是deal with，也不是face，它是avert 表示一种避免的意思，或者说阻止都行，但是这个“应对”可能就不太准。另外如果英文中是it 的话，我们一般的需要把它的这个具体的意思把它还原出来。但是你如果说全球变暖是重复的，那全球变暖它到底归根结底它是个什么？它是个问题，所以说因此需要全世界各国避免世界各国采取措施竭力避免这一问题。然后第二就是我们也相信,我们也相信是什么呢？这个可能理解有误，这个climate change is not a certainty是个否定转移，它应该在后面，就是说气候变化它不是必然的，然后后面是什么意思？由于没有科学依据，这个不是这个意思，它说的是there are no certainty in science，意思就是说科学中是没有什么是必然发生的，也就是科学无绝对科学无绝对。第二段与之前几个译文的问题一样。第三段的话可能我觉得要注意一些这个句式，英语的句子虽然有时候要比这个中文要紧凑，但是紧凑的意思不是冗长，就这个你可以用一个很经典的句式把它凸显出来。

第六个译文的第一段话“我们认为全球变暖严重威胁到了整个世界需要采取措施，以尽量避免它对世界造成伤害”，其实就念起来是挺通的，然后也实现了中文那种顺畅和自然，但是可能有点过度的去处理原文了。原文他想表达的意思原文是说，原文其实就是一个议论文的一个段落，它有两个观点。第一个观点就是到这个advert，然后第二个就是说we don't believe 到最后。第一个点的话就说我们相信全球变暖就是一个严重的威胁，然后世界各国需要采取措施去避免或者去阻止这一问题。这个it是人称代词。在中文中我们要把它具象为这个具体的东西。但是我们它具体指的是全球变暖，但是我们不可能又重复说，避免全球变暖或者说阻止全球变暖，而是要把它这个全球变暖到底是什么呢？它是一个问题。这个考生的译文虽然意思听起来好像也ok，就严重威胁到了整个世界，即动词化了，然后对世界造成伤害，这个伤害有点过了。怎么叫伤害？就这个伤害本身它是一个非科技术语。因为这是一个科技类的，或者说涉及到环境类的这种议论文，可能伤害的话不符合这个风格。然后的话因为原文是说的是try to avert it，那就直接说去采取措施尽力避免这个问题了，所以总的来说可能这句话有点处理过度。第二个其实就是we don't believe that 这是个否定转移，英文中它会把这个否定词放在前面，但是他实际的是在后面，所以说我们还是要翻译成我们认为我们也认为，就是跟前面进形成一个递进。然后盛行的理论翻译的很好，它比普遍要好。第三段首先是一个句式的选择，我们首先要去加工处理一下。第一句话，裸婚是一个新的结婚方式，指什么什么的结婚方式，说白了他就完成了两件事情。第一件事情他就归了一个类，就裸婚是什么？给他给了一个大范畴的一个性质，它是一种结婚方式。第二个就是去下定义，去解释到底这种接触方式是什么。as ...它是都作为这个性质展现的一个最好的句式，所以这里也可以as a new way of marriage，然后naked marriage means...这个句式的选择很重要，比这个译文里的句式要好一些。然后是pay attention to, 不是on，这个是基本的语法错误。

第七个译文除了一些细节，然后除了最后一段的最后一句话，翻译的不太合适之外，我觉得其他的都翻译的非常好。其实我们这个global warming is a serious threat，这个我觉得完全是可以直译的，就是全球变暖是人类面临的严重威胁。然后世界各国翻译的非常好，而不是世界应当采取措施避免这些问题。然后我们认为...我们也认为也能够突出这种递进关系，然后科学无定论，盛行的理论必须不断接受检验，非常好，然后收集证据再进行检验，这个逻辑关系就有问题了，前面这句盛行的理论必须不要要得到正确的检验，后面不是然后，后面是一个解释，后面说的就是为什么要做到检验。后面的意思就是说一旦有新的证据，那就要对理论进行再次检验。第三段的话，可能就是说整个结构完全是没问题的，用as也非常好，但是还是一些小问题。比如这个get 已经用过一次了，那这的话我就用动词不行吗？我就是marries each other，我能用married ，我为什么要用get married？which means a couple marries each other by only ...中间差了动词。这个wedding 在这可能要把它细化成wedding ceremony，婚礼和婚姻不一样。然后as young people，然后细究一下，这个the是多余的，就是the是特指，你这个完全就是泛指，所有的年轻人as young people 可能到这个细节的这种定冠词和冠词的这种用法，还是要去回去加强回顾一下语法。Freedom of love, 这个freedom 是自由，独立是一种independence，自由跟独立不一样。然后is gradually diminished，首先这个语态有点问题，说实话tradition 它是一种能在这种前面的这种年轻人的观念环境下，它能动的自然而然的一种主动的一种削弱，而且这个diminish 它很少是说使用被动diminish就表示了一种主动的减少，它更多是数量上的减少，但是削弱是表示它程度的削弱就是weaken，那就直接说the tradition 怎么样weakens，或者说has gradually weakened among the young generation。如果把这些细节改变的话，那这个英文就很完美了。总体来说翻译的非常棒。

现在评价第八个译文。众所周知，全球变暖是一个很严重的问题，全世界各国都必须采取行动改善这一问题。大的问题没有，但是可能细节的措辞还是需要注意一下。严峻的问题跟威胁又不一样，威胁的程度是非常高的，问题可能是小问题还是大问题，然后这个威胁还是要把它还原出来，就是说是一个很严重的威胁，这种threats 和problem是完全不一样的两个词，不要随意去改，我们要以忠实为前提。如果你忠实的不到位的话，其他什么我们都不会看的。世界各国翻译的很好。这个必须错了，need to 是需要，需要不等于必须。改善是improve，这个avert 表示一种直接的去阻止，去避免这个问题，改善是指本来还没有那么糟糕，就在这种还可以的基础上去给它一个提升，这个是改善。但是阻止和避免是你本来这个问题就是坏事儿，我一定要去避免它。“这个问题”翻译的很好，你要把它还原成全球变暖，但是全球变暖就不能重复，所以说全球变暖它归根结底是一个问题，这个翻译的很好。然后下面一个从科学研究的角度来看并非存在什么必然因素,这个有点啰嗦，它其实就表示科学界中没有必然的事情的，就是科学无绝对，没有说什么科学研究的角度，这个太啰嗦了，就直接说就是科学里面是没有必然的，那就是科学无绝对。然后普遍理论需要经过不断的实践，这个不是实践，而是被检验。看第三段在句式上要更提升一步，显示一种逻辑关系。the tradition of having to have 这个有点怪，你可能就觉得我就直接把去掉一个，去掉之后，又有一点损失意思。那这个时候怎么办呢？可以把它变成一个从句，就是the tradition that 去解释这个tradition。

第九个考生没太分清楚笔译和口译的区别，他很会用一些口译的策略，比如说generalization 这种概括化，就是把它一些细节把它概括，汉译英最明显。其实在笔译中其实不应该这样去处理。尤其是像第三段，因为第三段它本身就讲的是一个裸婚，然后给它一个非常细节化的定义，让我们让读者更清楚，这个裸婚到底从微观来说到底是个什么样的东西。人家本身就是想给你通过细节化的方式来给你科普裸婚是什么，那generalization我显然是不合适的。这个地方丢的东西太多了。然后naked marriage is a new way of getting marriage，which refers to 这个语病来了，refers to 他后面不加句子，如果你想解释定义的话，你就说which means that 那就可以了。which means a pair of lovers，你直接说a couple 就好了，而且lover 是情人，情人跟恋人不一样，情人突出的是一种比较不稳定的那种感情，couple比较正儿八经的，也是比较中规中矩的。后面有很多漏译的地方，这个modern times 哪去了，然后这个生活压力大中的“生活”去哪了？另外，diminish 它主要是指数量的一个减少，但是对于这种程度的削弱可能还要用weakened比较好。还有后面的这个marking，虽然有庆祝的意思，有标志的意思，但是原文中的大肆操办，他强调的是个什么？这个大肆操办，它强调的是一个规模问题，就是搞得沸沸扬扬，然后规模比较大，它强调hold marriage on the grand scale，然后你用这个mark，就是庆祝婚姻，庆祝婚礼这个意思是对的，但是没有把这种规模大的意思表现出来。第二个我想提一个状况惊人的恶化，我第一次听说图书的储存状况惊人的恶化，我只听过就是个病情，病情恶化、环境恶化，但是储存图书的储存状况还可以恶化吗？而且为什么一定要是状况，为什么一定要把这个状况说出来了？他强调就是一个图书库存量的问题，图书库存量在下降，然后它急剧的下降就可以了。我们看第一个第一段的话，人们需要采取措施来阻止它，这个也是比较细微的一个东西，英文中我们喜欢用人称代词，但是在中文中其实我们很少用的，除非是他做主语，比如说他是个什么样的人，他是个什么样的人。但是如果说我把它放到宾语这个位置的话，其实很多时候要还原，当然不是还原成原原本本的全球变暖，而是要把他的性质说出来，它是全球变暖，全球变暖是什么？是问题是现象，所以说人们需要采取措施来阻止这一问题。

最后一个译文，首先世界如何采取行动？第一句你是把它合起来了，但是中文中的话，反而我们是要把它切出来，不然这个句子太长的话，会有翻译腔那种嫌疑，这个译文不太明显，但是我是比较希望就按照英文的意群来处理，首先说它是严重威胁，再说世界各国需要采取措施来应对，就是说我们认为全球变暖是人类面临的严重威胁，世界各国需要采取措施避免这一问题。avert it 要把它还原它，而还原成全球变暖，但是也不要重复全球变暖。公认的理论可能就有一点点意思上的不准确，prevailing它实际上是一种普遍的或者说比较流行的，但是如果在我们这个语气之下，我们这个语气是科技文的语气，这个语气比较严谨，比较正式，流行的话是偏可能偏那种非正式一点点，如果说当然也没问题，但如果说我们要让这个措辞更严谨更正式一点点，可能我们要用盛行比较好。然后第三段的话，首先我们要看第一句话，当我们遇到这样的句型的时候，其实我们一个很好的可选择的句型是as ...按照这个句式来说，就是a couple get marry or a couple marries each other by only applying for a marriage certificate without ABCD，这样的话句式的话就比较紧凑，而且比较自然。然后就是as表示一种因果关系，因为这个年轻人他们生活有压力，他们强调独立，所以说这个tradition 怎么样了...high profile 是什么意思？high profile给我的感觉就是比较高调，高调可以，但是说实话我觉得在这里有点过。我觉得就是说这个大肆操办，跟高调之间到底是不是能够对等这个我不敢说。但是我认为在这的话大肆操办，他强调的是什么？强调的是种规模之大。他强调是一种grand scale，就是搞得沸沸扬扬，就是说整个排场非常足，所以最准确的应该就是说这个就是the tradition that wedding ceremony should be held on a grand scale。eliminate 它是表示是一种消失或者排除或者淘汰，但是原文是削弱，削弱的话，可能他是从程度上来说是一种weakened。

E2：

Prevailing theories 翻译为假定好成立的理论，首先意思并不是百分之百的正确。第二，假定好成立的理论在中文中似乎并没有这种说法，比较这个说法比较怪。还有and 翻译成并且以及more evidence collected，而当更多证据被收集时，都比较Chinglish。

这段话翻的比较好的地方在于prevailing theories 翻译成流行的理论，这个比较像是中文的表达方法，但是有一些地方就表达不是特别的到位。比如所有的被动模式也就是be tested，be collected和be tested，它都翻译为被检验、被收集、被验证。这个翻译没有第一个同学翻的好，第一段是把be tested 翻译成经受检验。

这段翻译总的来说翻的比较一板一眼，很多词汇就是按照他词典里的含义来翻，但是并不适合译入语，也就是中文的表达习惯。比如说prevailing theories must be constantly tested，这个constantly，它翻译成持续的用证据来检验，持续没有问题，但是不断的是不是好一些。另外有一些地方译错了，prevailing theory 翻译成前瞻的理论应该是比较流行的理论。

这一段翻的比较好的地方在于有一些be done，它翻译为比较自然的表达，比如说be tested，be constantly tested against evidence, 就是不断的接受证据与事实的检验。但是它的不足之处在于，有一些地方误译了prevailing theories，翻译成现有的理论。第二。欧化现象比较严重，比如说第一句。第一个是两个短句，它把它翻译成一个非常长的句子，这个也是不符合中文的表达习惯的。

这一段翻的比较好的地方也在于它那个被动式翻译成自然的顺句的形式。比如说and more evidence collected and theories tested again，而收集了更多的证据之后，就需要再对理论进行检验。这个没有把be done翻译成被，还是比较灵活的。但是也有误译prevailing theories，它翻译成之前的一些理论。第二点是有些地方翻译的意思并不是百分之百的正确。比如说there are no certainties in science，它翻译成科学是未知的，不确定的。这个未知的和no certainties 还是有一定的语义上的距离。

这一段表达非常的自然，很符合中文的表达习惯。比如说第一句，第二部分the world needs to take steps to try to avert it。世界需要采取措施来扭转这个趋势，把avert 翻译成扭转，并且加了一个原文中没有的宾语“趋势”，看起来像是加了一点东西，但是实际上意思是跟原文是一样的，而且表达也更加顺畅，这是成功的增译。其次，它的被动式也是并没有翻译成被怎么样，而是把它翻成这个通过什么来验证，证据收集的越多等等。但是有一个地方就是prevailing theories 没有译出，应该是明显漏译了。

这段话有一个地方也是误译了prevailing series 翻译成先前的理论。另外他的被动式也翻的比较的死板，都翻成被验证，被收集。

这一段prevailing theories 翻译正确了，翻译成了流行的理论，但是有些地方不够准确，或者是表达不够地道。比如说a serious threats 翻译成严峻的议题，threats 应该是威胁和议题还是有区别的。这个翻的不够准确。第二，有些地方表达比较欧化，比如说全世界需要做出尝试，避免它的措施，应该表达成全世界需要采取措施来尽量扭转这个趋势。还有就是被动式都翻成了被收集被检验，这个表达不是特别的地道。

这段话有些地方表达不够地道，比如说阻挡全球变暖，阻挡应该是和趋势搭配，或者是阻挡一个什么行为阻挡全球变暖，似乎这种表达比较少。第二个，有些地方翻译的不够精准，比如说there are no certainties in science，科学界不存在一成不变之时，这个语义上有一点偏差。然后有一个地方被动的翻成受到考验，这点翻的还不错。

这段话表达比较自然，比如说理论必须接受证据的检验。越多的证据被收集，理论也就再次受到检验。并没有一五一十一板一眼的把被翻成被动，被怎么样。第二个。avert it也翻译成缓解这一现象。总的表达还是比较自然的，但是prevailing theories 翻的不够准确，翻成了现有的理论。

E3：

一号同学的译文，整体上把意思翻译出来了，但是有一些细节需要注意，就比如说第一句话中，原文的时态是有的，has been应该就是现在完成时，所以最好表明一下这个句子发生的时间。比如说近来、最近都可以的，但如果你不说的话，就不知道这句话发生的时间到底是什么时候，有可能是过去，也有可能是现在。这个学生另外一个问题就是他虽然明白了原文的意思，但是他这个翻译腔还挺严重的，也就是跟原文跟的太近了，就比如说第二句话，学生要提醒自己借书还书的规则。这里原文是students are asked，学生被要求去提醒自己，我们就会想这是谁要求他？所以从逻辑推理来看，应该是图书馆要求，所以正式一点的话，最好就是说现提醒学生怎么样，这样就既把这个主语谁提醒这个主语就暗含出来了，现提醒就非常的文绉绉的，就觉得可能是这个图书馆这边去提醒学生，而不可能是学生自己提醒学生提醒他自己。所以学生这里不仅是这个语言太贴近原文，跟着原文走，而且他这个意思也稍微有点出入，没有翻译出be asked。这个是谁？这个是施动者是谁，然后后面这半句他说学生说并时刻记得其他学生对书籍的需求，所以这句话倒也把意思翻译出来了，但是就还挺费解的。你这样说还不如直接把这句话的意思说到位，你就直接说，别忘了其他同学也有借阅需求。特别是我们还要考虑到这几句话的语用环境，如果是张贴在图书馆门口的这种告示，如果你说的这么的委婉，那学生可能看完之后还是很懵，不知道你要说什么。然后最后一句话，未来这个词听起来还挺远的，就像科幻片那样，所以我感觉今后听起来就是the near future，而不是the future far away 特别遥远的未来。还有学生这一点非常有趣，这个逾期还数的赔偿金，我们一般说逾期不还对吧？我们不说逾期还书，如果你还书的话，那再说你逾期就不太对，对，逻辑上好像有点问题。一般逾期的话，就代表你到时间你还不还的。如果你还书的话，那你所以这个地方很奇怪，它这个中文就没有转换过来，受原文的这个影响太重。逾期还出的赔偿金将会被严格收取赔偿金。这个我觉得penalty 是赔偿金吗？penalty 可能是罚款和赔偿金应该是有区别的。如果说到赔偿金会让我想到法律中的一些纠纷之类的。因为你图书馆，你的书只不过是过两天晚一些还这并不涉及到你赔偿给图书馆什么图书馆也没有损失到什么。只不是说给你一个处罚而已，因为你影响到别人了。被严格收取那这个也是紧紧随原文的，我觉得参考答案处理的比较好，就是照章处罚，会按照规定进行相关处罚。这听起来都还可以，对其相对来说更加的地道一些。所以这个学生他的英文水平还可以把这个意思翻译出来，但是一定要注意自己这个中文有待提升。还有要注意到这句话的语用环境在哪里用的，它的目的是什么？如果这样，仅仅翻译出意思，而这个语言很费解，让人一下子看不出来的话，那这个翻译还是一个失败的翻译。

二号同学的译文相对比较简洁，意思也都翻译出来了，但和一号同学一样，这个中文水平还是有待提升。首先这边alarmingly他翻译的是令人警觉的急剧下降，你会发现这两个词在中文中我们不这样用。我们说图书馆藏书的数量急剧下降，但不会说令人警觉的就急剧下降。听起来很怪，还有像后面这半句，这引起了人们的注意和担忧。开始学生用的是注意，但是他觉得这个注意是一个中性词，觉得不过瘾，后面又加了一个担忧，但你这两个词都放到这儿，就稍微有点啰嗦。所以你把注意删掉就可以了，因为这个担忧自然里面就包含了注意了。下面这一句，学生们被要求提醒自己注意借还书的规定。前半句非常的怪异，学生被要求提醒自己，那这到底是什么意思呢？被谁要求？这里可能学生还没有掌握这个公文，比如说写告示，写通知这种写作手法，所以没有意识到这里其实可以现提醒大家注意借出还书规则，用现提醒就避免了被动语态。我们中文中这个被动语态是比较少的。后面这半句并把其他学生的需求铭记于心这个铭记于心这四个词倒也翻出了bear in the mind，但是感觉这有点太重了，这个铭记于心，我们会把组织纪律规章制度铭记于心。但是我们把其他的学生的需求铭记于心，总是觉得有点过头了，所以你就直接说，别忘了其他学生也需要借阅，学习的其他学生也有机会的需求，这样就可以了。所以这里可以把这个肯定句转换成否定句，就能起到这样的效果。接下来在未来还是同样的问题。如果你一说未来就让人觉得还挺遥远的，如果说今后这个会好一点，就就是不久的未来马上就要开始实行逾期归还图书的惩罚将被严格执行，中文中没有这么长的定语，如果说这个惩罚将被严格执行，这个长度倒也可以接受，但是如果前面加这么长的定语，逾期还书的惩罚，这个就非常不像中文的结构了。另外这个惩罚被严格执行，这个搭配也是存在问题的。所以这里首先这句话太长了，必须把它断开，那就是说今后将严格执行借书的规则，然后再断开。凡逾期不还者，将照章处罚，按照要求进行处罚，这样会好一点，更符合中文的语法习惯。

三号同学整体比较连贯，也不是特别的啰嗦。当然了也有一些地方，这个还不是特别的地道，尤其是对被动语态的处理，比如说学生们被要求记牢借书和还书的规则，学生们被要求记牢。这其实不是很符合中文的表达习惯。如果是图书馆的告示，贴在门口的话，还是不是这个不太容易让人一眼就看出来这句话是什么意思，不太符合这种告示这种语体的需求。最后一句有一个这种现象，就是他喜欢词语叠加，严格的加强执行借书逾期归还的惩罚，如果你说严格执行，其实就可以了，就不必说严格的加强执行，加强这个词是和什么乘法搭配的，加强什么什么的惩罚，听起来还可以，但你这些词全部都堆积在一起，就会觉得非常累赘。

四号同学应该是把这小段这一小段话全读了之后才开始动笔翻的。四号同学有一个词，他是不知道什么意思，这个overdue他不知道是过期的，他看到这个over，想到可能是过了，就看到这个词缀，词根这种记忆法觉得可能是过了什么，但是他他这个意思有点偏差，他翻译为过度。第一句话它翻译为在图书馆过度接触的现象大幅减少，引起了人们的关注。前半句就非常累赘，在图书馆过度借书的现象大幅减少，还挺让人费解的。接下来还是每个同学都会犯的一个毛病，学生们被要求遵守什么什么的规定，被要求那是被谁要求呢？所以学生们他们的语言水平是不错的，但是他们不太了解这个有一些固定的文体的写法，就像这种告示的写法，现提醒现要求大家注意什么什么。这种现这个词，这一个词就能把这个被动的感觉给表达出来。这个时候还有一个问题，就是有点啰嗦，并记住其他同学也会需要借书的事实。这个事实这两个字完全可以删掉，要记住其他同学也需要借书，这样就可以了。最后一句，在未来这个词刚已经强调过，不是很喜欢这个词，今后或者说将来都可以。对于过度借书的惩戒将严格得到执行，非常的拗口，将严格得到执行，你就说将被严格执行就可以了。这个另外惩戒将被执行，好像应该是惩罚，执行某种惩罚，惩戒好像不是这样搭配的。

五号同学注意到这个第一句的时态，加上现在这两个字表明这个事情发生时间。五号同学另外一个处理的比较好的地方，就是他没有翻译出students are asked 这个被动时态，它翻译的是学生们都应当将什么什么的规则铭记于心。然后下面说，并且也知道还有其他同学需要，那这半句就有点问题，需要什么，并且知道还有其他学生需要，你可以说有借书需求，这样会好很多。最后一句，在将来逾期未还书的行为将被将被严厉惩戒，将受到严厉的惩罚。惩戒这个词总觉得在这里不是搭配的不是特别地道。

六号同学第一句翻的相当不错。把这个令人担忧的是放在了句首，显得整个句子很地道，而前面五号同学就没有翻译出令人担忧concern 这个词是没有翻译出来的。第二句，学生被要求提醒自己什么什么的规则，还是这个问题。被动语态最好是不要翻译出来，但是他后半句翻得很好，并考虑其他学生的需求，语气还有信息都处理的很到位。最后一句有点这个逻辑上有点问题，搭配有点问题，未来对逾期未还的书本将更加严格的实施惩罚措施，那书本怎么实施惩罚措施呢？你要惩罚的这个对象应该是人，而不应该是书本，所以这最后这一句话要在重新的考虑一下措辞，虽然意思都到位了，但是措辞有问题依然是一个硬伤。

七号同学问题较多，比如说第一句他说有人担心的指出担心的指出这个搭配是有点问题的，可以说对什么事情有些担心，但不太会说担心的指出，你可以说担忧的指出，还有第二句，学生们被要求提醒自己借书还书的规则被要求提醒自己听起来非常的拗口，首先中文不太会用这样的被动句式，另外提醒自己就起不到这个告示这种语用的目的。然后后面半句并关注谨记其他同学的需要关注谨记这两个取一个就可以了，所以建议把关注删掉，有点啰嗦。然后最后一句这个意思发生了明显偏差。其实是以后对于逾期未还图书的这种行为，要适应要进行处罚，这是第一个点。这个意思在要在第二个是这个惩罚会要被严格的执行，他这个最后一句就意思就偏差了，他认为是这个行为要严，要禁止这种行为，其实跟原文的意思相差甚远。还有他的搭配有问题，以后过期未还书目的行为会被严厉禁止，严厉禁止行为，这个是很不搭的，还有过期未还书目的行为听起来相当啰嗦，就是逾期未还就可以了。

八号同学错的比较离谱，尤其最后一句话。先说到前面要注意的是，图书馆储书已经大幅减少，我们可以说图书馆图书库存或者存书的量，但我不会说储书图书馆储书大幅减少，非常费解，这个不是很搭。第二句，学生被要求记得借书还书的规则被要求记得那多拗口，就直接说，学生们要记得是什么规则就可以了。记得参与的需要什么呢？你可以说他人也有借书需求，这样是不是更加的明确一些。毕竟中文他是不害怕重复的，它不像英文。最后一句话，这个意思就完全完全跑偏了，这个学生肯定是不了解overdue的意思。overdue不是超量，这里这个over不是说是超过这个数量，他其实过这个超期的意思，所以这个词的理解错误，导致这个学生最后一句就没翻出来。规范将被严格执行，那这里严格执行的并不是规范，而是一种处罚。什么样的处罚呢？借书这个借书超逾期不还这种处罚。所以最后一句话在中文中必须是要分成两句说的，否则就会显得定语过长。

九号同学翻的整体上不错。但第一句，曾有人担忧的指出什么什么惊人的减少了，这里我觉得这个曾有人担忧的指出，比较具有文学色彩。对于原文的这个语体register不是特别符合。第二句话还是一个被动语态的问题，十个学生中，到现在为止，只有一个学生没有翻译出被动语态，其他的都把这个被动语态翻译出来。而中文是不是很喜欢用被动语态的。学生们被要求提醒自己，听起来非常的别扭，其实就是学生们要提醒自己，这样是不是要好很多。然后后面这半句并且铭记其他同学的需求，铭记我觉得总体总觉得铭记这个词有点是虽然是非常书面的一个词，但是这个词程度有点太过了，就是记住就可以了，不一定要说铭记铭记，有一种刻在心上的感觉，但也不必到如此的程度。

十号同学第一句被特别关注到的是这里并不是特别关注，而是说令人担忧的是，他并不是让人去pay special attention，就是感觉和concern 是有一些出入的。另外书库存这个图书馆中书库存说法这个说法非常奇怪，也我们也不太会这么去说。还有第一句这种结构是非常的欧式中文，也不太会这么说，你看被关注到的是图书馆中书库存的急剧减少，中文似乎没有这样的结构。下面这一句还是这个被动的问题，中文不太喜欢用被动学生们被要求，就是说学生们要时刻提醒自己就可以了。那么最后一句借书逾期不还在以后将被严格督促。首先督促这个词就完全翻错了，它不是督促，是借期逾期不还的这种行为的处罚要被严格的执行。他这里你被严格执行处罚严格执行这种处罚，这个点就没有翻译出来。他仅仅翻译出来书不还这个词，而且他也没有提到这个借书不还以后要有一种处罚。这个处罚这个词也没有翻译出来，属于明显漏译了。

E4：

译文1总体来说，把意思翻译出来，但是还有很多细节问题，比如说第一个裸婚这个词，他先翻的是free marriage，然后自己改成了naked marriage，naked marriage在西方有自己原本的意思，并不是指裸婚在中文里面的意思，所以呢，也不太合适，他可以把他加个引号，然后后面注解都是ok的，但这里没有，所以要扣分。然后后面的没车没房，一系列没有什么什么东西是否定并列，所以不能够用and，而应该用or，对这个地方也要扣分。然后呢，他后面的年轻人生活压力太大，而且爱情的独立等等后面的整句话，前面是原因，后面是结果，他本来翻译出来的一个so，但是so并不合适，因为是笔译，并不是口译，他可以在前面加个given，然后后面就会把那个so去掉就行。而且总体来说比较罗嗦...这一段话这一句话，总体来说，翻的基本合格，但是没有达到优秀的标准。

译文2总体来说翻译的比较简练，但是也有一些逻辑还有细节的问题，首先他的裸婚这个词翻成了free marriage，完全是错误的，所以这个术语就要扣分吧。然后后面的which refers to比较好，它翻译成了一个从句解释前面的这个现象，然后同样的问题就是否定它用成了and，而应该用or。**然后面年轻人压力生活大，且强调爱情的独立，他这个地方翻成了独立的一个句子，其实并没有把它原本的因果逻辑关系给翻译出来，所以逻辑上就要扣分**。而且他把强调爱情独立suggest the independence of love，这个地方也是有问题的，读者读不懂什么叫suggest，强调爱情中的强调翻成suggest是不合适的，所以也要扣分。然后后面呢，他说，大肆操办等等，现在变得逐渐削弱这句，总体还是可以，但是放的有一点点太过于字面，可以稍微转一转，这个试题1也比较类似，都是把意思翻译出来，但是感觉逻辑都没有得到体现，所以只能够达到合格，也不能达到优秀。

译文3翻得总体来说比前面两个要好一点，但是有很多的语法错误，前面的他说裸婚翻译成为naked marriage，然后打了一个引号，其实像这样是可以的，就想读者知道，并不是原来字面上的意思，但是后面缺了一个解释，可以增译一下，这个地方暂不扣分吧，因为他的一个引号也说明了问题。然后后面的他否定并列，用了or，但是or用错了地方，我应该放在最后一个，就是在不度蜜月前面，他放在了没有车那个地方，所以要扣分，语法错了。然后年轻人生活压力太大了，说young people have great pressure，这个地方ok吧，生活没翻出来，要扣分，然后强调爱情独立，emphasize on the independence这个地方语法错了，要扣分，然后后面的therefore the tradition这个地方，他其实也把整个句子的因果关系重点放在therefore来体现上面，但是therefore一般是单独做一个句子的，不应该放在句中，所以这个地方也不太合适，要扣分。然后最后一句他翻的特别字面，就是翻成了什么has decreased its influence on young people就很Chinglish，这个地方也要扣分，总体来说也是一个合格的水平。

译文4总体来说比前面三份都要差，问题特别多，首先裸婚他翻成先simple marriage，一个引号算是ok吧，暂时不扣分，然后后面的他说了否定的并列，他用的是or这个还是还是可以的，但是用了太多的or，用or放到最后一个就行了，蜜月翻成了honeymonth完全错误，然后他这个句子翻得很奇怪，什么only being proved by marriage这什么意思啊，他可能不知道那个结婚证怎么翻，所以这个地方翻得比较奇怪。还有后面强调生活压力大，他说的是social life，而原本只是说生活并没有说到社交生活，所以他增意了也要扣分。Pay attention to independence of love也算可以，然后最后一句他说什么什么traditions dying，而原本只是削弱，并没有说消亡，所以这个地方也是有问题的，望文生义了，所以有点过分延伸，应该要扣分，总体来说这个不能够及格，问题有点点太多了，语法错误有，误译有，增意也有，都是问题特别大的。

译文5总体来说和前面3份有一点点类似，有些小问题。首先裸婚，他翻译了marriage with nothing 也有点太过了，并不是nothing，可能他有点点过于延伸，然后后面的没有车没有房，他用的是and不太合适，然后后面的年轻生活压力大，他翻成的是have large stress形容词误用，然后他说underline the independence，underline这个地方用的很奇怪，不应该说人是underline什么stress，然后他这个句子翻译的还行，因为他把那个因果关系用一个as表达出来，整个句子就联系起来了，所以逻辑还是可以的。但是后面的就是整个句子有很多的词用的不太恰当，这是他最大的问题。

译文6看得出来学生的语法结构问题特别大，问题如下：第一，她说裸婚他翻成了bear marriage错了吧，他可能原来是想要写那个bare，这地方完全错误。然后后面的没车没房他并列也用上了and,错误，然后后面honey moon也分开写了，也是错的，然后整个句子很奇怪，前面用了从句，后面又突然来了they，就感觉断开了，结构错误。然后最后一个也是同样的问题，就是说什么为with high stress，然后或什么什么什么的，tradition is faded away这个地方错了，要么是is fading away要么说faded away，所以也是语法错误，所以总体来说这个最大的问题不在于意思而在于它的语法结构还不太成熟。

译文7这位考生翻得特别粗糙，有点敷衍，首先裸婚这个词也翻错了，naking marriage没有太用心。后面的就没有一个正确的句子，语法都是错误的，referring后面又有突然来一个动词，就是明显的就没有太用心去翻译这个句子，所以结构完全混乱。同样也有一个并列词用的and的问题，所以还是要扣很多的分。然后后面再接着看，in modern life ...has a great living pressure，勉强过关吧，indicate the independence of love中indicate和suggest是这样的问题，不懂是什么意思。他要是强调的是强调爱情独立，并不是说什么暗示指示，所以是用词错误。然后最后的tradition......luxurious这个词用的太过了吧？可能还想找一个比较fancy的词，但是找的一个词并不合适，而且他这个句子的结构也是完全混乱，逻辑没有很好的体现出来。

译文8首先看他的裸婚这个词，问题是一样的，naked marriage并没有加引号，所以会误导读者，然后后面呢refer to a couple get....，这个句子又是一个是语法错误，这个语法错误and也没有用成or，然后后面的他把句子都分开写，就是把三个句子，年轻人生活压力大一个句号，然后爱情独立一个句号，然后就是什么什么削弱的又一个句号，完全分散开了，就没有把逻辑很好的体现出来，逻辑上就要扣分，然后面那个生活压力大，他这个什么啊，算是勉强过关吧，然后后面什么爱情的强调性独立翻成了declare，是要宣布独立吗？这个太完全错误了。然后大肆操办holding a great ceremony for marriage....可以的。It’s necessary这个地方结构完全错误了，而且他也没有把那个结婚证给翻出来。

译文9还有很多和前面类似的问题，第一个裸婚这个词，他翻的是marriage without other things解释的太过了，他可以先把那个词给直译出来，然后再注释都可以，就是后面加一点点，这样直接翻译出来反而显得就没有任何意思，所以这个不合适。然后没车没房也成了用的是and换成or更好。然后后面only has a marriage certificate这个地方一个动词，然后前面和refer to构成了一个结构上的错误，语法错误和前面是一样的，就是这样的问题就是语法上如果句子太长就会出现错误。然后后面的他也是把三个句子都翻成了单独的句子，本来是由逻辑关系，并没有体现出来，也是一个前面一样的问题，young people living in modern times are very stressful，stressful并没有把这个生活压力中的生活翻出来，所以也是不合适。underline用的非常奇怪，他可能不知道，另外能够表示强调的词吧，underline不能够用到这个地方。

最后一个是相对来说较差，就是第十份，首先他裸婚这个词，原来是想说naked marriage，这个词写的，单词拼错了。Is a new way of marrying这个地方就奇奇怪怪的他可能是故意强调ing，marriage不就行了吗？Which means a couple...wedding holiday，蜜月也没有翻出来，wedding holiday是什么？然后emphasize the independence of love这个地方翻得很好，好纠正一下，这个人，他翻的比前面大多数要稍微好一点点。

E5：

我们首先来可以看一下这个文章，应该是属于一个叙述性的，或者说是描述性故事。他也非常符合我们对中文的定义，是流水句，是意合。英文也就是我们所说的形合，它通过语法的一个逻辑关系来串联起来非常的紧凑。学生在翻译的过程中，我们其实可以看到这个学生的译文其实首先在信达雅的“信”上，就是在信息的完整度上，其实是已经做到位了，也没有太大的语法问题，所以说明学生他的基础还是比较扎实的。但是我们可以看到，比如说刚刚当说到首先的话，学生第一个想到的就是firstly，这其实就有点类似于“假朋友”的感觉。学生可能平时接触到议论文写作比较多，他看到首先就想到了一个firstly, 但其实这里我们按照原文里面的first一开始，或者at the beginning，用这样的表达其实是更加符合这个译文的要求。其次我们可以看到这架飞机从波士顿飞往洛杉矶，学生用的是非常简洁的一个from...to。如果他平时可能坐动车有留意的话，其实我们会比较说this train is bound for，那么它的目的地是哪里，所以说明他有语言的基础，也比较扎实，但是可能在高级一些的词汇，他也不太了解，还比如说突然掉头南下冲向纽约市中心，这也其实也是说明了学生再次说明了学生他有语言基础，但是我们学生对于英语可能还说他在平时学习过程中可能只是背词条而已，背的就是说中文英文意思的对照，他没有去翻阅过英字典，或者说在翻字典的时候没有去看英语中的一个详细的介绍，以及他对于词汇的搭配还是比较不了解。就是了解词汇了解的还是比较表面，只知道意思，但是不知道如何搭配，如何去使用。就比如说句子里出现了一个attention，那么attention 的搭配我们一般会用get，catch，grab , draw attract. 但是学生用的是seize，seize这个单词其实也是比较高级的一个单词。但是在这里却出现了一个误用的情况。其次的话，英语的译文其实还是比较好的，非常符合英语一个形合的概念。首先它用的是强调句，it was said, 然后把前面两句中文的句号的中文的句子给串到了一起，之后飞向向西飞行，飞过阿迪朗达克山。那它这里的飞过，它并没有用一个动词来表示出来，而是直接用了一个over。那其实是非常形象的。其次它的这个结构，它不是按照其它学生一样，把它按照一个时间顺序，一个小句的译出来，它是把后面几个整理成了一个句子。It headed over before. 其实可以看出他的一个逻辑是非常清楚的。看了一下就能够一目了然。学生在这一方面，他就是按照小句来进行翻译，他的信息是到位的，但是可能就是说没有英语那么地道。

第二个学生，我们来看一下他的译文，我们可以看到他的译文没有太大的语法问题，所以说明他的一个语法还是比较扎实的。而且他也在第一个里面，他使用了一个主语从句。What...is a 767 plane 用了一个主语从句，所以说明他还是有一些语法基础在这里知道要句式多样一些。首先他也跟第一个学生一样存在的一个问题就是firstly 我们讲到firstly 其实是在议论文里面表示论点。第一点第二点第三点第四点我们就会用firstly 。在这里学生也是误用了，说明跟第一个学生一样，他只是知道中文跟英文的一个对照的意思。但是可能没有去深入的去了解这个词汇。第二个出现的一个是误译的话，是引起安德鲁注意，在这里把它翻译成了一个interest。interest 我们在初高中的时候，其实就已经学学过是兴趣。这个错误我觉得还是一个比较低级的一个错误。大家应该碰到这个注意都会想到attention 或者说notice。其次我们在这里可以看到波音767型飞机。在这里他学生把波音省去了。其实说明他可能在平时生活中并没有太多的去了解社会上的一些新闻。如果他其实有去了解过，平时比如说我们前几年出现了一个波音飞机的事故，其实我是知道这个波音应该是怎么样去拼写的。这一点第一个学生的话可能就相对来说知识面更广博一点。再次的话我们可以看到它会使知道要是飞往洛杉矶，他知道要用bound 这个单词，但是他在使用的过程中也是搭配存在的问题。

他用的是bound to，那么bound to 我们平时可能会说be bound for。就是这个事情注定要失败。但是如果我们在表示这个飞机可能要飞机或者说朝什么方向，我们肯定是用be bound for。其次再出现一个误译的情况的话，就是说这个学生，他比如说他用的是一个飞机起飞，他用的是take on。其实飞机起飞是应该用是take off。这个take off 的一个词组应该也是高中的一个基础词组。所以说明学生在这里还是存在着误用。其次是flew to west 。其实west 前面应该是要加一个the flew to the west . 因为我们可以想到西游记就是a journey to the west，所以他前面其实还是少了一个限定词的。之后我们可以看到flew ，我们可以看到它翻到了，突然掉头turn southward ，向南掉头。但是他学生在翻译的过程中，其实漏译了冲向纽约市中心。我们从这里我们就已经看到，学生在这里已经大多数的信息已经体体现出来。但是他漏译了波音，同时也漏译掉了一个冲向纽约市中心。这个来说，对我来说其实是一个比较重大的一个信息的漏译。其次的话它也存在着一些一些语法的问题。比如说interest 搭配有问题，take off 词组搭配使用有问题be bound to也是搭配搭配有问题。然后flew to west ，这里是少了一个the。我们可以看到这个学生在信息量方面可能是做到了85%。在语法方面，他其实可能还有较大的一个提升的空间。从第一个学生来还是比较不扎实的，所以他的分数可能在打的时候可能会打80分左右。

第三个学生，我们首先来看一下他的一个信息量，他的信息量全部都是到位了的。所以他在信息方面是没有问题的。其次我们在回顾到词汇方面也是他的一个首先的问题。他也是使用了firstly 属于一个误用的状态。之后，他的词汇还有一个有问题的话是波音767型飞机，它用的是aircraft，而不是用plane。我们其实这也是一个初高中的一个知识点，aircraft 它是不可数的plane 才是可数的。所以这里这是一个词汇和语法共同误用的一个状况。更多的话可能还是一个词汇的一个角度。接着是飞机飞向它，在这里可以使用headed，也能够说明他平时是有积累的。但是在这里又存在一个搭配的错误，一般就是headed for 跟bound一样，bound for headed for. 我们在这里是搭配存在问题词汇方面的话就没有什么问题了。就是三个，一个是firstly，一个是aircraft ，还有一个是headed for 。我们从整体意思上刚才已经讲过了，意思上基本上都已经信息都是到位的。所以信息方面没有什么要扣分的。再从句式和一个结构方面来讲，它首先能够使用一个主语从句，然后也懂得把句子几个句子整合在一起。但是有一个地方比较冗余的就是说这个飞机从波士顿飞往洛杉矶，载着81名乘客。飞机上午7点59起飞。他其实一个是讲了在翻译的时候，一个是翻了从波士顿起飞，然后又又讲了一遍，在7点59起飞。其实它可以把它们整合到一起，变成一个句子，这样的话可能会让结构更加的紧凑，而不至于显得有些冗余。整体来说，句子的一个句式也是比较多样的，就不会比较单一。所以也是说明他的一个基础还是比较扎实。

第四位学生首先也是一个问题，firstly 使用错误。第二个是波音767 ，它的波音没有翻出来。其次是他的一个fly flew flow，它的一个fly 过去式写错了。这是一个非常基础的错误。在初高中就应该已经学会了一个动词的不规则变化。那么在这里出现这个错误其实是非常基础的一个错误。翻错很可惜。再者的话，他的一个信息量其实基本上都到了。再从语法上来的话，就是它的一个句式就非常的一个单一。第一个的话还是跟译文一样，有一些抢眼，用了一个强调句，我们也可以看到它的Andrew也是拼错了，也是一个非常基础的错误。我们就想到词汇有这四个错误的。Firstly，波音， fly 的过去式，还有Andrew 拼四个错误。整体意思的长基本上都已经点到，但是存在着一处严重的漏译，就是掉头南下冲向纽约市中心，没有翻出来。信息确实不够完整。然后从整体的一个句式结构，首先它能够使用一个强调句有一定基础。接着但是它的一个句子还是有点像原文的句子来说，不怎么知道把句子可以整合在一起，所以说还是像中文的一个流水句，缺少点英文的这种语法衔接不够的一个自然不够的一个紧凑。然后还有一个语法的问题，就是flying over这个地方，句子主语是有问题...有些奇怪，他的一个前面都是took off flying towards，然后后面可能是想要跟句子多变一点，多变一点。类似于说知道它一个主语是前面的plane，然后它在这里把它表示flying over。但是其实放在这里有点奇怪。

第五个学生的话，他的first 使用是对的，他把它变成了一个也是主语从句。the first thing that is a plane，然后他的一个词汇问题，一个是波音，波音不会写。其次的话是fly from Boston to LA，它是用的是flight 。flight 其实是一个名词形式，在这里应该用fly 而不是flight，所以存在这个误用。接着是 kept on flying to the west，这个keep 就是keep doing something， kept on doing, 应该也是一个误用的情况。然后接着是下面自创了一个past up there 搜了一下，并没有这个表达，所以它这也是自创的一个误用。接着是掉头南下，turn on south 应该是turns off，所以也是存在一个使用错误。所以说明他的一个在词汇方面就其实已经不是特别的一个扎实语法。从整体意思上的话，大致的意思都是在了。从语法上在最后一句，它使用基本上就是还原的原文的一个结构。用了流水句，但是流水句写到最后rush to the center of new York。前面其实少了一个and，就是英语的这个句子的一个语法衔接的一个逻辑缺少了，然后整体的句式也是比较的单一，没有太大的一个亮点。基本上就是把这个流水句堆在了一起。

第六个学生，我们首先可以看一下他整体的意思全部都已经是到位了。然后从他的一个句式的话，第一个首先用了what 主语从句，what caught Andrew’s eyes 比较的亮眼。第二个的话，他the plane flying to，然后再有81名乘客用了carrying 81 passengers，用的也是比较巧妙。可能大多数的同学都会想到用with，之后的话它的整体的句式其实还是偏向于中文的一个流水的句式，没有对句子的意思进行整合组重新组合，所以可能整个篇章来说会显得有一些些散。从词汇角度跟之前同学一样，首先firstly 翻错了，然后波音这个波音反正写错了，然后比较亮眼的一点，就是caught somebody's eyes也比较亮眼的一个表达。然后从语法角度的话出现了, took off on，然后加这个时间。这个具体的时间点应该用at。然后还有一个是turn around it direction? 这个it 应该改成its。所以这两个语法问题是比较基础的，问语法的问题应该是能够避免的。然后最后一个就是rush to 这用的不够贴切。因为它其实表示它会南下冲向纽约市中心。没有译文里面的dive down toward 更加的一个形象生动。所以的话。整体意思到了，但是结构方面要扣分。还有他的fly 的过去式也拼错了。

第七个学生的信息基本上都到位的，整体信息意思都到了。然后句式的话也是有点按照原文的句式来。但是第一个首先用了the one which 那个主语从句比较好。然后第二个的话就是后面用了一个catch 81 passengers ，所以其实应该是想要表示carrying ，但是用错了，所以有一些可惜。然后之后的话就是中文的话是把over翻成了飞过，但是它翻回去的时候也用了一个over，所以没有像中文一样一直用动词来连接，巧妙的用了一些介词，也体现了一个英文的一个形合的一个特点，也体现了英文静态的一个特点。语法上的话基本上没有什么问题。词汇的话基本他也是第一个把首先翻对的考生，波音拼写有些问题。caught one’s attention 在这里用的挺好的。

第八位学生的话大致意思都写到了。然后他在句式上基本上句式上基本上是遵循着原文的一个顺序。第一个一开始也想到了，可能想用一个主语从句，说明他有一定的想法。但是整篇语法问题存比较大。首先第一个是first thing，前面应该加the first thing 少一个限定，少了一个限定词the。然后Andrew 应该是一个非常简单的一个单词，基本上应该是一个非常基础的人名，所以他不会拼也比较神奇。第二个句子可以看到this plane from Boston to LA，也进行了一定的整合。之后的是flew straightly 这个straight 它不止它可能只记着这有直线作为形容词的意思，但是不知道straight 也可以做副词，所以在这里用了straightly 也是比较奇怪。然后掉头南下，直接掉头，其实应该是转换方向的意思。然后他被这个中文的原文的意思给困住了，不知道怎么翻译，把它变成了change is head down，所以就是比较奇怪，然后这个south南下南south 这个单词好像也拼错了，所以他这个还是存在着挺大的一点点语法问题的。

第九个同学全文基本上意思都到了，然后句式的话基本上按照原文的一个顺序没有进行太大的整合，所以流水句比较松散。第一个首先他能够想到用the first thing that caught Andrew’s attention was 它的一个句式，比较亮眼。但是涉及到词汇角度的问题，一个是词汇搭配不当，seize attention 应该用got caught ，或者用grab 等等一些单词。然后波音的话它是写不来采取的一个拼写。其实拼来拼写还是不写。之后，他一直向西飞行，这里也是他不知道straight 可以作为副词，所以把它写成straightly，把以为把它当做是一个形容词，把它变成了副词的形式。然后向西飞行这个toward 跟forward 没有搞清楚的意思，过于之后是过于紧跟原文的语序，飞跃什么什么的上空。他用了across above the air ，自己编了一个表达出来，其实这么冗余的一个信息，用一个over就可以表达出来了。然后语法问题的话这是一个，这个句子其实都是用 and 连接起来的。然后在这里忽然出现了一个changing the way 这个语法是有些问题的，冲向纽约市中心就把它冲翻成了going to，削减了原来的一个力度。

第十个学生整体意思也都到位了。然后我们首先它的句式的话，一开始也可以看到他把这些句子整合了一下，所以看起来更加的紧凑一些。然后从语法词汇角度，首先他用了一个主语从句，what called notice of Andrew is the Boeing is 767 . 然后这里一个是notice 使用错误了，一般不会吸引引起某人注意，一般不会caught notice of 这个表达。然后波音767波音拼写错误。此语法took off in 7：59，是不是应该用at, fly过去式flew使用错误，然后这个山fly over，他用的是across 怎么across ，这一个介词一般都是指从河的这一边到大河的另一边，或者从街的这边到街的那一边，或者说fly across the sky，反正在这里有一些些奇怪。然后他的词汇量可能crushing，这个说明他词汇量还是有一些词汇量的。但是这个crash 是挤压的意思，在这里翻译的话明显是不合适。

E6：

一号同学的译文整体上从内容上来说，他基本上是90%都处理出来了，然后可能会存在个别的一些误译，极少数的漏异现象。他的比如说第一段的翻译的话，我认为就是它比较大的一个问题，就是在于这个连接词的处理。就这第一句话里面它是有几个and的，但是它每一个and 都处理成了“并”。其实比如说我们相信全球变暖问题给我们带来了巨大的威胁，然后认为全世界应该通过行动来改善。这里其实不需要把这个and 翻出来的，因为中文当中是没有连接词的，只是英文当中有。然后最后一句这个地方的and 它也是把它翻成了并其实应该是说现在流行的那些理论推断，它是需要不断的被证实的。然后如果你收集到了更多的数据，那么它需要再进行测试，就说明这里的and 的话它其实是表示进一步的递进关系，而不是并列，所以他在连接词的处理上面的话，可能就是没有处理的很好。然后第二个句子的话就是会存在很严重的一个他没有脱离这个原语境，就是他不会把它进行深层次的加工。比如他每一个被这里学生被要求他们都是处于成了被动。这个问题的话，其他后面的同学也都有最严重的地方。就是在最后一句话，就是对于那些，逾期未归还书的学生，我们会进行严厉处罚。但是最后一句话，他是直接照着原句翻，就是说对于逾期不还的书，会严厉对学生处以罚款，其实这句话就是一个病句，因为处罚的是学生，他的对象就是说对于书。所以这个地方的话应该是明显的是一个原语的脱壳，可能没有处理好。第三句的话，他的问题就是在于他的难点其实也是在于什么没房没车，不办婚礼，不度蜜月这一句话的话很长又有很多个谓语，他就真的把每一个谓语都翻出来了，他其实可以借助一个把他们都处理成名词，这样就不会有多个谓语并列，还有包括他这一句话的重点，其实是说这个结婚方式是说恋人他们是怎么样结婚，但是他却把它处理成了裸婚，它意味着是一对夫妇。这里的重点其实有偏差，包括他这个裸婚这一个词的话，他就直接没有翻译了，我觉得这是非常不好的。就是其他同学有些他们也不知道，但是他会尽力去翻。但是空在这里的话就是完全不行。然后最后一句话的话，其实他的难点应该是在后面最后一句，他上午起飞向西飞行，飞过哪里掉头，然后再冲向他这里中文的话是只有一句话，但是他在英文的处理当中，他也是把它处理成了一句话。他在这里的话，其实他忽略到了考点应该是在于断句，所以他整个这样子处理下来的话，整句话就是会让人把握不住重点，而且很奇怪。然后包括它前面这个地方，767型飞机的话，它可能也是不会翻，所以直接是把它漏译掉了。总而言之，就是这一个同学他在内容的完成度上面是比较高。但是在每一个句型他的考点他每一个考点其实都没有达到考试者想要的。比如说第一句主要是考察他的这个and 的翻译。第二个句的话可能是这个被动的对象。第三句是多个动词的并列的处理，第四句的话应该是这个断句，就是他每一个考点的加分项，其实他都没有完成出来，所以他的翻译只能算是说很中规中矩的那一种，但是达不到朝上的水平。

二号同学的翻译很明显的一个问题，就是他的错译现象，还有漏译现象是非常明显的。就是他相比于其他同学的译文，首先在长度上面就短了很多，然后这是它很突出的一个问题。然后具体看的话，比如说第一句的话，它有一个很好的地方处理的很好，就是这个climate change is a certainty，他把它处理成了板上钉钉的事实，我觉得这个是我看了这么多篇处理的最好，因为其他同学可能都是翻成了什么确定性，但其实这个是不符合中文的用语的。但是他后面这个地方就犯错了。there are no certainties in in science , 它是指的是说科学无绝对或者无必然。但是这里的话他可能是理解出现了偏差。然后包括后面这一句话也是要验证这一假说，它并不是说验证气候变化这个假说，而是指现有所有流行的这些理论都需要用时用证据来进行测试的，所以他这个同学非常明显的，就是可能他是存在一个理解的偏差，从而导致错误现象很多。然后第二句的话，同样的他也是出现了这个学生被要求，其实一般中文的话很少用到被动，就直接是说要求学生，学生应该干什么就好了。只不过在英文当中会把它处理成被动，还有包括他最后一句，我们是说会对那些逾期未归还书的学生会进行严厉的惩罚。但是并不是说不准禁止这种延期归还图书，所以这也是一个理解的偏差，第三句的话就是很明显的，它出现了一个漏译现象。就是后面这里现代年轻人生活压力大，强调爱情的独立，然后什么什么之类的，他直接就翻成了live stressfully and and emphasize 之类的独立。他应该是不会写，那后面这个大肆操办，他也是把它省略掉了。那这一点的话，其实作为改卷的老师会特别在意这个信息的一个完整度。包括一个它的正确的一个理解，这个是非常重要的。然后最后一句的话就是很大的一个问题，就是从这架飞机一直到最后的话，他有一种是字对字的翻译，就是这架飞机从波士顿飞到了洛杉矶，有81名乘客，他就是字对字的把它翻出来了，就是完全没有进行加工，这个英文的话是非常的糟糕的。所以总结一下这个同学问题比较大的地方，一是内容的一个完整度漏译有，然后二是一个内容的诠释，正确理解错误现象也比较明显。第三个是内容的一个加工，它也没有对信息进行一个重新整合，而是直接把它翻译出来了。这是他比较明显的一个问题。

三号同学的翻译的话，其实他有很多的亮点。首先第一个的话是我觉得他在处理这种连接词方面的话就比第一个同学有很大的进步。比如说第一句话的两个and 他都没有把它翻成并列。第一个他知道就是把它处理成了因果。这个其实在语义上是说的通的。然后包括第二个and 是把它理解成了一个递进。我觉得他这一块是处理的很好。然后包括他在断句或者说调整语序方面也做的很好。比如说第二句的开头是it has been noted with concern，这是在前面的，但是它把它放在了后面。先说这样一个现象，它引起了大家的关注，我觉得这个是符合我们中文的一个表达，包括最后一句话也是说会对那些逾期未归还的书，我们会进行惩罚。他也不是说把他直接处理成被动，而是会把它补充主语翻成主动，包括这里也不是学生被要求，而是会翻成学生注意。所以从他的翻译来看的话，应该这个同学的中文功底应该会比较强。所以它把它英译汉部分的话，它的中文表达都是比较地道的，而且会对这些连接词或者是被动结构或者语序，它都会进行一定的调整。然后他的汉译英部分，其实也是得益于他的中文的这样一个断句。所以包括比如说英译汉部分的第四部分这里，飞机上午起飞，这一句话很复杂，他也学会了把它断句，而且我觉得断的也很好，就是把它处理成当它飞过的时候，它突然掉头转向冲向纽约中心，但是他在英译汉部分的话有一个问题就是把握不住这个英文句子，一个句子的一个重心，比如说第一个英译汉译英部分就是指一对恋人没房没车，不办婚礼，不度蜜月，只领取结婚证。那其实应该先说到是，which means the couple gets married , which means the couple only apply for the marriage certificate without a house car 或者什么之类的，应该要先把领取结婚证说出来，然后再说后面的东西。还有包括第二句的这个飞机飞往哪里有81名乘客，那应该要先把飞说出来，再把81名乘客with eighteen one passenger 放在后面，所以他在英汉译英部分的话，就是把握不住一个句子的一个重心。就一般我们先会说重点那次要部分，再往后去做。那他这一点可能是他的一个问题。然后汉译英部分的话，我觉得处理的是很好的。然后只有一个问题，就是那个certainty，就是这个确定性的问题。其实这个表达的话，一般来说我们中文也不会这样说。不过这个问题的话是所有同学都有的一个问题。总而言之，他的翻译还是比较好的。

四号同学的英译汉部分和之前的同学一样，就是在语言的一个深层次的理解和加工的话，是存在一定的问题的。比如说第一句的这个先前的理论肯定要不断的接受来自证据的挑战。这句话的话在中文上都读不通，然后包括后面的，而且越多的事实会使理论被重复实现。就是完全是照着原来的句型翻，但是不考虑中文表达是否地道。包括第二句，这里学生们是被要求记住图书借还规则，然后，什么逾期书的归还将在以后受到严格的管理。这些表达的话是很明显的不地道的，就可以看出来他在这个句子其实是没有完全理解透。其次，即便他理解透了，他也不知道要是可以把他用我们地道的中文表达出来。所以这说明这个同学在英译汉的部分，首先他的理解这一关没有过。其次他的表达也存在一定的问题。然后说到他的汉译英部分的话，就是第一句的话就是存在一个漏译是很明显的了，后面首先裸婚没有翻出来，然后其次最后大肆操办婚事，这个他是没有意的。其实这里是能够体现出来他的一个转换能力不行，应该是大肆操办，不会说。所以他就直接把它漏译掉了。然后后面第二句的话，同样的问题也是在最后一句这个多个谓语的处理的问题。它的话不是全部都把它们处理成谓语动词，它是把其中向西，然后飞过，还有包括最后那个冲向都处理成了非谓语。但其实这样子的话，他并没有准确的把握出到底哪一个词应该是重点，哪一个动词应该是处理成非谓语，它这个应该是想当然的就把它处理成了非谓语。这个地方的话建议还是应该要把它断句会比较好。如果是一定要这样子合成一句话的话，那我觉得应该是向西跟飞过是可以借助介词的。然后最后冲向纽约中心的话，这里可以处理成非谓语作结果状语。他的这个掉头的话，动词又用错了，包括整个的时态也是有问题的。

五号同学他的英译汉部分的话，第一个部分的话比较大的问题应该是在单词的一个理解。第一个，首先avert是避免不是预警，包括第二个test平常可能是测试，但是这里的话应该是要取它的引申义检验。它英译汉部分的话，第一个是一个词义的一个正确理解的问题。而且关键是这两个词的话都是一整句话的一个重点。如果把这个词义理解错了或者有偏差的话，那语义的传达是会受损的。他的第二句英译汉的话就是很明显是跟前面的同学是一样的。就是他在处理这个被动的地方，还有包括最后一句话，他应该也是没有完全理解，所以导致他翻出来的这个中文的话是不太地道的。什么学生被要求牢记，包括这里，什么关于期末归还书的惩罚将会严格执行。这里的话都是不是符合我们中文文的表达，包括他还有一个地方就是没有把他的隐含义处理出来。这里bear in mind the needs of the students 其实就是指的是说我们要谨记借书还书的规则，同时也要记住其他的学生，他们也有借书的需求，就是这个需求是什么，一定要指出来。那他这里的话是没有谈到的。好的，然后汉译英部分的话，其实他要比他的英译汉部分要好。汉译英部分的话他比较大的一个问题。第一句话就是前面说过中文的话，一般没有连接词，但是英文的话它是有这样一种内在的逻辑的。比如说最后一句话，他应该是存在一个因果的关系。就是因为他们现在生活压力大，强调爱情的独立，所以现在的这个大肆操办婚事的传统逐渐削弱，就这个因果的话他是没有体现出来的。他直接就把这两个句子并列放在了这里，另外要提的是他的汉译英部分的话是有很多长句，英文当中使用长句没有问题，但是它容易出错。比如说这一句没房没车不办婚礼，这里it means a couple getting married without car，这里倒是没有问题。但是后面but only 接待后面的话，这一句话就是不对的。这里建议他先把重点说出来，应该是which means a couple marries by applying for a marriage certificate without 什么什么，所以这句话是有问题的。然后下面这一句的话就是它的一个时态。很多同学其实在做翻译的时候，也会忽略这个时态。前面用的是现在时，后面又用的过去时这个时态不统一的话，也是一个致命的问题的。但他处理的比较好的地方就是这个后面的断句是处理的比较好的。这是这个同学的问题，就是英译汉部分的话可能在被动这一块，还有包括个别词关键词的一个理解跟传达，还是存在一定的问题。汉译英部分的话就是需要把这些句子之间的这个逻辑是需要在英文当中需要显化，它在这里没有显化出来。第二个就是句子太多长句了，然后就是容易出错，这是他的问题。

我觉得六号同学的翻译的话是我比较喜欢的一个。首先他的英译汉部分的就是第一个句子的话，它的那个并列词and 第一个它是没有翻。然后第二个把它处理成了随着，我觉得他这里处理的是非常好的，不过唯一的瑕疵就是可能在这个take steps 采取措施，它翻成了贡献，就这个地方的话，可能是词义还是有一点点受损的。然后第二句的话，英译汉的话问题是跟前面的同学一样的，这个是他翻的比较差的地方。其实其他三句都翻的很好。第二句也是同样的被动的表达，包括最后一句话，我觉得可能他们没有理解这句话，就是他翻出来的中文也很奇怪。逾期不还书的惩罚措施在将来会被严格执行，这句话其实也是不是中文的表达，应该是针对逾期不还书的同学，未来我们会执行严格的惩罚措施，还有包括他考虑其他同学的需求，什么样的需求也是没有处理出来的。然后这里的话要特别表扬他的是他的这个汉译英部分，我觉得处理的非常的好。首先裸婚，他不知道这一个词是什么，它就是相当于对它进行了意译，getting married with something, 我觉得算是勉强翻的比较好的了。包括后面这一整句话，就是说他们没有车，没有房之类的这个得分点，他也其实也是踩到了的。还有包括后面这一句就是必须大肆操办，这里他用了一个定语从句，其实他就是表示一个因果，我觉得这一个也很好。唯一不足的地方就是在个别词的一个翻译。就比如说这个生活压力大，其实就是bearing more life pressure 就好了，就没有必要说把它翻成more stress of living。

这句话就是有点点累赘，包括后面那个削弱他用的banish。banish 本身是驱逐的意思。这个词义的话还是有一点点出入的，特别要提到的是他翻的很好的，就是这个大肆操办婚事，把它处理成了a big wedding ceremony is a must 我觉得这个表达用的非常的好，非常的地道，这应该是他的一个加分项。他最后一句话的翻译的话，我觉得处理的也很好，他最后的话也是跟前面有一个同学有点点像这几个动词，它确实也全是翻成了一句话。只不过有一些词它把它处理成了谓语动词，有一些处理成了非谓语动词。这样子的话就是能够明确哪个是主要的，哪个是次要的。但是个人建议还是把它拆开来会更好一点点。因为这样子这个句子太长了，还是会有一点点把握不住重点。总而言之，这个翻译我还是比较满意的。

七号同学的话，他是英译汉部分，要比汉译英部分要好很多。他英译汉的话存在的问题的话，第一个句子应该是prevailing是流行的，他应该是把这个词看成了predict，翻成了预测，就这一个单词它可能存在了错译。第二句的话，我觉得是目前看来所有同学当中处理的比较好的。首先应该注意的，前面开头他又把它翻出来，然后包括这里学生，他也不是被要求他处理成了学生需要铭记。然后这个就是他唯一不好就是在最后一句，在未来应该是我们会严格执行。对于这种逾期还书行为的一个成绩。这里的话还是把这个严格执行放在了后面，我觉得要放起来会更好一点。他英译汉问题不大，主要问题就是在于汉译英了。汉译英的话第一句的话首先是它一些词的翻译，裸婚翻成bear marriage肯定不对，其次，这个领结婚证证书写错了，然后包括这个没车，车的话，其实就是car，他用了一个vehicle ，那是机动车，并且拼错了。还有新的一代应该是generation ，他这个是generous，就很多个别的词的翻译都不是很精准。然后包括这个冠词的使用，也是应该是without a house，a vehicle for a ceremony or honeymoon. 还有包括这个生活压力的话，就是heavy life pressure。他就是小的这个细节错误比较多的第一句。但是整个句子的结构还是在的，包括他后面是先把这个逐渐削弱说出来。然后再说是因为他们生活压力大，我觉得这个调序没有任何的问题，并且把这个逻辑显化了，我觉得处理的很好。他问题最大的就是在第二句的汉译英部分，我觉得他应该是看到这一句话，可能这个学生本身可能就觉得这个很难，所以他基本上完全就已经放弃了，因为真的有很多地方处理的都不对。比如说首先引起注意的，这里什么the first that 那它的整句话是the first was the 767. 这句话主干就不对，还有包括这里的，人家载有81名乘客，这里又用了一个定语从句，这个定语从句用在这里的话也是完全不对的。还有包括后面一些个别词的翻译，比如说这个pay的过去式，还有fly 的过去式，还有这里起飞，然后朝西走向西飞，他也是took the west direction。总而言之，他这一句话基本上的话就是勉勉强强的是说把一些词翻出来了，但是句子的主干都不在，那他的第一句的这个汉译英的话是句子的主干都还是在的，只是一些个别词的翻译。所以这个同学他问题比较大的地方，应该是在汉译英部分，他的英译汉的话是没有什么问题的。除了极少数的一个问题就是了。

八号同学他主要的问题的话应该是在单词这个用词这个方面。首先英汉的话是单词的个别词的一个理解。第一个step的话是措施，它这里就分成了一步步。然后包括这里科学没有绝对的，这里是no certainties，但是他自己又多补充了一个解释，这个的话是他自己增意部分，然后也不符合原文上下文的。还有包括这个prevailing，它是流行的，它又处理成了令人说服的。就它这些词的翻译的话，可能是记错了。然后第二个部分的英译汉还是一样，跟其他同学一样的问题，就是这个被动了，我就不详细说了。然后说一下他的汉译英部分，他的汉译英部分，首先裸婚，他翻的simple wedding，那也算勉强翻出来了。那后面这里就是他这一句话问题是比较大的，这个没房没车，不办婚礼，不度蜜月，他把它拆成了两句话。那如果要拆也没有关系，但是后面这个地方就有多个谓语在一块了。比如说they get married without wedding ceremony，and honeymoon only get marriage certification，那这里证书又错了。其实这句应该是 only get their marriage certificate without a holding wedding ceremony or a honeymoon，我们这里的话，他这句话就是多个谓语在一块，这就是一个病句了。**还有包括后面这个用词，也是婚事的话，用的是affair， affairs 的话，它是表示暧昧关系的那种，用词很不准确**。包括第四句话也是一样的，他跟其他同学一样出现的是一个时态混用。前面用现在后面运用过去式，还有包括他的断句，我觉得是问题比较大。其实这个从哪里飞往哪里，跟载着乘客，他应该是在一起的，但是他把他拆开来了，还有后面这个地方。最后一句话也是他飞过哪里，然后又朝下冲向其实他这里有三个动词，但是他在前两个动词之间加了连接词，最后一个反而没有加，这是不符合英语的表达。其实他完全可以把最后一个动词处理成非谓语，然后变成了一个结果状语。所以这个同学最大的问题就是不论是英译汉还是汉译英，就是在一个词义的一个理解，包括词义的一个准确的使用是比较突出的问题。另外的话就是这个断句问题。

九号同学的英译汉第一句存在的一个很大问题是其他同学没有的，就是他有一fabricate的感觉，就是他没有完全理解这个句子的意思，然后又在自己的理解的基础上又增添了很多很多的信息。比如说首先这里我们不相信这个气候变化应该是必然的。他把它理解为了正确，然后就说因为没有足够的科学证据，那这后面完全就跑偏了。然后包括后面是说理论需要证据来进行测试检验，他又后面说成了理论，要以实践为基础。这一个很明显的现象就是他没有理解整句话。然后他根据自己的一个常识，然后进行来猜测。这个的话是非常不科学的。这样子不仅影响一个内容的准确度，还有一个内容的完整度，这是他突出的问题。其他同学的话基本上没有这个问题。第二句的话就是同样的应该是在一些词义的理解方面，这里concern是表示担心而不是关心，所以他的第一句话也是出现了错译现象，把它翻成了对图书馆大部分书的一个关心在继续下降。这跟原句的话是相差十万八千里了。然后被动这个地方依然没有处理出来。不过他处理的很好的是最后一句，这个延期归还书籍在未来要受到严厉处分，没有把它分成被严格执行。我觉得这个是很好的。然后汉译英部分的话，就是这个问题也是很大。因为这个同学可能我猜测他的英语的一个句子结构意识不是很强，所以会出现这里没房没车，不办婚礼，不度蜜月，只领结婚证，他全部把多个单词就处理成了一个短语，然后堆在了一块的那一种。还有包括后面一些个别词的使用，生活压力大 hard，独立性independent。然后包括这个削弱，die down这些用词都很不准确。最后一句话的这个地方也是这句话的话其实对于他来说也很难。所以导致他第一句话根本就不是一个句子了。

然后后面的话勉强是有句子的主干在，但是用词也不精准。搭乘的话，它应该是carry，但它这里用了一个cover，然后后面的fly 过去式应该是flew，这里又用了一个flow。所以他这个同学的问题，首先汉译英部分的话就是句子结构意识，他可能是比较薄弱。很多时候很多句子都没有主谓宾，甚至出现多个谓语。然后包括一个是用词的准确，这可能说明他的词汇量比较匮乏。然后英译汉部分的话就是他的理解不到位。在理解不到位的情况下，他还喜欢依据自己的常识来进行添加，所以我觉得这都是非常致命的硬伤。

十号同学汉译英部分的话，他比其他同学都显著的问题就是在于这个被字的使用。他把每一个被动带都处理成了被动。第一个被特别关注，第二个被要求，第三个被严格督促。所以说明这个同学他是受这种英语结构的迁移，在中文的表达当中真的是比较明显的，然后看起来也特别扎眼。然后另外他第一句的话甚至出现了一个错别字，威胁的威字是写错了的。然后包括这个语义的一个理解，而不是是避免不是缓解。然后他的话汉译英部分的话，其实算比他的英译汉译英要比英译汉要好。就是他的整个句子处理起来都很好，但是他第一句的话也同样存在一些很多细节问题。第一个比如说这个冠词的使用without a house，a car, 这里还有get marriage，这里也是一样的。他这里建议他重新再起一句，否则他跟上面这一句话也连接不上。然后包括后面他有看到这两个之间存在因果很好。但是后面这个因果导致的话，一般是有lead to，但是有lead to 后面又一般是加名词，但是后面又加了一个句子。其实这里就是虽然他能发现这个潜在的逻辑关系，但是当他处理到句子当中的时候，他又不太会用这说明他的一个句子的一个结构意识还是比较薄弱的。最后一句话的翻译的话，我觉得他也是跟其他同学一样，就是在于这个时态的问题。前面是就是过去式跟现在是容易混用，包括一些单词的过去式也不太准确，我觉得他处理很好的一点就是后面这几句话，向西飞过哪里什么的，这一块的话，基本上都是处理的很好。甚至最后这个冲向也是用了非谓语结构。

看完这十个同学的译文，我觉得我评分的一个标准宏观来看，首先是一个内容的一个完整性。有没有存在，是不是百分之百都传达了原句的内容，有没有漏译，还有包括他有没有增意，就比如说增添了很多不必要的信息。第二个是内容的一个准确度，就是他把这些信息都翻出来了。但是是否理解正确了，这是宏观层面。微观层面的话就是他的一些用词。首先是一个词语的一个正确的一个理解，包括一个正确的使用。然后第二个的话就是每一个句子它的一个难点部分，他们是不是都有处理出来。比如说第一句英译汉的话，他应该是在and 这个处理上面。第二个句的英译汉的话应该是在被动。第三句的话应该是在这个没房没车，不办婚礼这一块，他是要怎么样去处理的，把这些动词是要转换成名词，包括后面这个两句话的因果关系是不是有添加出来。然后最后一句的话是这个飞机上午起飞向西飞行就这一个断句的处理，就这几个得分点难点学生，他们是否有很好的完成。然后第三个部分的话就是他们的一个整体的一个句子的一个结构，就很多同学的话，他们整个句子下来是不是有完整的一个结构，在这里还有包括他一些其他的细节问题。比如说冠词的使用、时态问题这些等等的小的细节基本上也是要扣分的。所以我整个评分标准就是从宏观内容，然后微观的话就是语言层面了。

E7：

他这篇翻译有好的地方，比如说but we do not believe that climate change is a certainty，他把这里的certainty 一个名译成了动词的形式，非常简洁，然后也非常有力。不好的地方有以下几点，第一就是adverted 是避免，它译成了缓解。第二个就是prevailing theories ，他这个prevailing 理解有偏差，然后翻译的不准确，最后的话就是最后的这个句子，more evidence collected 的这边，他就是用这个被用了一个被动的语态再翻译成中文的时候，感觉不是很自然。

下面一个同学，他的整体给我的感觉就是理解很多地方理解都错了，而且错的很离谱。比如说首先就是avert it把这个避免译成扭转。第二就是后边这一句a climate change is certainty，不把他这个意思的完全译错了。然后正是因为他这边理解错，然后他把下面那一句there are no certainties in science ，也理解错了。是所以这是理解出现重大问题的两个地方。**就是最后两句话他翻译的也非常的不准确。就是我给我的感觉就是他没有以原文的这个词为基础，而是以自己的理解，凭着自己随意的理解，想当然的去翻译**。所以最后就出现了非常重大的问题。

接下来一个同学。他有一个非常好的地方，就是气候变化是一件必然事件。他把这个certainty直接翻译出来，也是翻译的非常准确的。然后就是说一说不好的地方，首先the world needs to take steps to try to avert it, 他把这个take steps采取措施翻译成一步步的去尝试改变它。感觉它对于单词这个词组的理解不到位。就是关于这个prevailing theories，可能是对于这个词不认识，然后也犯错了，还有就是关于这个理论，理论要被根据证据进行检验。那他没有翻译成检验，而翻译成这个测试也是非常直白的翻译出来，容易让人产生误解。然后这个测试这个问题出现了两次，刚才那边一次，然后最后一句话theories tested again，然后又是用了一个测试。

下面这个同学，是说一下不好的地方。这个世界是要在这个第一句的后半段，the world needs to take steps to try to avert it 他在这边对这个avert 可能不认识这个单词，所以最后就是翻译人的翻译的这个是不准确的。然后就是我们不相信气候变化是一个定数。这个定数感觉有点并不符合中文的表达习惯。还有就是最后最后一句最后一句的翻译，基本上是跟原文出入是比较大的。然后除了但虽然说有这些不好的地方，但是这个prevailing theories，他能够理解这个prevailing盛行的这个也是比较好的。前面几位同学有好几位都没有认出这个词。

接下来这个同学，整体上他的对于这个理解是比较准确的，然后基本上也都翻译出来了。那说一说，不好的地方。首先就是我们不认为气候变化具有确定性这样的一个说法很少在中文里边看到这样用，感觉不是很特别妥当。然后然后基本上没什么其他的问题了。再补充一个不好的地方。第一句，全球会带来严峻的危害，跟这个原文的严重的威胁还是有一定的偏差的。这是第一点。第二点就是危害。我们都会说严重的危害很少用严峻这个词。下面这个，他其实有一些亮点。比如说比如说就是这个a certainty ，它翻译成了板上钉钉的事实，也是比较好的一个表达。那不好的地方有以下几点，第一是一个严肃的威胁，非常的不地道。第二就是转变这一现象应该是避免这一现象。第三就是后边的几句话，这没有科学证据要验证这假说，必须由证据进行准确无误的测试。**所以这一节这一部分，他有点想当然了，就是他可能是以自己的理解为基础来进行翻译。没有原文为基础，所以最后错的很严重。**下面第二题，第一个同学，他首先第一句就是非常的贴字面，被特别关注到的是库存的急剧减少。然后最后在第二句其实也犯了同样的错误，学生们被要求时刻提醒自己，这个规定也是非常的贴字面非常的不地道。再往后再往后的话就是对最后一句话的理解出现了偏差，被严格督促这个在原文中并没有出现。他就是可能也是对这个句子不是很理解。

下面这个同学，先说不好的地方，我们担忧的注意到了，这个也是属于把直接把英文里的表达直接搬过来了，然后没有考虑到中文的表达习惯，让人听起来非常的不适。另外就是学生们被要求牢记借书还书的规则，这个被要求也是非常不妥当的。接下来就是在心里牢记其他同学的需求。他可能对于bear in mind 他的数学mind，他了解比较他的理解比较多，比较贴字面，然后就直接这样的译出来了，其实是非常不合适的，那就在心里，我觉得这可以直接去掉，就是直接牢记就可以了。但是这位同学整体的意思都出来了，还是很不错的。

下面这个同学，首先就是图书馆...的几率减少引起了明显的担忧引起了明显的担忧。在中文中也很少看到有这样的说法的。第二就是学生们被要求还是老问题了，就是这个被动，中文里边那个被动是非常少用的。接下来就是一个比较大的问题，就是最后一句话，这个penalties 它没有一它没有认出来，也没有翻译出来，在译文当中没有体现出来。下面这个同学，他的这个翻译其实整体上是非常到位的，有一些亮点。首先就是要时刻记着要为其他同学着想，他把这个bear in mind the needs of other students 翻译成了为其他同学着想，这个就比较到位，也就是能直接将原文的这个意思用中文中我们经常用的表达译出来了，这个是非常好的。另外就是他的这个句式也是小短句，没有跟一些同学一样，就是非常长。然后这样的他这样的做法把句子出一层小短句，也是符合我们中文中喜闻乐见的表达习惯的。最后一句话，对于这个逾期不还书的这个惩罚，他也非常贴心的，就是加上了主语我们然后再加上了宾语学生们，让这个意思更加的清楚。我认为这样的增译也是非常有利的。第二段，首先储书量，没有这样的说法，我们都是藏书量。第二就是就是第二句，学生们被要求提醒自己借还书的规则，首先被要求就已经非常不合适了。还有这种借还就是提醒自己这样的说法也是非常生硬的。然后就是关于最后一句话，个人延长还书理解是不到位的。我们这个原文的意思就是说这个图书预期，那个人延长还书，其实他的这个理解跟原文出入是比较大的。

下面开始第三题的点评，首先这个同学的这个翻译有一个非常大的亮点，就是最后一句话，大肆操办婚事的传统逐渐削弱，他并没有直译，而是非常巧妙的把它表表达become less popular among young people，我觉得这样的处理是非常妥当的。但是他的这个一整段话中的这些小问题是非常多的，就是说这个语法，还有这个词组的问题都比较多，都比较严重。比如说首先裸婚的翻译。第二就是a new of marrying，这个也是我们基本功不扎实。接下来就是关于这个without house car wedding，前面也没有冠词，还有这个度蜜月的这个表达也不对其他的问题倒也没有什么了。第二位其实他的问题和三位同学非常像，基本功不够扎实。首先就是对于裸婚的理解marriage without everything 这样的说法比较奇怪。比方说这个house 前面又没有冠词，后边又没有复数。就是这个办婚礼，办婚礼的说法也不对，度蜜月的说法也错了。还有就是这个领结婚证，结婚证的这个拼写也是不对的。接下来就是这个我觉得现在年轻人他这样的这样一个把他们全揉在一起，全揉在一起这样的做法也在英语中是很难被接受的。最后的大肆操办婚事的一个a big marriage 这样的说法这样的说法在是比较不地道的。还有最后一个词就是in the young general 应该是the generation。所以说这个总的来说基本功是不够到位的。但是这个逐渐但是也有还有一些亮点。就比如说这个逐渐削弱，他用了一个die down，这个确实有这样的一个意思。

下面点评下一个同学的，首先他对于裸婚的这个翻译simple wedding，我认为是可以接受的。就是一个比较简约的一种结婚方式。他的小问题也有也是基本功不够扎实。比方说they get married without wedding ceremony，前面没有冠词，然后还有一些连接词，这个连接词可能是使用不知道怎么用。比如说without wedding ceremony and honeymoon only get their certification，就only get 前面是不是要加一个连接词呢？接下来的话就是最后一句。最后一句are the tradition of huge wedding affairs，wedding affairs这样的一个说法，基本上没有没有在英语中见过。还有就是这个逐渐削弱，他用了一个被动也是不合适的。下面一个同学的第三题。首先亮点就是对于裸婚它的这个发音get married with nothing, 也是比较形象的。接下来就说一些不好的地方，首先就是他对于这个裸婚做解释的时候，用了一个indicate 这样一个词，indicate 是暗含然后是暗含的意思，跟原文它直接解释还是有区别的。接下来就是一些语法问题。比如说house 前面没有加冠词，还有一个问题，就是他非常喜欢把所有的句子都揉在一起，然后用一种非常大非常长的句子，在英语中其实也是很少见的，英语中也是比较喜欢用小而短的句子的。比如说最后一句，前面说了一大堆，然后后边就是which gradually banish tradition 什么什么什么什么就太长了，也都是不合适的。然后banish 这样这个词的使用也是有点过了，人家原文的意思是逐渐削弱，banish就直接把这个传统赶走了，把传统弄的没有了，这个力度有点太大了。接下来这个同学的第三题。首先就是裸婚。裸婚他没有想到怎么译把这个空出来了，漏译了。就是说一些语法问题，跟前面几位同学非常像，就是house前边都是没有加冠词的。然后这个car 什么的，那全部都就空着扔在这里了。还有就是最后一句话，他也是可能是不会翻，也把他漏了。但是他这里边有一个非常好的地方，有一个大的亮点，就是领证，很多同学都翻译成就是get certificate。但是他能够想到get registered 这样的一个表达也是非常地道的。接下来这个同学第三题，我认为他整体翻比较糟糕的，首先就是is a new kind of way to get married。没有这样的说法，只有a new way to get married。他就是这样的一个添加。这样的一个添加是不符合表达习惯的。就是一些基础的语法问题，house car 都都没有加冠词，然后就是这个honeymoon，然后他这个翻译不对的。还有就是现代年轻人，他这个modern use 这个就是非常机械的把这两个词组合在一起了。不符合表达习惯。就是最后一句大肆操办婚事，然后他用了这种非常复杂的几个词把它堆在一起来表达这样一个意思。我认为也是不妥当的。最后这个同学的第三题。首先就是这个卷面，给人的印象就非常不好，各种涂改。有这个问题就分别说裸婚没有翻译出来，就是a new kind of way，这个a new way 是正确的，加一个kind，做这个无谓的添加。前面有一个a了，后边这个way 他又加了一个复数，所以这体现出这个基本功能不扎实。然后就是这个领这个领证，然后pick up marriage license，这样的表达。是很少见的。但是在最后一句，我觉得他最后一句，他翻译的其实也是可以的。就是这个tradition is disappearing，就是逐渐消失。跟这个原文对应起来也是也对应的，也是比较好的。但是它的这个介词用的也不对，disappearing in young people, 这个介词用错了。

下面开始点评第四题，首先这个同学的第四题，他这个其实失误是比较多的。第一个方面就是他的主动和被动搞不清楚，就是说他注意到一家什么时候飞机他本来是一个主动的，他用成被动的。It was noticed that 这点是不对的，然后就是介词的使用也有很大的问题。比如说这个飞机7点59起飞，然后他用的是took on ，飞过这个山飞过，用应该用over 它用的through，所以介词的使用也没有掌握的很牢固。第三个方面就是一些基本的搭配，基本的用法。比方说这个飞机上载着多少名乘客，他用了一个cover。这个飞机从哪儿飞到哪儿，他用了一个flow，这个时态也是错的。所以总的来说基本功有很大的问题。下面这个同学的第四题，基本功还是比较扎实的，看看这几位同学，只有这个同学的，总体来说这个意思是比较到位的。就是在表达用法上面，也是比前面几位同学都要准确的多。但是也是有一些小问题，比如说这个波音他不知道怎么用英语表达，这个表达是错误的。然后就是时态。比如说第一句话，他前面用了，现在是后面用了过去式，然后再往后看的话，基本上就没有太大的问题了。下面这个同学的第四题。总的来说，他有一个非常好的方面，就是在介词使用上面非常的准确简洁到位。比如说从波士顿到洛杉矶的这个飞机，他他用了这个from 什么to ，然后就直接用介词代替了动词。这个飞机上多少名乘客，他直接用了一个，也是非常简单，非常地道。然后接下来说一下不好的地方，比如说一些基本功的语法问题，就是raise什么attention ，没有这样的说法。就是这个波音，波音他也不会表达，没有表达出来，然后这个飞机起飞他不会说，然后用了一个start，再往后的话，就是这个比方说这个飞机往南飞，那这个手机南南这个south之前要加一个冠词，他没有加。所以总的来说介词它的介词使用的还是不错的，但是基本功还是有些不扎实。下面这个同学的第四题。问题是有以下几个方面。首先就是这个词组搭配，引起他的注意，他用了一个caught the eyes of... , 这个是错误的。然后就是关于起飞，起飞的这个词组也没有掌握，然后用了一个非常生硬的一个表达，began flying at...。就是一些介词，介词掌握的也不够好。比如说kept flying a head west，对吧？这里边出现两个问题，一个是用的这个ahead ，然后另外一个就是这个west 前面需要加一个冠词，接下来就是时态。比如说这个fly这个词过去式的话是flew，它用它是用了一个flied。所以这也就体现出它的时态以及所有的基本功方面掌握的不够扎实。最后这个同学，我认为一个比较好的地方比较好的一个地方就是他能够在不会表达某些意思的时候，他不会拘泥于某一些字词。就比如说一架波音767型飞机，它不会表达，但是他能够将这个意思传达出来。用了一个a plane 来代替这样一个意思。所以虽然说它的这个词具体的词它不会表达，但是意思被他译出来了，我认为这一点还是比较到位的。接下来说一下不足的地方，首先就是这个词语的搭配这个came into 他的eyes，这个是没有这样的表达的。就是这个起飞took off，他用了take up了。然后还有第一句的就是这个firstly 应该用first。然后再往后的话，基本上没有其他的问题了。
