# Supplementary material for: Assessing Students’ Translation Competence: Integrating China’s Standards of English With Cognitive Diagnostic Assessment Approaches
Source: Front Psychol. 2022 Mar 31;13:872025. doi: 10.3389/fpsyg.2022.872025 (PMC9008137; doi:10.3389/fpsyg.2022.872025)
Supplement: Supplementary file 5 [file Data_Sheet_5.DOCX]

Supplementary Material (Q-matrix coding results of 7 experts )

E1:

| Attribute  Descriptor | A1 | A2 | A3 | A4 | A5 | A6 | A7 |
| --- | --- | --- | --- | --- | --- | --- | --- |
| D1 | 1 | 0 | 0 | 0 | 0 | 0 | 0 |
| D2 | 0 | 1 | 0 | 0 | 0 | 0 | 0 |
| D3 | 0 | 1 | 0 | 0 | 1 | 0 | 1 |
| D4 | 1 | 0 | 0 | 0 | 0 | 0 | 0 |
| D5 | 0 | 1 | 0 | 0 | 0 | 0 | 0 |
| D6 | 0 | 0 | 0 | 1 | 0 | 0 | 1 |
| D7 | 0 | 1 | 0 | 1 | 1 | 0 | 1 |
| D8 | 0 | 1 | 0 | 0 | 0 | 0 | 1 |
| D9 | 1 | 0 | 0 | 0 | 0 | 0 | 0 |
| D10 | 0 | 1 | 0 | 0 | 0 | 0 | 0 |
| D11 | 0 | 0 | 1 | 0 | 0 | 0 | 0 |
| D12 | 0 | 0 | 0 | 1 | 0 | 1 | 1 |
| D13 | 0 | 0 | 0 | 0 | 0 | 1 | 1 |
| D14 | 0 | 0 | 0 | 1 | 0 | 1 | 1 |
| D15 | 1 | 0 | 0 | 0 | 0 | 0 | 0 |
| D16 | 0 | 1 | 0 | 0 | 0 | 0 | 0 |
| D17 | 0 | 1 | 1 | 0 | 0 | 0 | 1 |
| D18 | 0 | 0 | 1 | 0 | 0 | 0 | 0 |
| D19 | 0 | 0 | 0 | 0 | 1 | 0 | 0 |
| D20 | 0 | 0 | 0 | 1 | 0 | 1 | 1 |

E2:

| Attribute  Descriptor | A1 | A2 | A3 | A4 | A5 | A6 | A7 |
| --- | --- | --- | --- | --- | --- | --- | --- |
| D1 | 1 | 0 | 0 | 0 | 0 | 0 | 0 |
| D2 | 0 | 1 | 0 | 0 | 0 | 0 | 0 |
| D3 | 0 | 0 | 0 | 0 | 1 | 0 | 1 |
| D4 | 1 | 0 | 0 | 0 | 0 | 0 | 0 |
| D5 | 0 | 1 | 0 | 0 | 0 | 0 | 0 |
| D6 | 0 | 0 | 0 | 1 | 0 | 0 | 1 |
| D7 | 0 | 0 | 0 | 1 | 1 | 0 | 1 |
| D8 | 0 | 0 | 0 | 0 | 0 | 0 | 1 |
| D9 | 1 | 0 | 0 | 0 | 0 | 0 | 0 |
| D10 | 0 | 1 | 0 | 0 | 0 | 0 | 0 |
| D11 | 0 | 0 | 1 | 0 | 0 | 0 | 0 |
| D12 | 0 | 0 | 0 | 1 | 0 | 1 | 1 |
| D13 | 0 | 0 | 1 | 0 | 0 | 1 | 1 |
| D14 | 0 | 0 | 0 | 1 | 0 | 1 | 1 |
| D15 | 1 | 0 | 0 | 0 | 0 | 0 | 0 |
| D16 | 0 | 1 | 0 | 0 | 0 | 0 | 0 |
| D17 | 0 | 0 | 1 | 0 | 0 | 0 | 1 |
| D18 | 0 | 0 | 1 | 0 | 0 | 0 | 0 |
| D19 | 0 | 0 | 0 | 0 | 1 | 0 | 0 |
| D20 | 0 | 0 | 0 | 1 | 0 | 1 | 1 |

E3:

| Attribute  Descriptor | A1 | A2 | A3 | A4 | A5 | A6 | A7 |
| --- | --- | --- | --- | --- | --- | --- | --- |
| D1 | 1 | 0 | 0 | 0 | 0 | 0 | 0 |
| D2 | 0 | 1 | 0 | 0 | 0 | 0 | 0 |
| D3 | 0 | 0 | 0 | 0 | 1 | 1 | 1 |
| D4 | 1 | 0 | 0 | 0 | 0 | 0 | 0 |
| D5 | 0 | 1 | 0 | 0 | 0 | 0 | 0 |
| D6 | 0 | 0 | 0 | 1 | 0 | 0 | 1 |
| D7 | 0 | 0 | 0 | 1 | 1 | 1 | 1 |
| D8 | 0 | 1 | 0 | 0 | 0 | 0 | 1 |
| D9 | 1 | 0 | 0 | 0 | 0 | 0 | 0 |
| D10 | 0 | 1 | 0 | 0 | 0 | 0 | 0 |
| D11 | 0 | 0 | 1 | 0 | 0 | 0 | 0 |
| D12 | 0 | 0 | 0 | 1 | 0 | 0 | 1 |
| D13 | 0 | 0 | 1 | 1 | 0 | 1 | 1 |
| D14 | 0 | 0 | 0 | 1 | 0 | 1 | 1 |
| D15 | 1 | 0 | 0 | 0 | 0 | 0 | 0 |
| D16 | 0 | 1 | 0 | 0 | 0 | 0 | 0 |
| D17 | 0 | 1 | 0 | 0 | 0 | 0 | 1 |
| D18 | 0 | 0 | 1 | 0 | 0 | 0 | 0 |
| D19 | 0 | 0 | 0 | 0 | 1 | 0 | 0 |
| D20 | 0 | 0 | 0 | 1 | 0 | 1 | 1 |

E4:

| Attribute  Descriptor | A1 | A2 | A3 | A4 | A5 | A6 | A7 |
| --- | --- | --- | --- | --- | --- | --- | --- |
| D1 | 1 | 0 | 0 | 0 | 0 | 0 | 0 |
| D2 | 0 | 1 | 0 | 0 | 0 | 0 | 0 |
| D3 | 0 | 1 | 0 | 0 | 1 | 0 | 1 |
| D4 | 1 | 0 | 0 | 0 | 0 | 0 | 0 |
| D5 | 0 | 1 | 0 | 0 | 0 | 0 | 0 |
| D6 | 0 | 0 | 0 | 1 | 0 | 0 | 1 |
| D7 | 0 | 1 | 0 | 1 | 1 | 0 | 1 |
| D8 | 0 | 1 | 0 | 0 | 0 | 0 | 1 |
| D9 | 1 | 0 | 0 | 0 | 0 | 0 | 0 |
| D10 | 0 | 1 | 0 | 0 | 0 | 0 | 0 |
| D11 | 0 | 0 | 1 | 0 | 0 | 0 | 0 |
| D12 | 0 | 0 | 0 | 1 | 0 | 1 | 1 |
| D13 | 0 | 0 | 1 | 1 | 0 | 0 | 1 |
| D14 | 0 | 0 | 0 | 1 | 0 | 1 | 1 |
| D15 | 1 | 0 | 0 | 0 | 0 | 0 | 0 |
| D16 | 0 | 1 | 0 | 0 | 0 | 0 | 0 |
| D17 | 0 | 0 | 0 | 0 | 0 | 0 | 1 |
| D18 | 0 | 0 | 1 | 0 | 0 | 0 | 0 |
| D19 | 0 | 0 | 0 | 0 | 1 | 0 | 0 |
| D20 | 0 | 0 | 0 | 1 | 0 | 1 | 1 |

E5:

| Attribute  Descriptor | A1 | A2 | A3 | A4 | A5 | A6 | A7 |
| --- | --- | --- | --- | --- | --- | --- | --- |
| D1 | 1 | 0 | 0 | 0 | 0 | 0 | 0 |
| D2 | 0 | 1 | 0 | 0 | 0 | 0 | 0 |
| D3 | 0 | 0 | 0 | 0 | 1 | 0 | 1 |
| D4 | 1 | 0 | 0 | 0 | 0 | 0 | 0 |
| D5 | 0 | 1 | 0 | 0 | 0 | 0 | 0 |
| D6 | 0 | 0 | 0 | 1 | 0 | 0 | 1 |
| D7 | 0 | 0 | 0 | 1 | 1 | 0 | 1 |
| D8 | 0 | 0 | 0 | 0 | 0 | 0 | 1 |
| D9 | 1 | 0 | 0 | 0 | 0 | 0 | 0 |
| D10 | 0 | 1 | 0 | 0 | 0 | 0 | 0 |
| D11 | 0 | 0 | 1 | 0 | 0 | 0 | 0 |
| D12 | 0 | 0 | 0 | 1 | 0 | 0 | 1 |
| D13 | 0 | 0 | 1 | 1 | 0 | 0 | 1 |
| D14 | 0 | 0 | 0 | 1 | 0 | 1 | 1 |
| D15 | 1 | 0 | 0 | 0 | 0 | 0 | 0 |
| D16 | 0 | 1 | 0 | 0 | 0 | 0 | 0 |
| D17 | 0 | 0 | 1 | 0 | 0 | 0 | 1 |
| D18 | 0 | 0 | 1 | 0 | 0 | 0 | 0 |
| D19 | 0 | 1 | 0 | 0 | 1 | 0 | 0 |
| D20 | 0 | 0 | 0 | 1 | 0 | 1 | 1 |

E6:

| Attribute  Descriptor | A1 | A2 | A3 | A4 | A5 | A6 | A7 |
| --- | --- | --- | --- | --- | --- | --- | --- |
| D1 | 1 | 0 | 0 | 0 | 0 | 0 | 0 |
| D2 | 0 | 1 | 0 | 0 | 0 | 0 | 0 |
| D3 | 0 | 0 | 0 | 0 | 1 | 0 | 1 |
| D4 | 1 | 0 | 0 | 0 | 0 | 0 | 0 |
| D5 | 0 | 1 | 0 | 0 | 0 | 0 | 0 |
| D6 | 0 | 0 | 0 | 1 | 0 | 0 | 1 |
| D7 | 0 | 0 | 0 | 1 | 1 | 0 | 1 |
| D8 | 0 | 1 | 0 | 0 | 0 | 1 | 1 |
| D9 | 1 | 0 | 0 | 0 | 0 | 0 | 0 |
| D10 | 0 | 1 | 0 | 0 | 0 | 0 | 0 |
| D11 | 0 | 0 | 1 | 0 | 0 | 0 | 0 |
| D12 | 0 | 0 | 0 | 1 | 0 | 1 | 1 |
| D13 | 0 | 0 | 0 | 0 | 0 | 1 | 1 |
| D14 | 0 | 0 | 0 | 1 | 0 | 1 | 1 |
| D15 | 1 | 0 | 0 | 0 | 0 | 0 | 0 |
| D16 | 0 | 1 | 0 | 0 | 0 | 0 | 0 |
| D17 | 0 | 0 | 1 | 0 | 0 | 0 | 1 |
| D18 | 0 | 0 | 1 | 0 | 0 | 0 | 0 |
| D19 | 0 | 0 | 0 | 0 | 1 | 0 | 0 |
| D20 | 0 | 0 | 0 | 1 | 0 | 1 | 1 |

E7:

| Attribute  Descriptor | A1 | A2 | A3 | A4 | A5 | A6 | A7 |
| --- | --- | --- | --- | --- | --- | --- | --- |
| D1 | 1 | 0 | 0 | 0 | 0 | 0 | 0 |
| D2 | 0 | 1 | 0 | 0 | 0 | 0 | 0 |
| D3 | 0 | 0 | 0 | 0 | 1 | 0 | 1 |
| D4 | 1 | 0 | 0 | 0 | 0 | 0 | 0 |
| D5 | 0 | 1 | 0 | 0 | 0 | 0 | 0 |
| D6 | 0 | 0 | 0 | 1 | 0 | 0 | 1 |
| D7 | 0 | 0 | 0 | 0 | 1 | 0 | 1 |
| D8 | 0 | 1 | 0 | 0 | 0 | 0 | 1 |
| D9 | 1 | 0 | 0 | 0 | 0 | 0 | 0 |
| D10 | 0 | 1 | 0 | 0 | 0 | 0 | 0 |
| D11 | 0 | 0 | 1 | 0 | 0 | 0 | 0 |
| D12 | 0 | 0 | 0 | 1 | 0 | 1 | 1 |
| D13 | 0 | 0 | 0 | 1 | 0 | 1 | 1 |
| D14 | 0 | 0 | 1 | 1 | 0 | 1 | 1 |
| D15 | 1 | 0 | 0 | 0 | 0 | 0 | 0 |
| D16 | 0 | 1 | 0 | 0 | 0 | 0 | 0 |
| D17 | 0 | 0 | 1 | 0 | 0 | 0 | 1 |
| D18 | 0 | 0 | 1 | 0 | 0 | 0 | 0 |
| D19 | 0 | 0 | 0 | 0 | 1 | 0 | 0 |
| D20 | 0 | 0 | 0 | 1 | 1 | 1 | 1 |
